# Glucocorticoid receptor-dependent therapeutic efficacy of tauroursodeoxycholic acid in preclinical models of spinocerebellar ataxia type 3

**DOI:** 10.1172/JCI162246

**Published:** 2024-03-01

**Authors:** Sara Duarte-Silva, Jorge Diogo Da Silva, Daniela Monteiro-Fernandes, Marta Daniela Costa, Andreia Neves-Carvalho, Mafalda Raposo, Carina Soares-Cunha, Joana S. Correia, Gonçalo Nogueira-Goncalves, Henrique S. Fernandes, Stephanie Oliveira, Ana Rita Ferreira-Fernandes, Fernando Rodrigues, Joana Pereira-Sousa, Daniela Vilasboas-Campos, Sara Guerreiro, Jonas Campos, Liliana Meireles-Costa, Cecilia M.P. Rodrigues, Stephanie Cabantous, Sergio F. Sousa, Manuela Lima, Andreia Teixeira-Castro, Patricia Maciel

**Affiliations:** 1 Life and Health Sciences Research Institute (ICVS), School of Medicine, University of Minho, Braga, Portugal.; 2 ICVS/3B’s – PT Government Associate Laboratory, Braga/Guimarães, Portugal.; 3 Medical Genetics Center Dr. Jacinto de Magalhães, Santo António University Hospital Center, Porto, Portugal.; 4 Unit for Multidisciplinary Research in Biomedicine, Abel Salazar Biomedical Sciences Institute, Porto University, Porto, Portugal.; 5 Instituto de Biologia Molecular e Celular (IBMC), Instituto de Investigação e Inovação em Saúde (i3S), Universidade do Porto, Porto, Portugal.; 6 Faculdade de Ciências e Tecnologia, Universidade dos Açores, Ponta Delgada, Portugal.; 7 UCIBIO – Applied Molecular Biosciences Unit, BioSIM – Departamento de Biomedicina and; 8 Associate Laboratory i4HB – Institute for Health and Bioeconomy, Faculdade de Medicina, Universidade do Porto, Porto, Portugal.; 9 Research Institute for Medicines (iMed.ULisboa), Faculty of Pharmacy, Universidade de Lisboa, Lisbon, Portugal.; 10 Cancer Research Center of Toulouse (CRCT), Inserm, Université de Toulouse, UPS, CNRS, Toulouse, France.

**Keywords:** Neuroscience, Therapeutics, Drug therapy, Genetic diseases, Molecular biology

## Abstract

Spinocerebellar ataxia type 3 (SCA3) is an adult-onset neurodegenerative disease caused by a polyglutamine expansion in the ataxin-3 (*ATXN3*) gene. No effective treatment is available for this disorder, other than symptom-directed approaches. Bile acids have shown therapeutic efficacy in neurodegenerative disease models. Here, we pinpointed tauroursodeoxycholic acid (TUDCA) as an efficient therapeutic, improving the motor and neuropathological phenotype of SCA3 nematode and mouse models. Surprisingly, transcriptomic and functional in vivo data showed that TUDCA acts in neuronal tissue through the glucocorticoid receptor (GR), but independently of its canonical receptor, the farnesoid X receptor (FXR). TUDCA was predicted to bind to the GR, in a similar fashion to corticosteroid molecules. GR levels were decreased in disease-affected brain regions, likely due to increased protein degradation as a consequence of ATXN3 dysfunction being restored by TUDCA treatment. Analysis of a SCA3 clinical cohort showed intriguing correlations between the peripheral expression of *GR* and the predicted age at disease onset in presymptomatic subjects and *FKBP5* expression with disease progression, suggesting this pathway as a potential source of biomarkers for future study. We have established a novel in vivo mechanism for the neuroprotective effects of TUDCA in SCA3 and propose this readily available drug for clinical trials in SCA3 patients.

## Abstract 2

Glucocorticoid receptor dysfunction is a pathophysiologic mechanism in Spinocerebellar ataxia type 3 that can be restored using a bile acid therapy in mice.

## Introduction

Neurodegenerative diseases are currently considered a growing epidemic in the aging population (1, 2), still lacking effective therapeutic options. For those disorders with an identified genetic cause, research has been focused on gene therapy approaches, with mixed outcomes (3, 4). Importantly, technical and ethical questions regarding these strategies are likely to hinder and delay their use in the near future (5, 6). Therefore, other lines of research should, perhaps, focus on complementary or alternative approaches that may be more feasible. One of these is the repurposing of currently approved drugs for treatment of different disorders, an approach that could significantly and rapidly change how neurodegenerative genetic disorders are being managed (7, 8).

Spinocerebellar ataxia type 3 (SCA3), also known as Machado-Joseph disease (MJD), is the most common autosomal dominant ataxia worldwide and is caused by the expansion of a CAG repeat in the *ATXN3* gene (9, 10). The resulting protein harbors an abnormally large polyglutamine (polyQ) tract, which renders it neurotoxic and prone to form aggregates (11). While loss of ATXN3 does not explain the pathogenesis of SCA3 by itself, the interference of the expanded form with interacting proteins and their respective functions (in a dominant negative manner) can be highly deleterious (12–15). Interestingly, while native ATXN3 acts as a deubiquitylase (DUB) that functions in several protein quality control processes, its expanded form disturbs proteostasis networks (16, 17).

Clinically, SCA3 is one of the most heterogeneous ataxias, with an onset varying between young and middle adulthood (18, 19) and with variable system involvement. The most common presenting symptom is gait abnormality, frequently accompanied by oculomotor and speech difficulties (18). Additional manifestations are spasticity, amyotrophy, peripheral neuropathy, and extrapyramidal symptoms, usually without cognitive impairment (10, 20). Imaging studies reveal pontocerebellar atrophy, dilation of the fourth ventricle as well as basal ganglia abnormalities (21). The clinical presentation reflects the neuropathological findings in SCA3, the main affected areas being the deep cerebellar nuclei (DCN), pontine nuclei, anterior horn of the spinal cord, cranial nerves III to XII, *substantia nigra*, and several thalamic and other brainstem nuclei (11, 14). While intranuclear inclusions of aggregated ATXN3 in neurons are a hallmark of SCA3 pathology, they are present in both affected and nonaffected areas, contradicting the idea that these large aggregates are the key disease-causing entities in neurons (11, 22). Moreover, astrogliosis and microgliosis are often present in patients with SCA3 (23, 24).

Development of therapies for SCA3 is ongoing in multiple directions (25, 26). Several studies have shown that silencing the expanded form of ATXN3 or countering downstream protein dyshomeostasis are effective therapeutic approaches (27–32), but little has translated to the clinics so far (33). The current management of SCA3 is undirected and symptom-focused, with limited impact, given the lack of approaches that reduce or stop disease progression (34).

Bile acids (BAs) are steroid molecules synthesized in hepatocytes, being subsequently released to the gastrointestinal (GI) tract to emulsify and allow absorption of dietary lipids (35). In addition to their role in digestion, they are essential for liver homeostasis, functioning as signaling molecules that act through nuclear hormone receptors (NHRs) — most commonly through the farnesoid X receptor (FXR) (36) — in response to increased levels of BAs, as an autoregulation mechanism. These processes are clinically relevant, ursodeoxycholic acid (UDCA) being the first-line therapy in primary biliary cholangitis and an adjuvant therapy in several other hepatic and biliary disorders (37).

Contrary to the knowledge on BAs in the digestive system, their relevance to the nervous system is unclear (38). Previous studies have identified the presence of BAs in the brain (39) and even hypothesized that they could be endogenously synthesized, since Cyp27a1 expression was detected (40). Considering a possible role of BAs as endogenous neurosteroids, these molecules have been put forward as potential therapies for Alzheimer’s, Parkinson’s, and Huntington’s diseases, as well as for amyotrophic lateral sclerosis (41–45). UDCA and tauroursodeoxycholic acid (TUDCA) were shown to reduce neuronal apoptosis by stabilizing the unfolding protein response and inhibiting radical oxygen species formation (46). Whether these downstream effects were mediated through nuclear steroid receptor activation (the canonical signaling pathway) is still unclear. In vitro studies showed that, in addition to the FXR, BAs could also act through the glucocorticoid receptor (GR) (46) as well as through the G-protein coupled receptor GPBAR1/TGR5 (47).

Since BAs were shown to be protective in other neurodegenerative disorders associated with impaired proteostasis, we have hypothesized that these could be possible therapeutics for SCA3, which would be attractive due to their elevated translational potential. In this work we used a *Caenorhabditis elegans* model of SCA3 (17) to screen several BAs for their therapeutic potential. After identifying TUDCA as the most efficient molecule, we performed a preclinical trial in a mouse model of the disease (CMVMJD135) (48), which showed TUDCA as inducing a strong phenotype reversal. A followup pathomolecular characterization revealed neuroprotection by TUDCA treatment. We functionally show that these positive effects are independent of FXR and dependent on neuronal GR, as suggested by transcriptomic and pharmacological approaches in nematode and mouse models of SCA3. We also observed that ATXN3 interacts with GR and that TUDCA directs this interaction toward the nucleus. Additionally, our results suggest that GR protein degradation is increased in diseased mice and is prevented by TUDCA administration. Finally, we show intriguing correlations of peripheral *GR* and *FKBP5* mRNA expression with the predicted age of clinical conversion and with disease progression, respectively, in a SCA3 clinical cohort. Hence, we propose what we believe to be a novel mechanism of action for this neurosteroid and suggest TUDCA as a promising drug to treat SCA3, targeting GR dysfunction, that can be a clue for candidate biomarkers of disease,to be further studied at the clinical level.

## Results

### TUDCA is the most efficient BA at improving the SCA3 phenotype.

**Figure 1:**
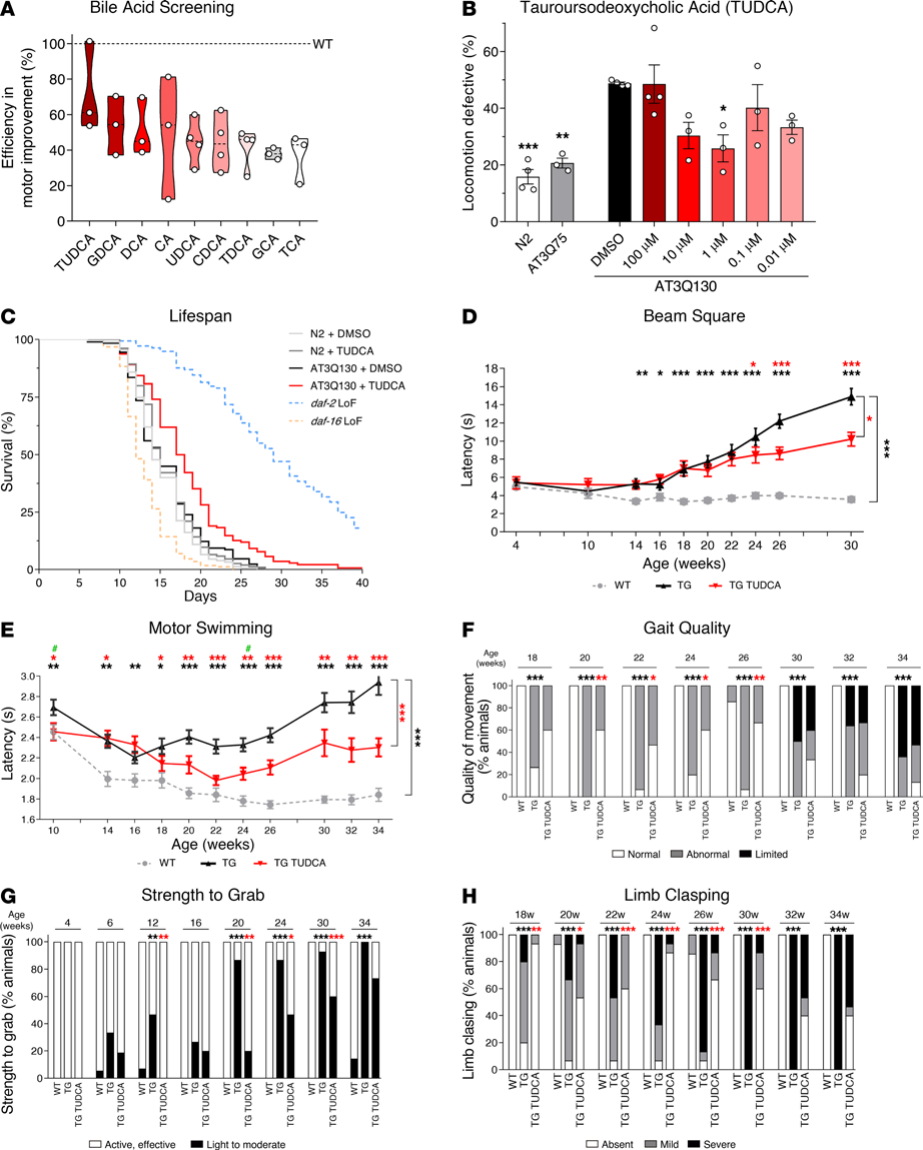
Impact of BAs on the motor phenotype of SCA3 nematode and mouse models. (**A**) Percentage of improvement in locomotion-defective animals of a representative set of BAs. The predetermined optimal concentration for each compound was used. A total of 3-to-4 independent experiments were performed, for a total of 150–200 animals assayed. (**B**) Dose-response evaluation of the effect of TUDCA in reducing the percentage of locomotion-defective AT3Q130 animals. AT3Q75 animals were used as a TG non-motor-defective control, and 1% DMSO was used as the negative control for vehicle administration. A total of 3-to-4 independent experiments with 150–200 animals were performed. (**C**) Kaplan-Meyer curve of the survival of AT3Q130 nematodes treated with TUDCA. The *daf-2* and *daf-16* strains were used as long and short-lived controls, respectively. A total of 300 animals per condition were tested along 3 independent experiments. (**D**) Evaluation of the time taken for a mouse to cross a 12 mm square beam, with TUDCA having a positive effect. (**E**) Assessment of the time taken for a mouse to swim through a 60 cm water path. TUDCA consistently improved this time throughout the trial. #, no difference between WT and TG TUDCA animals. (**F**) Gait quality was improved in treated animals. (**G**) The strength of an animal to grab a grid was used to evaluate forelimb strength. TUDCA had a positive effect in some time points. (**H**) Qualitative assessment of limb clasping revealed a significant improvement of TG TUDCA-treated animals in comparison with TG. Black *, WT versus TG; red *, TG versus TG TUDCA. A total of 14–17 mice per condition was used in all tests and evaluated in the indicated weeks of age. 1-way ANOVA (**B**, **D**, and **E**). Kaplan Meier and Cox regression (**C**). κ^2^ test (**F**–**H**). * *P* < 0.05, ** *P* < 0.01, *** *P* < 0.001.

Previous studies have pointed to the usage of BAs as potential candidates for the treatment of neurodegenerative diseases. Therefore, we started by evaluating the effect of several BA molecules in a well-established *C*. *elegans* model of SCA3, which expresses human ATXN3 with 130 glutamine residues (AT3Q130) (17). After confirming that all candidate compounds were nontoxic to WT animals (Supplemental Figure 1; supplemental material available online with this article; https://doi.org/10.1172/JCI162246DS1), we tested their effect, at several concentrations (0.0001–50 μM), on the motor phenotype of AT3Q130 animals (Supplemental Figure 2). A total of 6 of 9 of the tested BAs had a beneficial effect in at least one of the concentrations assessed (CA, DCA, GCA, GDCA, TDCA, and TUDCA). After selecting the concentration with the highest positive effect for each individual molecule, we determined that the efficiency in recovering locomotion defects was highest with TUDCA treatment at a concentration of 1 μM (Figure 1, A and B). Moreover, TUDCA was able to significantly increase the lifespan of AT3Q130 animals (Figure 1C and Supplemental Table 2) by 13% (the median survival of the AT3Q130 strain being 15 days, that of TUDCA-treated animals was 17 days), supporting its therapeutic potential.

Next, using the CMVMJD135 transgenic (TG) mouse model of SCA3, which recapitulates several key aspects of the human disease (48), we performed a preclinical trial to test the effect of TUDCA on motor impairment (Supplemental Figure 3A). An initial evaluation of motor function was carried out at 4 weeks of age (presymptomatic stage) and treatment was started at 5 weeks by supplementing food with 0.4% TUDCA (w/w). We confirmed that the mean food consumption was similar between diets (Supplemental Figure 3B) and that serum levels of TUDCA were significantly increased in animals fed a TUDCA-supplemented diet (Supplemental Figure 3C). No signs of toxicity or secondary effects of TUDCA were detected throughout the study. The CAG repeat number, which is known to correlate directly with disease severity and inversely with age of symptom onset in this disease, was similar between untreated and treated mice (Supplemental Figure 3D), confirming an adequate matching of the experimental groups for this potential confounder.

TUDCA was able to ameliorate several early onset motor phenotypes of CMVMJD135 mice, such as deficits in the beam walk (Figure 1D) and motor swimming tests (Figure 1E and Supplemental Videos 1 and 2), and increased the age at which animals were able to complete the beam test (Supplemental Figure 4A). This was not due to weight variation, with TUDCA having no effect on body weight (Supplemental Figure 3E). Motor deficits regarding both gait (Figure 1F and Supplemental Figure 4C) and limb strength (Figure 1G and Supplemental Figure 4D) were also largely improved upon treatment. While TUDCA had little effect on the performance in the hanging wire test (Supplemental Figure 4B), a recurrent observation in positive preclinical trials in this model (27, 48), it was able to strongly improve late-onset phenotypes of limb clasping (Figure 1H) and tremors (Supplemental Figure 4E). In spite of a mild effect of TUDCA on the vertical exploratory activity of SCA3 mice (Supplemental Figure 4F), it was noticeable that, at 34 weeks of age, treated animals (but not untreated) were still able to hold with their hindlimb paws.

Importantly, none of the tested behavioral patterns of WT animals treated with TUDCA were different from untreated WT animals (Supplemental Table 3), which strongly supports the safety and specificity of the compound for the disease context. In summary, chronic and early administration of TUDCA led to an overall improvement of the motor behavior in the CMVMJD135 mouse model, confirming its potential for SCA3 treatment.

### Neuropathological findings in SCA3 mice are ameliorated by TUDCA.

**Figure 2:**
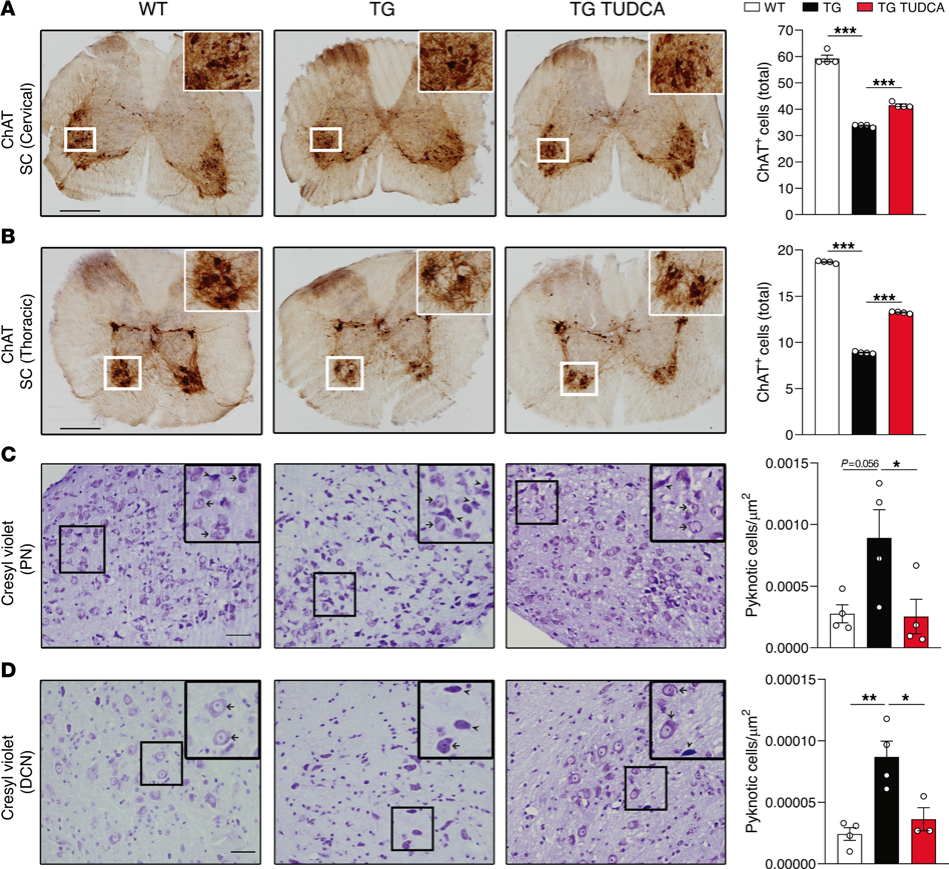
TUDCA reduces neuropathology in CMVMJD135 mice. (**A**) IHC (and respective quantification) of ChAT-positive cells in the cervical and (**B**) thoracic regions of the ventral horn of the spinal cord. (**C**) Pyknotic cells per area were counted after cresyl violet staining in the pontine nuclei and (**D**) DCN of mice. All analyses were performed in 34-week-old mouse tissue in a minimum of 3 animals per group. 1-Way ANOVA, * *P* < 0.05, ** *P* < 0.01, *** *P* < 0.001. Scale bars: 200 μm (**A** and **B**), 50 μm (**C** and **D**).

Widespread histological changes in the CNS have been described in several mouse models of SCA3 (29, 49, 50). Treatment with TUDCA prevented loss of cholinergic motor neurons in the cervical and thoracic spinal cord of CMVMJD135 mice (Figure 2, A and B) and normalized the number of pyknotic cells in pontine (Figure 2C) and DCN (Figure 2D). Despite this beneficial effect on key regions for the disease, TUDCA did not normalize the gross brain weight of SCA3 mice (Supplemental Figure 3F); this measure, however, completely excludes the spinal cord, where most of the beneficial impact on neuropathology was observed. Interestingly, this neuroprotective effect was dissociated from an impact on ATXN3 aggregation, as TUDCA had no effect in the number of intranuclear inclusions in the lateral reticular and pontine nuclei (Supplemental Figure 5, A and B, respectively). In conclusion, not only was TUDCA able to significantly improve motor phenotypes, but it also ameliorated different aspects of neuropathology, independently of direct effects on ATXN3 aggregation.

### TUDCA promotes neuroinflammatory homeostasis in SCA3 mice.

Having established the therapeutic potential of TUDCA, we shifted our focus to the molecular and cellular effects of this drug. Since proteostasis imbalance is a hallmark of SCA3 (16), we evaluated several branches of the protein control quality control for their response to TUDCA and no changes were found in gene expression/protein levels of key components of the heat shock and mitochondrial unfolded protein responses (Supplemental Figure 6A), antioxidant response (Supplemental Figure 6B), ER stress response (Supplemental Figure 6C), autophagy (Supplemental Figure 6D), or the proteasome (Supplemental Figure 6E) in the brainstem of 34-week-old animals.

**Figure 3:**
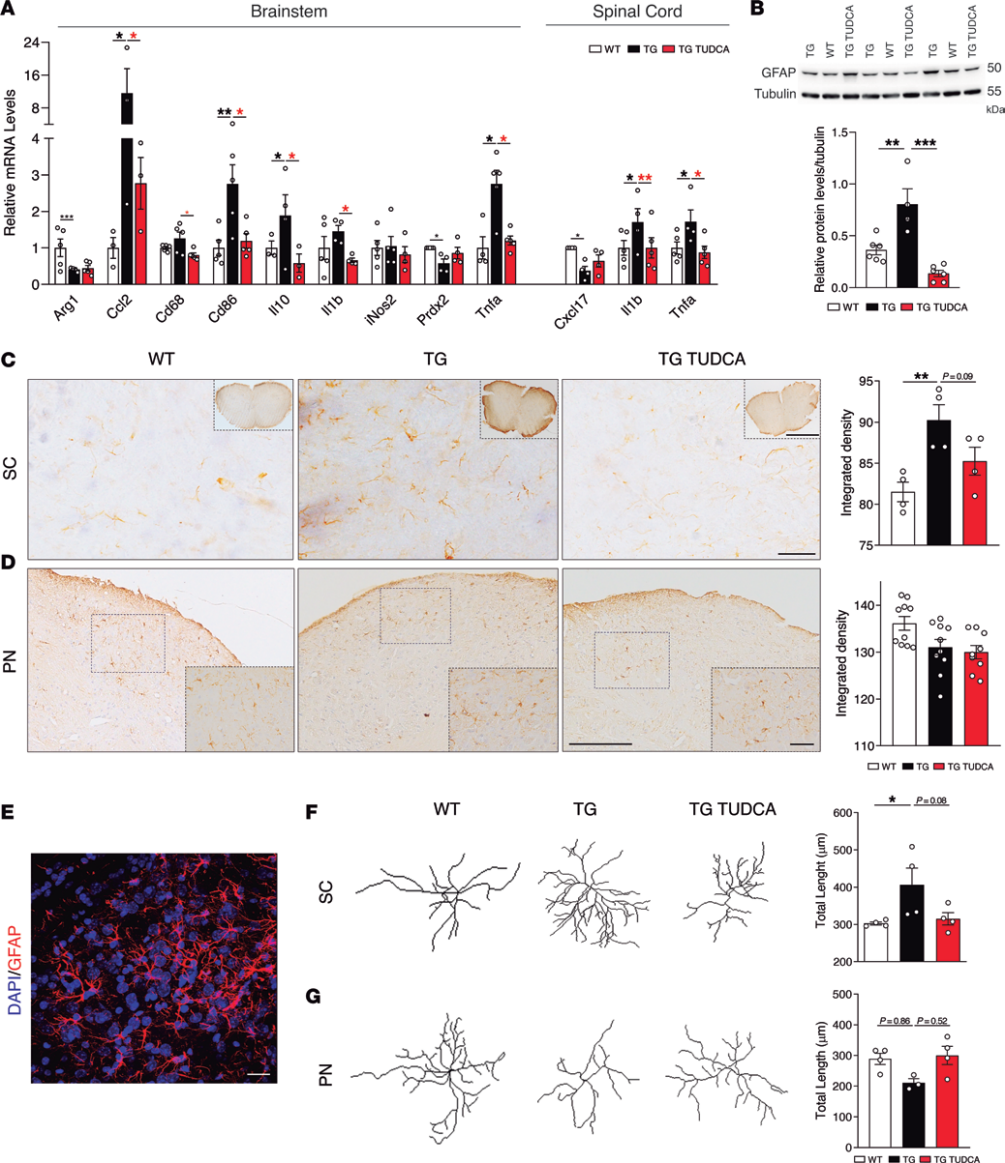
TUDCA reduces neuroinflammation in CMVMJD135 mice. (**A**) RT-qPCR of brainstem and spinal cord tissue of 34-week-old mice, with expression values normalized for β-2-microglobumin (B2m). A total of 4–6 biological replicates were evaluated. (**B**) Western blot analysis of glial fibrillary acidic protein (GFAP) levels, normalized to α-tubulin, in the brainstem of 34-week-old mice. A total of 4–6 biological replicates was tested. (**C**) IHC analysis of GFAP staining (and respective quantification) in the spinal cord (SC) and (**D**) pontine nuclei of 34-week-old mice. A total of 4–6 animals was analyzed per group. Scale bars: 50 μm (higher magnification) and 200 μm (lower magnification) (**C** and **D**). (**E**) Representative microphotograph of GFAP staining (nuclei stained by DAPI) in the spinal cord of WT mice. Scale bar: 25μm. (**F**) Representative image of skeletonized astrocytes for morphological evaluation of process complexity in the cervical spinal cord (SC) and (**G**) pontine nuclei of 34-week-old mice. A minimum number of 3 animals was used per condition, with 15–32 astrocytes being analyzed per group. Black *, WT versus TG; red *, TG versus TG TUDCA. 1-Way ANOVA, * *P* < 0.05, ** *P* < 0.01, *** *P* < 0.001.

Since TUDCA has been classically described as an antiinflammatory and antiapoptotic molecule (46), we evaluated both processes in CMVMJD135 mice. TUDCA was able to normalize the expression of several anti and proinflammatory markers in the brainstem and spinal cord of 34-week-old mice (Figure 3A and Supplemental Figure 7A), suggesting promotion of immunological homeostasis. Moreover, TUDCA treatment was able to revert astrocytic reactivity, measured by protein levels of glial fibrillary acid protein (GFAP) in the brainstem (Figure 3B), and GFAP staining in the spinal cord (Figure 3C). No changes were observed in GFAP staining levels in the pontine nuclei (Figure 3D) in all groups. However, TUDCA showed a tendency to normalize astrocyte morphology changes observed in the spinal cord and pontine nuclei of TG mice (Figure 3, E–G). No differences were observed in the protein levels of the microglia marker IBA-1 (Supplemental Figure 7B), IKKβ (Supplemental Figure 7C), phosphorylated NF-κB p65 (Supplemental Figure 7D), protein levels of TP53 (Supplemental Figure 7E), or expression of caspase 3 (Supplemental Figure 7F), either in the model or upon treatment. In conclusion, CMVMJD135 mice show some evidence of neuroinflammatory dyshomeostasis, mostly at late disease stages, that is effectively countered by TUDCA.

### The neuroprotective effect of TUDCA is GR-dependent and FXR-independent.

In an attempt to define TUDCA’s mechanism of action, we wanted to assess whether the canonical BA receptor FXR (35) was required for the effect of TUDCA in the brain. Moreover, looking at possible alternative receptors, TUDCA has also been shown to interact with the GR in vitro and to require this receptor for some of its cellular effects (46).

**Figure 4:**
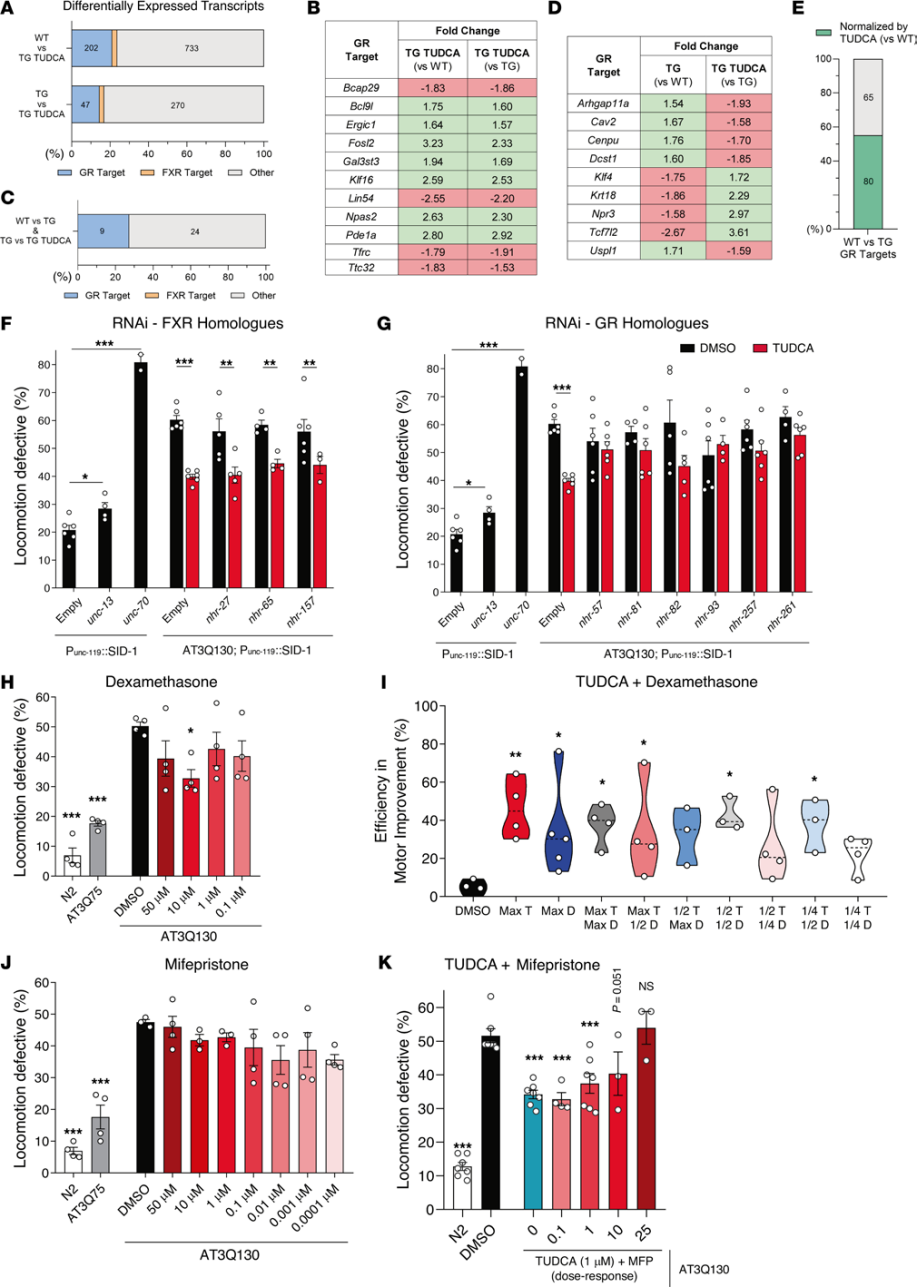
The effect of TUDCA is FXR-independent and GR-dependent. (**A**) Number of differentially expressed protein-coding genes when comparing WT and TUDCA mice, and TG and TG TUDCA mice, in the RNA-Seq analysis. The total number of genes corresponds to 100% (xx axis), and the absolute number of genes is indicated inside each bar. The number of differentially expressed FXR targets is 25 and 8 in the WT versus TG TUDCA and TG versus TG TUDCA comparisons, respectively. (**B**) Fold change of GR target genes that are simultaneously differentially expressed in WT versus TG TUDCA and TG versus TG TUDCA comparisons. (**C**) Number of genes that are simultaneously differentially expressed in WT versus TG and TG versus TG TUDCA comparisons. No FXR target genes were observed. (**D**) Fold change of GR target genes that are simultaneously differentially expressed in WT versus TG and TG versus TG TUDCA comparisons. (**E**) GR target genes that are differentially expressed in the WT versus TG comparison, but not in the WT versus TG TUDCA comparison. (**F**) Evaluation of the effect of TUDCA treatment in AT3Q130 animals crossed with the RNAi-sensitive strain LC108 upon simultaneous silencing of FXR orthologue genes and treatment with TUDCA. The silencing of all 3 genes did not the change the positive effect of TUDCA. *unc-13* and *unc-70* were silenced as controls for RNAi efficiency. (**G**) The effect of TUDCA after silencing the GR orthologues showed that the compound’s effect was lost. (**H**) Dose-response assay for the effects of dexamethasone in the locomotion of AT3Q130 animals. (**I**) Percentage of improvement in locomotion defective animals (compared with N2 nematodes) of several combinations of both TUDCA (T) with dexamethasone (**D**). Max T, 1 μM; 1/2 T, 0.5 μM; 1/4 T, 0.25 μM; Max D, 10 μM; 1/2 D, 5 μM; 1/4 D, 2.5 μM. (**J**) Dose-response evaluation of the effect of mifepristone in reducing the percentage of locomotion-defective AT3Q130 animals. (**K**) Dose-response assay for the effects of TUDCA (1 μM) in combination with increasing concentrations of the GR antagonist mifepristone (MFP), in the locomotion of AT3Q130 animals. The 1% DMSO condition was used as the negative control for drug/vehicle. (**F**–**K**) 150–300 animals were assayed across 3–7 independent experiments. AT3Q75 animals were used as a TG non-motor-defective control, and 1% DMSO was used as the negative control drug. 1-Way ANOVA, * *P* < 0.05, ** *P* < 0.01, *** *P* < 0.001.

We performed a transcriptomic analysis by RNA sequencing of the brainstem of TUDCA-treated mice (Supplemental Figure 8A), followed by reverse-transcription quantitative PCR (RT-qPCR) validation of representative transcripts (Supplemental Figure 8B). We identified 25 and 8 FXR target genes as differentially expressed between WT and TG TUDCA, and TG and TG TUDCA mice, respectively (Figure 4A). However, a significantly higher number of GR transcripts was found to be differentially expressed, namely 202 and 47 between WT and TG TUDCA, and TG and TG TUDCA mice, respectively (Figure 4A). Remarkably, the expression of GR target genes that were common to both comparisons always varied in the same direction (up or downregulation) (Figure 4B), indicating a consistent effect for TUDCA. When we assessed genes that were differentially expressed between WT and TG, and TG and TG TUDCA mice simultaneously, we observed that about 30% were GR targets (Figure 4C) and that their expression changed in the opposite direction between groups (Figure 4D), indicating a recovery effect induced by TUDCA in TG mice. None of those genes were FXR targets. Finally, we assessed genes that were differentially expressed between WT and TG mice, but not between WT and TG TUDCA mice, and observed that approximately 55% of them were GR targets (Figure 4E). In addition, formerly described GR targets based on transcriptomic analyses of other neuronal areas (51, 52) showed unchanged expression between WT, TG, and TG TUDCA mice (Supplemental Figure 8C), which was nevertheless consistent with our transcriptomic data. This unbiased approach strongly suggests that TUDCA modulates the expression of GR targets in the brainstem, and consistently recovers expression changes of these genes in the disease context, without major changes in FXR-dependent transcripts.

Next, we wanted to functionally assess whether the GR was in fact required for the effect of TUDCA in vivo. For this, we used a gene silencing approach in the AT3Q130 nematode model. To ensure that candidate targets were being silenced in neurons, we crossed SCA3 TG worms with a *C*. *elegans* strain with increased sensitivity to RNAi in neurons, before feeding animals with dsRNA-expressing bacteria (53). Firstly, we observed that singularly silencing 3 FXR orthologues (Supplemental Table 4) did not change the positive effect of TUDCA on the motor phenotype (Figure 4F). Afterward, we looked at GR orthologues in worms (Supplemental Table 4) and observed that the positive effect of TUDCA was fully lost upon silencing of those GR orthologues (Figure 4G). We attributed this effect to most NHRs due to their high sequence similarity, and that each dsRNA clone is probably targeting several of these genes simultaneously.

To further evaluate a possible GR-dependent mechanism, we treated AT3Q130 animals with dexamethasone, a well-known GR agonist, at concentrations defined to be nontoxic to WT animals (Supplemental Figure 8D). We observed a positive effect of this drug on improving locomotion of TG worms (Figure 4H), suggesting that GR activation is beneficial for diseased animals. Next, to evaluate if their effect is mediated by a common pathway or not, we cotreated animals with both dexamethasone and TUDCA. We observed that the efficiency in locomotion improvement was similar between TUDCA-only, dexamethasone-only and cotreated animals (Figure 4I), with no additive or synergistic effects, strongly suggesting a common pathway of action for these drugs.

Finally, to assess if TUDCA was dependent on a functionally active GR, we cotreated AT3Q130 nematodes with TUDCA and the GR antagonist mifepristone. After defining nontoxic concentrations of mifepristone on WT animals (Supplemental Figure 8E) as well as confirming no effect of this molecule on the locomotion of WT and TG animals (Supplemental Figure 8F and Figure 4J, respectively), we observed a dose-dependent loss of TUDCA’s effect upon GR antagonism (Figure 4K).

**Figure 5:**
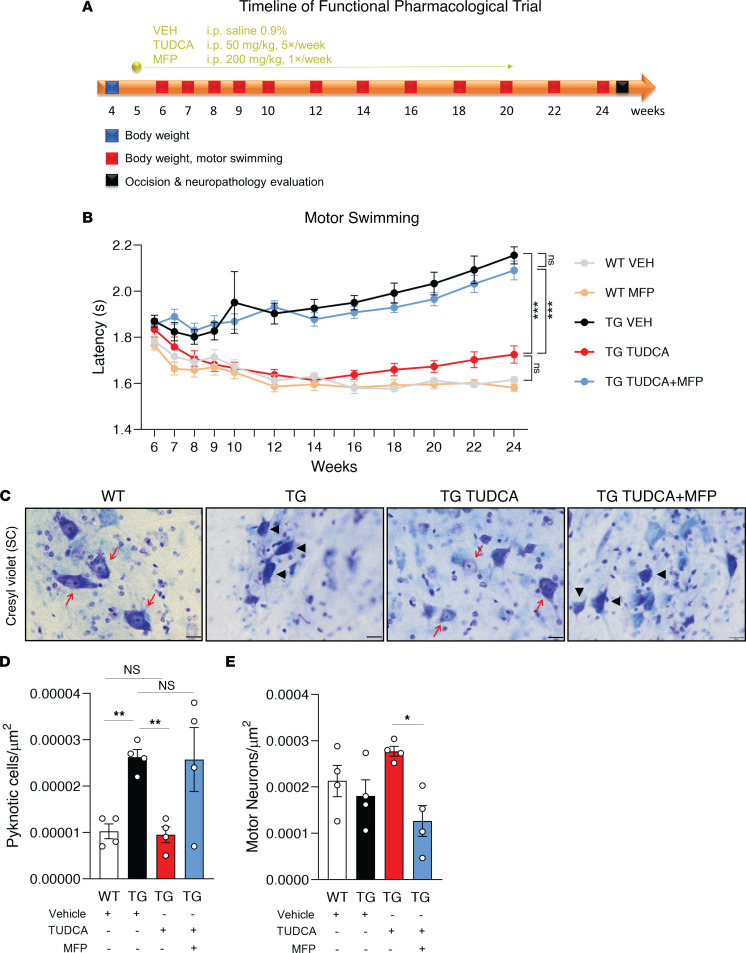
The positive effect of TUDCA in motor and neuropathological phenotypes of SCA3 mice is fully GR-dependent. (**A**) Schematic representation of the timeline of the preclinical trial with TUDCA and mifepristone cotreatment. Colored squares indicate performed tests at the indicated timepoints. (**B**) Assessment of the time taken for a mouse to swim through a 60 cm water path. TUDCA fully rescued the motor defects of SCA3 mice and its effect was fully abolished when coadministered with the GR antagonist mifepristone. A total of 11–15 mice per condition were assessed continuously in each time point. (**C**) Pyknotic cells and motor neurons per area were counted after cresyl violet staining in the spinal cord of treated 24-week-old mice, in a total of 4 animals per group. Black arrowheads show pyknotic cells, while red arrows show healthy cells. Scale bar: 20 μm. (**D**) Quantification of pyknotic cell and (**E**) motor neuron number per area. * *P* < 0.05, ** *P* < 0.01, *** *P* < 0.001. MFP, mifepristone; SC, spinal cord; TUDCA, tauroursodeoxycholic acid; VEH, vehicle. 1-Way ANOVA, * *P* < 0.05, ** *P* < 0.01, *** *P* < 0.001.

To lend further support to the therapeutic efficacy of TUDCA via the GR, we cotreated SCA3 mice with TUDCA and mifepristone, and evaluated their motor performance throughout disease progression (Figure 5A). TUDCA and mifepristone were administered parenterally with no detrimental effects observed (Supplemental Figure 8G), and the therapeutic effect of TUDCA (Figure 5B) was even more pronounced when compared to oral administration (Figure 1, D and E), reinforcing the therapeutic effect of this BA. Of utmost importance, the cotreatment of SCA3 mice with TUDCA and mifepristone, totally abolished the effect of TUDCA on mouse swimming performance, making these animals indistinguishable from the SCA3-vehicle mice (Figure 5B). The mifepristone cotreatment also reverted the positive effects of TUDCA on the neuropathology of SCA3 mice, increasing the number of pyknotic cells to SCA3 TG levels (Figure 5, C and D), with animals showing a decrease in the number of motor neurons (Figure 5, C and E).

These results further suggest that TUDCA administration is improving the phenotype of SCA3 nematodes and mice through a mechanism that is dependent on a functional GR and independent from the canonical FXR-mediated pathway, establishing a novel in vivo mechanism of action for BAs in neurons.

### GR dysfunction is a TUDCA-targetable conserved disease mechanism in SCA3.

**Figure 6:**
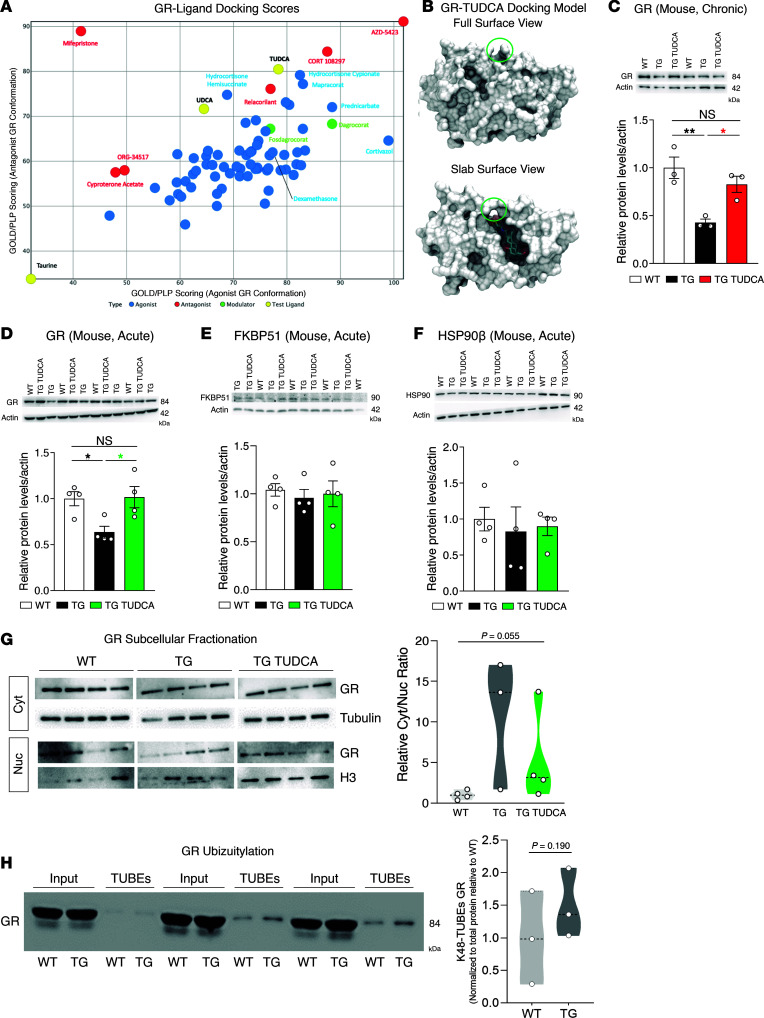
TUDCA promotes GR activity likely by preventing its degradation in CMVMJD135 mice. (**A**) GOLD/PLP scoring values of TUDCA, UDCA, taurine, and the known GR-binders to both GR conformation models, grouped into known agonist, antagonist and modulator models. (**B**) Representation of GR interaction with TUDCA in the agonist binding conformation. The left figure represents the full view of the surface of the protein, while the right figure represents a slab view, with a crosssection along the surface of the protein to show the accommodation of TUDCA inside the protein cavity. (**C**) Western blot analysis of GR levels, in the brainstem of 34 weeks-old mice. 3 animals per group were analyzed. (**D**) Western blot analysis of GR, (**E**) FKBP51 and (**F**) HSP90β protein levels in the brainstem of acutely treated mice. 4 animals per group were evaluated. Black *, WT versus TG; red *, TG versus chronic TG TUDCA; green *, TG versus acute TG TUDCA. (**G**) Western blot analysis of the GR in subcellular protein fractions, namely cytosolic (Cyt, normalized to tubulin) and nuclear (Nuc, normalized to H3). The relative cytosolic/nuclear ratio was determined by dividing the tubulin-normalized cytosolic GR levels by the H3-normalized nuclear GR levels. The molecular weight of each protein is: GR, 84 kDa; tubulin, 55 kDa; H3, 17 kDa. A total of 3-to-4 acutely treated mice were assessed. (**H**) Immunoblot for GR before (input) and after pulldown with tandem ubiquitin binding entities (TUBEs), in mixed protein extracts from the cerebellum and the brainstem. Three animals in 4 independent experiments were assessed and quantified. 1-Way ANOVA (**C**–**F**). Kruskal Wallis H test (**G**) and Student’s *t* test (**H**). * *P* < 0.05, ** *P* < 0.01.

After establishing the GR-dependency of the effect of TUDCA in the nematode and mouse models of SCA3, we set out to assess whether TUDCA is predicted to physically bind to the human GR. We generated a bioinformatic model to assess protein-ligand affinity between TUDCA and the human GR using GOLD/PLP scoring. Surprisingly, we observed a very strong score for TUDCA-GR binding, which was even higher than that for a well described BA receptor (the GPBAR) (Table 1). UDCA also showed strong predicted binding to the GR, albeit less than TUDCA, and taurine is unlikely to bind to the receptor (Table 1). It is also interesting to observe that the predicted GR-docking TUDCA affinity is very similar to that of classical GR ligands such as hydrocortisone (Figure 6A). While TUDCA is predicted to bind to both agonist (Figure 6B) and antagonist (Supplemental Figure 9A) conformations of GR, these findings are also observed for classically described GR agonists (Figure 6A) (54).

Next, we evaluated whether this mechanism was relevant for the SCA3 mouse model. It was surprising to us that GR protein levels were significantly decreased in the brainstem of TG mice and were restored upon treatment (Figure 6C); however, the levels remained unchanged in the hippocampus, which is a control brain region in this disease model (Supplemental Figure 9B). To dissect the immediate molecular response to the treatment, we administered TUDCA through i.p. injection in a second set of mice, with a treatment duration of 7 days (acute treatment) (Supplemental Figure 9C). In TG mice, the GR protein levels were also significantly decreased, and TUDCA fully restored them to WT levels (Figure 6D). No changes were observed for the GR chaperones FKBP51 (Figure 6E) and HSP90 (Figure 6F). Levels of serum corticosterone were also increased in TG mice, with no change upon treatment, which is in line with a normal long-term response of the hypothalamic-pituitary-adrenal axis to a decrease in GR function (Supplemental Figure 9D). No changes were observed in the expression of *GR (Nr3c1*) and *Fkbp5* mRNAs, but a significant increase in *Hsp90* expression in TG mice was detected, which was normalized by TUDCA (Supplemental Figure 9E). This is in agreement with the absence of a change in protein levels, as excess *Hsp90* mRNA is known to be untranslated at physiological temperatures (55).

**Figure 7:**
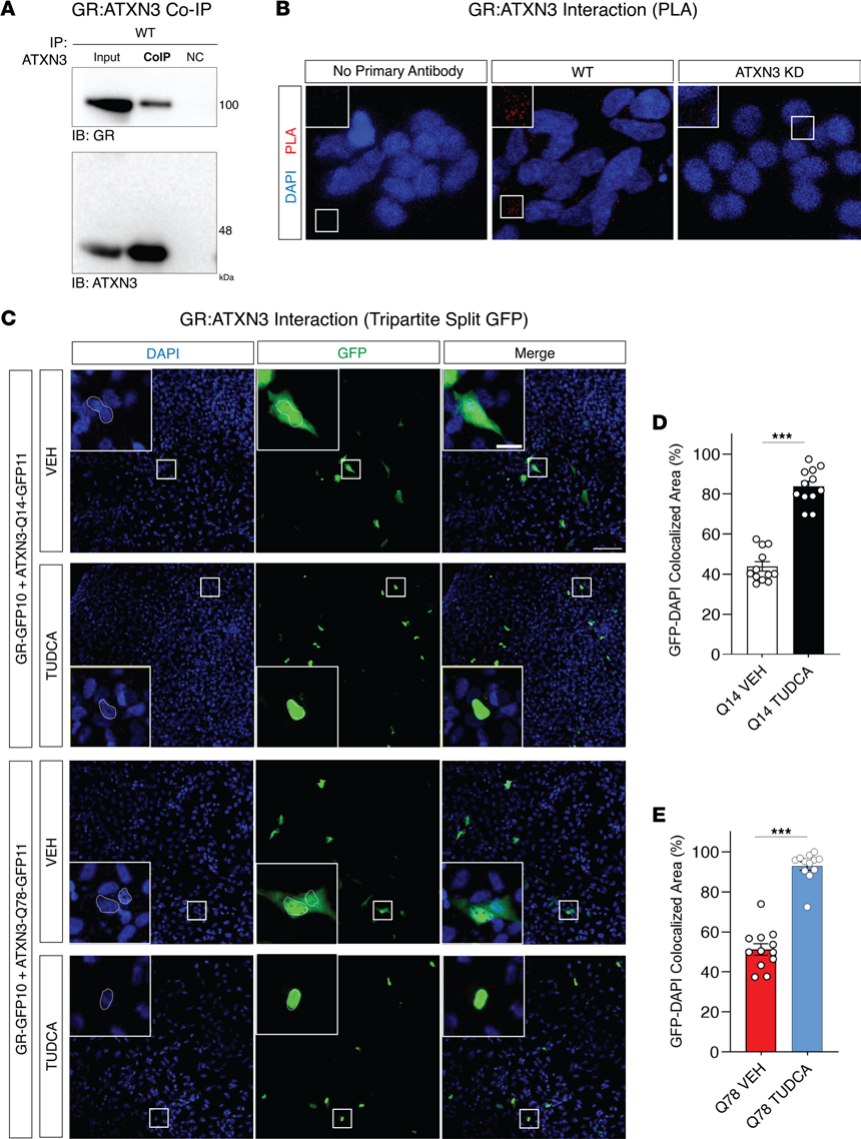
GR interacts with WT and mutant ATXN3, and is translocated to the nucleus upon TUDCA treatment. (**A**) Immunoprecipitation followed by Western blot showed coimmunoprecipitation of GR with ATXN3 (rabbit anti-ATXN3, MJD1-1) from mouse brain tissue lysates. IB, immunoblotting done with antibody against either GR or ATXN3 (mouse anti-ATXN3, 1H9). IP, immunoprecipitation done with an antibody against ATXN3. NC, isotype control antibody of the same isotype as the primary antibodies to discern specific binding from nonspecific interactions. A 40% increase in image contrast was applied from the original blots. (**B**) Representative fluorescence microphotographs of SH-SY5Y cells following DAPI staining and a PLA of GR and ATXN3, in both WT and cells with shRNA-mediated knockdown (KD) of ATXN3 expression. The negative control represents absence of primary antibodies, and ATXN3 KD cells show a scarcer signal compared with WT cells, as expected. Scale bars, 50 μm. (**C**) Tripartite split-GFP system fluorescence in MRC5-SV cells expressing GFP1-9. Cells were transfected with GR fused with GFP10 and ATXN3 fused with GFP11 (14Q or 78Q) and treated with and TUDCA at 100 μM for 24 hours. Upon protein interaction, GFP10 and GFP11 assemble, spontaneously associate with GFP1-9, and fluorescence is emitted. Green fluorescence at 488 nm excitation (GFP), DAPI nuclear staining (blue). Scale bars: 100 μm and 20 μm (inset). (**D**) Quantification of the percentage of the cellular area with colocalization of both GFP and DAPI signal in Q14 and (**E**) Q78 expressing cells with vehicle or TUDCA treatment. Mann-Whitney U test, *** *P* < 0.001.

Next, we explored the mechanism underlying the decrease in GR protein levels in mice bearing mutant ATXN3. Firstly, we observed an increase in the cytoplasmic fraction of GR in TG mice, which is partially countered by TUDCA (Figure 6G), suggesting that this compound may protect GR from ubiquitin-proteasome system–mediated (UPS-mediated) degradation, as it increases its nuclear translocation, a physiological process for this receptor upon ligand-binding. Moreover, we observed an increase in the proportion of ubiquitylated GR in TG mice (Figure 6H). This led us to hypothesize that ATXN3 might normally interact with the GR and function as a DUB toward this receptor, the mutant form of the protein being potentially less efficient in this action, thus causing an increased UPS-dependent GR degradation. Consistent with such a model, the endogenous GR coimmunoprecipitated with endogenous ataxin-3 in the mouse brain (Figure 7A). Using a proximity ligation assay (PLA), we observed that ATXN3 was physically close to GR (Figure 7B), colocalizing with this receptor at a distance smaller than 40 nm in human cells. The interaction between GR and ATXN3, as well as the predicted TUDCA-mediated nucleus translocation of the GR-ATXN3 complex, was also verified in a tripartite split GFP system (56), regardless of polyQ-length (Figure 7, C–E and Supplemental Figure 10). Taken together, these results lend support to the mutant form of the ATXN3 protein being able to bind the GR, like its WT counterpart, but hypothetically with a decreased GR DUB activity, causing its increased UPS-dependent degradation, and TUDCA acting, at least partially, by reducing availability of the GR for ubiquitylation/degradation in the cytoplasm.

### Human GR dysfunction is a potential therapeutic target in patients with SCA3.

**Figure 8:**
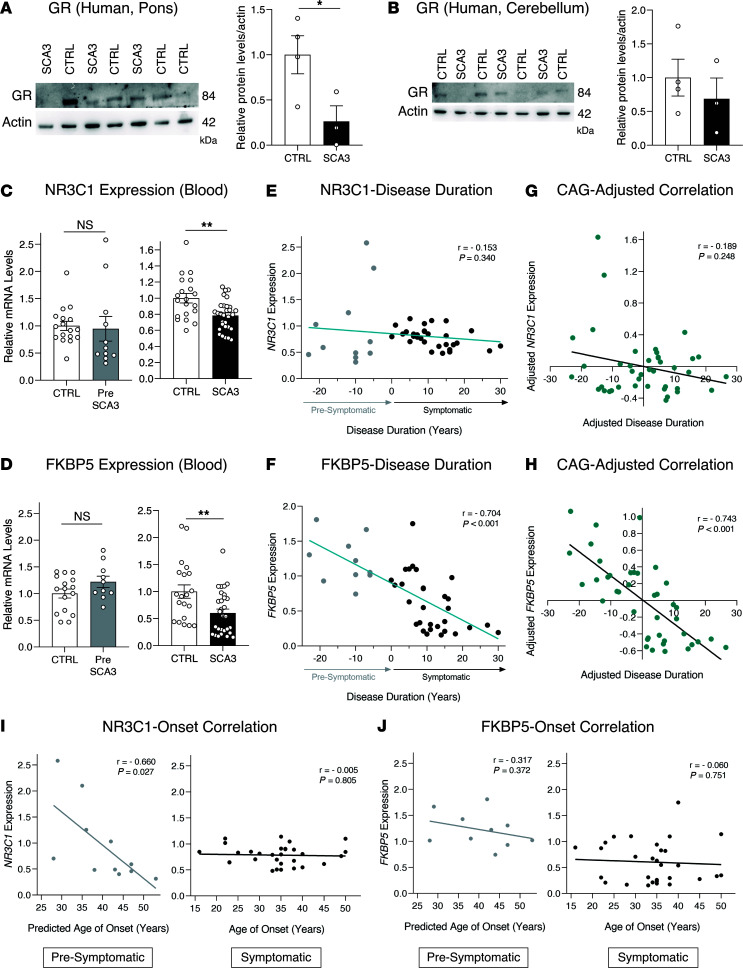
GR is a mechanistic target of TUDCA. (**A**) Western blot analysis of the GR, normalized for actin, in the pons and (**B**) cerebellum of patients with SCA3. A total of 4 control and 3 patients with SCA3 were evaluated. (**C**) RT-qPCR analysis of the expression levels of *GR* (*NR3C1*) or (**D**) *FKBP5* in the blood of patients with presymptomatic SCA3 (PreSCA3) and symptomatic SCA3 (SCA3), when compared the respective control groups. (**E**) Pearson’s correlation between the predicted time to disease onset (in patients who are presymptomatic) or disease duration (in patients who are symptomatic) with peripheral *GR* or (**F**) *FKBP5* expression. (**G**) Partial correlation between the predicted time to disease onset (in patients who are presymptomatic) or disease duration (in patients who are symptomatic) with peripheral *GR* or (**H**) *FBKP5* expression, when adjusting for the number of CAG repeats. (**I**) Pearson’s correlation between the predicted age of onset (in patients who are presymptomatic) or age of onset (in patients who are symptomatic) with peripheral *GR* or (**J**) *FKBP5* expression. A total of 11 patients withPreSCA3 (with 17 CTRL) and 30 patients with SCA3 (with 20 CTRL) were assessed. Student’s *t* test or Mann-Whitney U test for (**A**–**D**), Pearson Correlation Coefficient (r) was applied to (**E**–**J**). * *P* < 0.05, ** *P* < 0.01.

Despite establishing TUDCA as a predicted GR-ligand, it was still unknown whether GR dysfunction was also present in patients with SCA3. Protein levels of GR were decreased in the pons of patients with SCA3 (Figure 8A), with no change in the cerebellum (Figure 8B).

To assess the possible impact of GR dysfunction at the peripheral gene expression level, we collected blood samples from a cohort of 11 presymptomatic (PreSCA3) and 30 symptomatic patients with SCA3 (SCA3), each with their respective control (CTRL) group (Tables 2 and 3). Interestingly, we observed that the expression levels of *GR* (*NR3C1*) and *FKBP5* were unchanged in individuals in the PreSCA3 group, but significantly decreased in patients with SCA3 (Figure 8, C and D). Then, we determined the predicted time-to-onset in the PreSCA3 group (based on the number of CAG repeats) and, in combination with the disease duration in the SCA3 group, plotted it against results on gene expression. We observed that *GR* expression did not correlate with disease duration (Figure 8E). However, *FKBP5* expression negatively correlated with disease duration (Figure 8F), showing a steep decrease in expression throughout progression in both presymptomatic and symptomatic stages. Both correlations were unchanged upon adjustment for CAG number (Figure 8, G and H), sex (Supplemental Figure 11, A and B), and age (Supplemental Figure 11, C and D). This indicates that *FKBP5* is a potential indicator of SCA3 progression before and after clinical conversion.

Lastly, we analyzed the correlation between gene expression and the age of onset of disease (predicted age of onset for the PreSCA3 group). We observed that *GR* expression negatively correlated with the predicted onset in patients in the presymptomatic group, but not in the symptomatic group (Figure 8I). No significant correlations were observed for *FKBP5* expression (Figure 8J).

Interestingly, there was a positive correlation between *GR* and *FKBP5* expression in the PreSCA3 group (Supplemental Figure 11E) that was lost in the symptomatic patients with SCA3 (Supplemental Figure 11F). Altogether, these results indicate that GR signaling components show perturbed expression in SCA3 also at the peripheral level and that, in addition to being of therapeutic relevance, this pathway may be a source of potential peripheral biomarkers of disease to be studied in the future, namely for their capacity to predict the age at clinical conversion (*GR*), and to measure clinical progression (*FKBP5*).

## Discussion

The treatment of SCA3 is currently based on approaches that are not disease specific (33) and only partially effective, such as physical and speech therapy (57), dopaminergic agents for extrapyramidal symptoms (58) or botulinum toxin for dystonia (59). Currently, no single therapeutic agent can improve all symptoms and/or halt disease progression (34). Here, we showed that TUDCA was able to strongly improve abnormal behavioral phenotypes of 2 SCA3 animal models, that the effect of this compound was disease-specific, and that molecular and pathological features of the disease were also significantly ameliorated. Moreover, we established, for the first time, an in vivo mechanism of neuronal GR-dependency for the neuroprotective effect of TUDCA and proposed GR dysfunction as a source of candidate biomarkers of disease in patients with SCA3.

The most efficient BA molecule for improving the phenotype of AT3Q130 nematodes was TUDCA. While, according to our analysis, there was no evident correlation between efficiency and structure of each BA (for example regarding conjugated groups), it is also unlikely that the improved effect of TUDCA was due to its catabolism into taurine, since *C*. *elegans* does not express BA hydrolases (60, 61), nor was the bacteria used to feed the nematodes metabolically active (27). When it comes to treatment in the mouse model, it was striking that TUDCA improved both early and late-onset motor phenotypes. In association with the overall recovery of motor neuron death and pyknosis in several areas throughout the CNS, as well as with the absence of effects in WT animals, these results suggest that TUDCA is targeting overall disease-specific changes and not improving the general performance of animals independently of SCA3 pathophysiology.

Neuroinflammatory dyshomeostasis is consistently observed in the brain of CMVMJD135 mice at late disease stages and was detected postmortem in patients with SCA3 (48, 62). Because TUDCA and corticosteroids have well-established antiinflammatory and immunomodulatory properties (63–66), one hypothesis is that TUDCA is improving motor behavior by reestablishing neuroinflammatory homeostasis. However, this is unlikely to be the sole mechanism of action, since the onset of the motor phenotype in CMVMJD135 mice occurs much earlier than neuroinflammation is detected. Furthermore, the immunomodulatory effect of GR is dependent, both directly and indirectly, on the NF-κB pathway (67, 68), which we show to be unaffected by TUDCA treatment. Finally, it has been determined that the efficacy of corticosteroids in the management of immunological disorders of the CNS is much more dependent on modulation of the peripheral immune response than of the resident immune cells (69). While TUDCA might be protective through a direct antiinflammatory mechanism at later stages, it is much more likely that, in the case of SCA3, other neuron-directed effects underlie its beneficial impact from early disease stages, potentially since disease onset.

While it has been shown that mutant ATXN3 does not lose its DUB activity in vitro (13, 70, 71), the in vivo context is much more complex: the aggregation process can reduce the physical accessibility of ATXN3 to ubiquitin links and, therefore, compromise its DUB activity without loss of its intrinsic function (17). Indeed, our results show that the GR interacts with ATXN3, regardless of polyQ length, and that its ubiquitylation is likely increased in TG mice, suggesting an abnormal interaction and/or DUB activity of mutant ATXN3 toward this specific substrate. A less strong interaction and/or the loss of the GR ubiquitin chain editing mechanism could increase the signaling for its UPS-mediated degradation, culminating in the decrease of protein levels. From a pharmacological perspective, it is unsurprising that TUDCA can bind to the GR since both BAs and corticosteroids are formed from cholesterol (72). However, it was surprising that the predicted binding strength of TUDCA to the human GR surpassed that of several classical GR ligands. Therefore, considering the mechanism of action of NHRs, and that the effect of TUDCA in an in vivo model is dependent on a functionally active GR, we hypothesize that TUDCA binds to the GR, induces its nuclear translocation, and, consequently, prevents UPS-mediated degradation in the cytoplasm.

This hypothesis is also supported by the fact that TUDCA was able to modulate the expression of several GR target genes, which is the main outcome of receptor function (73). Indeed, more than half of the GR target genes whose expression was changed in SCA3 mice were restored upon TUDCA treatment. This indicates that TUDCA allows for recovery of the levels and subcellular localization of the GR protein, which, in turn, maintains its intrinsic activity. This restoration of transcriptional GR activity potentially underlies the beneficial effect of TUDCA seen in SCA3 mice. However, the GR transcriptional response is very diverse and tissue dependent (51, 52). In fact, none of the GR targets described in other CNS regions were altered in the brainstem, indicating that different GR target genes might be more or less relevant according to the tissue. As GR transcriptional targets are still unknown for the most affected CNS areas in SCA3, an unbiased chromatin immunoprecipitation–Seq (ChIP-Seq) of GR-bound targets in the brainstem is likely required to address this. Nevertheless, it is noteworthy that a GR-dependent immunomodulatory pathway for UDCA has been described in liver cells (65) and that GDCA is able to increase GR activity in rat hypothalamic neurons (74), supporting our hypothesis of a relevant GR-dependent neurosteroid mode of action for TUDCA. While it is worthwhile mentioning that the efficacy of TUDCA could also be mediated by the progesterone receptor (PR), as mifepristone is an inhibitor of PR (75), our data regarding the genetic knockdown of the GR orthologue in nematodes and the overall proof of GR dysfunction in SCA3 makes this hypothesis less likely.

The translation of TUDCA to patients is promising, yields several advantages, and is easier than introduction of a novel drug (66). A strong improvement in motor function was observed with per os administration, and the compound is safe and has little long-term side effects (76, 77). This is even more important in the context of SCA3, since chronic treatment is possible even prior to disease onset, due to the possibility of presymptomatic testing (78). The repurposing of this drug, with positive results in patients, would be a more immediate strategy for the management of SCA3 than gene therapy approaches, which it does not, nevertheless, exclude.

One of the key requirements for a robust clinical trial design is the existence of a disease-relevant biomarker. In this perspective, it is of interest that the peripheral expression of *GR* and *FKBP5* is decreased in patients with SCA3 who already show symptoms, but not before clinical conversion. Nevertheless, each gene shows a very different profile throughout asymptomatic and symptomatic disease progression. Expression of *GR* is increased in presymptomatic patients with a predicted earlier onset but is independent of the age of onset in patients after clinical conversion. This suggests that increased *GR* expression is a marker for patients that will develop disease sooner and may track the time of conversion when it begins to decrease. Notwithstanding, longitudinal studies are needed to thoroughly assess this hypothesis. While *GR* expression seems to be a potential predictor of onset/conversion, *FKBP5* shows a profile that is independent of onset, but continuously decreases throughout the natural history of disease, even in the presymptomatic stage. This indicates it as a candidate biomarker of progression, which, if validated, might be used to assess the efficacy of progression-attenuating drugs in future clinical trials. Mechanistically, we can hypothesize that these results reflect increased GR protein degradation; on one hand, the *FKBP5* expression decrease is directly related to progressive GR decrease as the disorders aggravates; on the other hand, increased *GR* expression only in the preclinical state may represent a transcriptional overcompensation to an initial state of GR protein decrease, as it is well described for other endocrine dysfunctions (79). In summary, we propose GR dysfunction as a pathophysiological mechanism in SCA3 that can simultaneously provide candidate biomarkers of disease and a therapeutic target, namely TUDCA, which can be repurposed as a safe and effective disease-modifying drug for this disorder.

## Methods

### C. elegans strains and general methods

Standard methods were used for culturing and observing *C*. *elegans* (80). Briefly, nematodes were grown on nematode growth medium (NGM) plates seeded with *Escherichia coli* OP50 strain at 20°C. All assays were performed at 20°C on 4-day-old animals synchronized by 20% alkaline hypochlorite treatment. Strains used in this work were: Bristol N2, AM599 [*rmIs261* [P_F25B3.3_:AT3v1-1Q75:YFP]], AM685 [*rmIs263* [P_F25B3.3_:AT3v1-1Q130:YFP]], CB1370 [*daf-2(e1370)* III], CF1038 [*daf-16(mu86)* I], LC108 [*uIs69* [*pCFJ90(myo-2p:mCherry) + unc-119p:sid-1*]], and MAC161 [*uIs69* [*pCFJ90(myo-2p:mCherry) + unc-119p:sid-1*]; *rmIs263* [P_F25B3.3_:AT3v1-1Q130:YFP]].

### C. elegans drug treatment and compound preparation

All compounds were prepared at 100 × working concentration in DMSO, so that all treatments were performed at 1% DMSO (such as controls without drug). TUDCA and UDCA were obtained from Sigma-Aldrich, while all other compounds were obtained as part of the SCREEN-WELL Nuclear Receptor ligand library from Enzo Life Sciences.

### C. elegans behavioral evaluation

Toxicity, motility, and lifespan assays were performed as previously described (27). Detailed information can be found in supplemental methods.

### C. elegans RNAi-mediated gene silencing

RNAi feeding of HT115 bacteria expressing dsRNA cDNA from the desired target genes was used as a gene silencing approach. All bacterial clones were obtained from the Ahringer RNAi library (53). NGM plates were supplemented with both ampicillin (100 μg/mL) and isopropyl β-D-1-thiogalactopyranoside (400 μM). These were seeded 24 hours after pouring with the respective bacterial clone (grown to an OD_595_ of 1.500 in LB with 100 μg/mL ampicillin), with or without 1 μM TUDCA in 1% DMSO, and dried at room temperature for 48 hours. Animals were then synchronized by allowing 10 adult animals to lay eggs for 3 hours and evaluated 96 hours later using the motility assay described above.

### Mouse strains and general methods

Female and male CMVMJD135 (C57BL/6J background) mice were used in the study. TG and non-TG drug- and vehicle- treated animals were alternately assigned and housed at weaning in groups of 5 animals in filter-topped polysulfone cages 267 × 207 × 140 mm (370 cm^2^ floor area) (Tecniplast), with corncob bedding (Scobis Due, Mucedola SRL) in a conventional animal facility. Progeny resulting from mating SCA3 TG and WT animals was genotyped at weaning by PCR, as previously described (48).

Housing and health monitoring details can be found in supplemental methods. At the end of behavioral evaluation, at week 34, the animals were humanely euthanized either by decapitation or exsanguination perfusion with saline or PFA 4% under deep anesthesia with a mixture of ketamine hydrochloride (150 mg/kg) plus medetomidine (0.3 mg/kg). Brain, spinal cord, and blood (to measure TUDCA serum levels, detailed in supplemental methods) were collected.

### Mouse treatment with TUDCA and mifepristone

For the chronic and acute treatments, CMVMJD135 and WT male and female mice were used. TUDCA (Sigma-Aldrich) was administered in the standard diet at 0.4% or injected intraperitoneally at 50 mg/Kg as previously described (42, 43). Mifepristone (MFP, Sigma-Aldrich) was injected i.p. at 200 mg/Kg. Detailed information on drug regimen can be found in supplemental methods.

### Mouse phenotype analysis and neuropathology assessment

Mouse behavioral assessment was performed as previously described (27, 48) and included body weight, beam and motor swimming tests, vertical and horizontal exploratory spontaneous activity, footprint analysis, and hanging wire grid test. Detailed information on motor behavior tests and neuropathology/IHC, as well as qRT-PCR and Western blot can be found in supplemental methods.

### Tridimensional astrocyte morphology analysis

Astrocytic morphology of spinal cord and pontine nuclei was assessed with the Simple Neurite Tracer plugin of ImageJ (81). Briefly, 40 μm free-floating sagittal sections of mouse brains were collected and immunofluorescence for GFAP was performed using standard techniques. 4 animals per group were used and 3–5 representative sections were examined in an Olympus FV1000 confocal microscope, followed by tridimensional reconstruction of astrocytes.

### RT-qPCR analysis

Total RNA from mouse tissue was extracted using TRIzol (Invitrogen), according to the manufacturer’s instructions. A brief description can be found in supplemental methods. All oligonucleotide sequences are available in Supplemental Table 7. For data analysis, the 2^–ΔΔCt^ method was used. The gene expression levels were normalized for both the housekeeping and control groups.

### RNA-Seq

RNA was extracted from flash-frozen brainstem of 34-week-old mice using the AllPrep DNA/RNA/miRNA kit (Qiagen), following the manufacturer’s instructions. A total of 3 mice per group (WT, TG, and 7 day TUDCA–treated TG mice) was evaluated. Library preparation was carried out by targeted amplicon amplification for AmpliSeq (82). The quality of assembled libraries was assessed using the Agilent Bioanalyzer High Sensitivity chip. Library amplification was performed by emulsion PCR in an Ion Chef Instrument (Thermo Fisher Scientific), and cDNA quality was assessed with the Agilent TapeStation High Sensitivity tape. Sequencing was performed in the Ion S5 XL sequencer (Thermo Fisher Scientific), and reads were aligned to the AmpliSeq Mouse Transcriptome v1, obtaining a total of 23,930 transcripts. Results analyses are described in supplemental methods.

### Ubiquitylation assay

Tissue from the brainstem, cerebellum, and cervical spinal cord was mixed and homogenized in lysis buffer (0.15 M NaCl, 50 mM HEPES pH 7.5, 1 mM EDTA, 10% glycerol, 1% NP-40, 50 μ*M* PR-619) and complete protease inhibitor (Roche). Protein extracts were centrifuged at 4°C and 14,000*g* for 25 minutes. A total of 2 mg of the supernatant was incubated with 1:50 of K48 agarose-tandem ubiquitin binding entities (TUBEs, LifeSensors) at 4°C overnight with agitation. In the following day, 50 μL of magnetic agarose beads (DynaBeads, Invitrogen) were washed with lysis buffer using a magnetic particle concentrator. The protein lysate bound to the K48-TUBEs were incubated with these beads for 6 hours at 4°C in the orbital rotator. The bound beads were washed 5 times using lysis buffer, followed by elution of bound proteins in Laemmli buffer (containing 0.1% SDS). Protein samples were used for Western blot and probed with GR antibody, as previously described; 10% of the original cell lysate was used as input.

### Subcellular fractionation of mouse tissue

Fractionation of mouse tissue in cytosolic and nuclear fractions was performed as previously described (83). Both cytosolic and nuclear fractions were used for Western blot analysis, as described above. Efficiency of the fractionation was confirmed by the absence of H3 and α-tubulin in the cytosolic and nuclear fractions, respectively, and presence in the nuclear and cytosolic counterparts. For more information see supplemental methods section.

### Cell culture and differentiation

SH-SY5Y human neuroblastoma cells (ATCC CRL-2266TM) were maintained in DMEM/F-12 (Invitrogen) supplemented with 10% FBS (Biochrom), 2 mM GlutaMAX (Invitrogen), 100 U/mL penicillin, 100 μg/mL streptomycin, and 25 ng/mL puromycin (Sigma-Aldrich). Cells were differentiated by treatment with 0.1 μM all-trans retinoic acid (Sigma-Aldrich) in Opti-MEM (Invitrogen) supplemented with 0.5% FBS. WT and ATXN3-KD cells (expressing an shRNA that targets ATXN3) were used (84).

### PLA and confocal microscopy

The Duolink PLA Technology kit (Sigma-Aldrich) was used to assess GR-ATXN3 interaction. SH-SY5Y cells were fixed with 4% paraformaldehyde (PFA), permeabilized with 0.05% saponin and blocked with 1% BSA. Sequential incubation with mouse anti-ATXN3 clone 1H9 (1:100, Sigma-Aldrich, MAB5360) and rabbit anti-GR (1:500, Santa Cruz Biotechnology, sc-393232) was carried out. Afterward, cells were incubated with Duolink In Situ PLA Probe Anti-Mouse PLUS and Duolink In Situ PLA Probe Anti-Rabbit MINUS. The signal was detected with the Duolink In Situ Detection Reagents Red and cells were mounted with Duolink In Situ Mounting Medium with DAPI. Imaging was performed in an Olympus FV1000 confocal microscope. The laser excitation lines at 405 (DAPI) and 594 (TexasRed) were used, with the pinhole set at 1.0 Airy units. Image acquisition was performed using a 60 × oil objective at a resolution of 1024 × 1024 pixels and z-stacking. ImageJ software was used for image processing.

### Coimmunoprecipitation

The cerebellum and brainstem from WT mice were homogenized through mechanical disruption with lysis buffer (50 mM Tris-HCl, 150 mM NaCl, 1 mM EDTA, 10% Glycerol, 1% NP-40, 1% PMSF, and 1 × Protease inhibitors). The homogenates were then centrifuged at 9,500*g* for 25 minutes. The supernatant was collected, and the protein concentration was determined using a BCA Protein Assay Kit (Thermo Fisher Scientific). Magnetic beads from the Dynabeads Antibody Coupling Kit (Thermo Fisher Scientific) were coupled with anti-ATXN3 (rabbit anti-MJD; ref. 85) and rabbit IgG isotype control (Thermo Fisher Scientific, 10500C), following the manufacturer’s instructions. Dynabeads coupled with anti-ATXN3 and control IgGs were incubated with 2 mg of brain lysates at 4°C with rotation for 16 hours. After incubation, the flow through was recovered, and the Dynabead complexes were washed three times with ice-cold lysis buffer. Finally, protein complexes were eluted from Dynabeads with SDS-page buffer for 15 minutes at 95°C and visualized by Western blot.

### Tripartite split GFP association assay

#### Cloning for the tripartite split GFP system.

Constructs with GFP10 (pcDNA_GFP10-Nter) and GFP11 (pcDNA_GFP11-Cter) were modified to introduce our genes of interest. PCR information is presented in supplemental methods.

#### Cell culture, transfection and drug treatment.

MRC5-SV (immortalized normal pulmonary human fibroblasts) cells expressing GFP1–9 and a single-domain antibody, based on camelid heavy-chain antibodies (VHH or nanobody), engineered to boost GFP fluorescence by modulating the spectral properties of WT GFP were used. Transfection of plasmids was performed using Fugene (FUGENE HD Transfection Reagent, Promega) according to the manufacturer’s instructions. For transient expression of interacting proteins, 0.5 × 10^6^ MRC5-SV1–9 cells per mL were seeded in 12-well plates with sterilized coated coverslips and transfected at a ratio 1:1 of the GFP10 and GFP11 fusion vectors (1 μg). In the same day of the transfection, after 6 hours of cell recovery, they were treated with TUDCA (T0266 Sigma-Aldrich) at 25, 50, or 100 μM for 24 hours. The treatment with Dexamethasone (DEX; Fortecortin, Merck) was performed at a final concentration of 10^−6^ M for 24 hours as previously described (86). Each condition was repeated at least 3 times. Cell fixation, staining, and microscopy analysis are described in the supplemental methods section.

### Molecular modelling for protein-ligand docking

Structures for human GR were obtained from the Protein Data Bank (PDB) (87), and structures 3H52 and 6EL9 were selected to model GR. 3H52 represents the crystal structure of the human GR in an antagonist conformation (88), complexed with the antagonist molecule mifepristone, with a resolution of 2.80 Å. 6EL9 corresponds to the crystallographic structure of the human GR in an agonist-bound conformation (89), complexed with the agonist molecule AZD9567, with a resolution of 2.19 Å. In addition, a structure of the G-protein-coupled BA receptor (GPBAR), was also considered for comparison purposes, namely structure 7CFN (90), corresponding to the cryo-EM structure of GPBAR complexed with the BA derivative INT-777, with a resolution of 3.0 Å. More information can be found in supplemental methods and Supplemental Table 5.

### Human samples

Human postmortem brain samples were obtained from the Michigan Brain Bank (University of Michigan, Ann Arbor, Michigan, USA). Blood samples were collected from 11 patients qualifying as PreSCA3 (with 17 CTRL) and 30 patients with SCA3 (with 20 CTRL). All patients with SCA3 had prior clinical and molecular diagnosis of the disease and details on human sample procedures can be found in supplemental methods. Clinical, demographic and pathology information of each patient is summarized in Supplemental Table 6.

### Statistics

A CI of 95% was assumed for all statistical tests. Several statistical assumptions were considered, and the appropriate statistical test was applied according to the variable, number of groups, multiple comparisons, and presence of outliers. Regarding continuous variables, the assumption of normality was assessed by qualitative analysis of Q-Q plots and frequency distributions, the *z*-score of skewness and kurtosis, as well as by the Kolmogorov-Smirnov and Shapiro-Wilk tests. The assumption of homogeneity of variances was tested by Levene’s test and assumed for all variables. For repeated measurements, sphericity was tested using Mauchly’s test, and assumed for all tested variables. Values that deviated more than 1.5 interquartile ranges from the mean were considered outliers and were excluded from further analyses. For the comparison of means between 2 groups, the 2-tailed unpaired Student’s *t* test or the Mann-Whitney U test was used when data were normally or nonnormally distributed, respectively. For the comparison of mean locomotion impairment in *C*. *elegans* experiments, a 1-way analysis of variance (ANOVA) was used, followed by Dunnett’s post hoc test using AT3Q130 + DMSO as the control category. For the RNAi experiments, pairwise comparisons were performed using an orthogonal planned contrast analysis in the 1-way ANOVA model. All other mean comparisons with more than 2 groups were carried out using a 1-way ANOVA followed by Tukey’s HSD post hoc test, or a Kruskal-Wallis test when data were normally or nonnormally distributed, respectively. Regarding the comparison of means with one between-subjects and one within-subjects factor, a mixed design ANOVA model was used, followed by Tukey’s HSD post hoc test for between-subjects variables. For the comparison of medians of discrete variables across time points, a Friedman’s ANOVA was carried out, with pairwise comparisons through the Kruskal-Wallis statistic. For the comparison of categorical variables, Fisher’s exact test was used. *C*. *elegans* lifespan data was analyzed using a Cox regression model with the condition as a categorical covariable and a simple contrasts analysis. Correlations were carried out using Pearson’s correlation coefficient. Effect size measurements are reported for all analyses (Cohen’s *d* for *t* tests, *r* for nonparametric tests and correlation, ω^2^ or ω^2^_p_ for ANOVAs, φ for categorical analyses, and the hazard ratio for the survival analysis). TUDCA-treated WT mice were included in all mouse behavioral statistical analyses. All statistical tests were performed using SPSS 25.0 (SPSS Inc.) and are reported in Supplemental Table 1.

### Study approval

#### Animals.

Animal experimentation and all the procedures applied to the animals were in accordance with European (Directive 2010/63/EU revising Directive 86/609/EEC on the protection of animals used for scientific purposes) and National laws. Animal facilities and the people directly involved in vertebrate animal experiments were certified by the Portuguese regulatory entity (Direção Geral de Alimentação e Veterinária - DGAV). All performed protocols were approved by the Animal Ethics Committee of the Life and Health Sciences Research Institute (SECVS 120/2014), University of Minho and by the DGAV (reference 020317).

#### Humans.

Blood samples were collected from patients qualified as PreSCA and patients with SCA3, and respective controls recruited at the Department of Neurology of the Hospital Divino Espírito Santo/Universidade dos Açores (Ponta Delgada, Azores, Portugal), after approval of the study by the Ethics Committee. Participants provided informed consent as requested prior to blood collection.

### Data and materials availability

All data associated with this study are presented in the paper or in the Supplemental Materials. The data discussed in this publication have been deposited in NCBI’s Gene Expression Omnibus (91) and are accessible through GEO Series accession number GSE252250 (https://www.ncbi.nlm.nih.gov/geo/query/acc.cgi?acc=GSE252250). A Supporting data values list is also provided.

## Author contributions

SDS, JDDS, and PM conceptualized the project. SDS and JDDS were involved in formal analysis, methodology, validation, and visualization of the project and wrote the original draft of the manuscript. SDS, JDDS, MDC, ANC, MR, CSC, JSC, GNG, HSF, SO, DMF, LMC, CMPR, SFS, ARFF, FR, JPS, DVC, SG, SC, JC, and ATC were responsible for conducting the investigation. ML, ATC, and PM acquired funding, performed project administration tasks, acquired resources, and supervised the project. SDS, JDDS, ATC, and PM reviewed and edited the manuscript. Despite equal contribution for the final shape of the manuscript, SDS was involved in the genesis of the study and so was listed first in the order of cofirst authorship. Alphabetical order was used for the other 2 first authors.

## Supplementary Material

Supplemental data

Unedited blot and gel images

Supplemental table 5

Supplemental table 6

Supplemental table 7

Supplemental video 1

Supplemental video 2

Supporting data values

## Figures and Tables

**Table 3:**
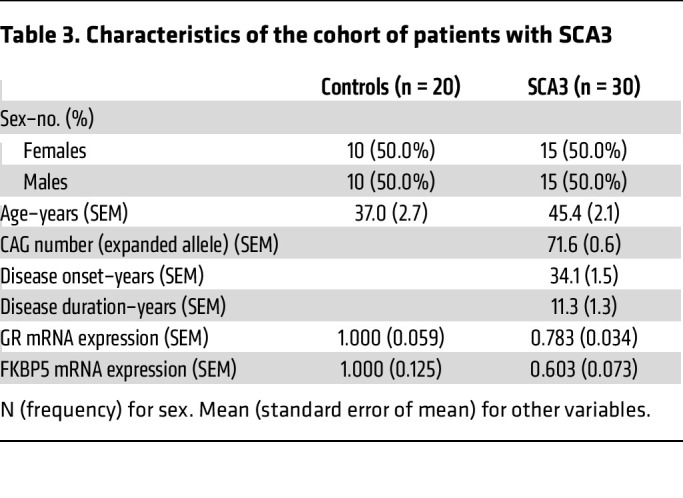
Characteristics of the cohort of patients with SCA3

**Table 2:**
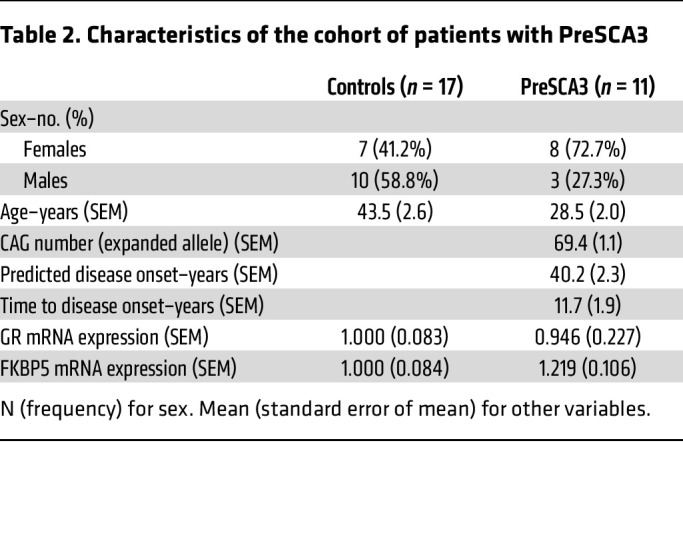
Characteristics of the cohort of patients with PreSCA3

**Table 1:**
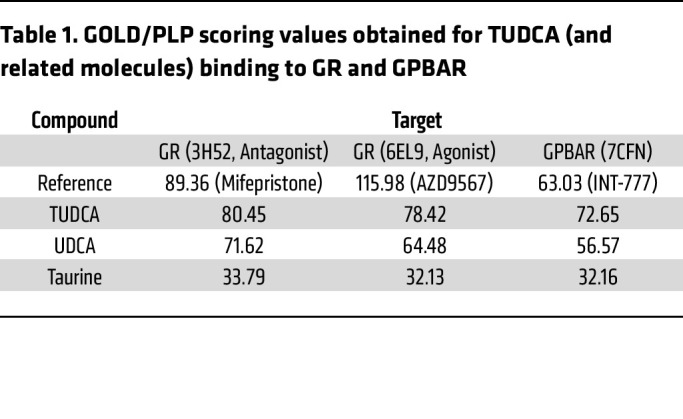
GOLD/PLP scoring values obtained for TUDCA (and related molecules) binding to GR and GPBAR

## Version 1 01/16/2024

In-Press Preview

## Version 2 03/01/2024

Electronic publication
