# Supplementary material for: Glucocorticoid receptor-dependent therapeutic efficacy of tauroursodeoxycholic acid in preclinical models of spinocerebellar ataxia type 3
Source: J Clin Invest. 2024 Mar 1;134(5):e162246. doi: 10.1172/JCI162246 (PMC10904051; doi:10.1172/JCI162246)
Supplement: Supplemental data [file jci-134-162246-s141.pdf]

Supplementary Materials

Supplementary figures

Fig. S1. Toxicity assays of bile acids in N2 *C. elegans*. Drug toxicity was evaluated by measuring the optical density at 600 nm (OD_{600}) as a readout of OP50 consumption (food source), from day 0 (egg) to day 7. Treatments with 1% DMSO and 5% DMSO were used as non-toxic and toxic controls, respectively. Concentrations ranging from 0.0001 µM to 50 µM were tested for all compounds. (**A**) Chenodeoxycholic acid (CDCA). (**B**) Cholic acid (CA). (**C**) Deoxycholic acid (DCA). (**D**) Glycocholic acid (GCA). (**E**) Glycodeoxycholic acid (GDCA). (**F**) Taurocholic acid (TCA). (**G**) Taurodeoxycholic acid (TDCA). (**H**) Tauroursodeoxycholic acid (TDCA). (**I**) Ursodeoxycholic acid (UDCA).

Fig. S2. Impact of bile acids in the motor defects of AT3Q130 animals. Dose-response evaluation of the effect of selected bile acids in reducing the percentage of locomotion defective AT3Q130 animals. AT3Q75 animals were used as a transgenic non-defective control, and 1% DMSO was used as the negative control drug. A total of 3-4 independent experiments with 150-200 animals was performed per individual compound. (**A**) Chenodeoxycholic acid (CDCA). (**B**) Cholic acid (CA). (**C**) Deoxycholic acid (DCA). (**D**) Glycocholic acid (GCA). (**E**) Glycodeoxycholic acid (GDCA). (**F**) Taurocholic acid (TCA). (**G**) Taurodeoxycholic acid (TDCA). (**H**) Ursodeoxycholic acid (UDCA), One-Way ANOVA, * $p < 0.05$, ** $p < 0.01$, *** $p < 0.001$.

Fig. S3. Basal characterization of CMVMJD135 mice and pre-clinical trial. (**A**) Schematic representation of the timeline of the pre-clinical trial. Food supplementation with 0.4% TUDCA (w/w) was started at 5 weeks of age. Colored squares indicate performed tests at the indicated time-points. (**B**) Mean weight of consumed food per animal per week, in normally fed and TUDCA supplemented mice. A total of 8 to 10 cages were evaluated per condition. (**C**) Concentration of TUDCA in the serum, in µmol/l, of animals fed a normal or TUDCA-supplemented diet, at 34 weeks of age. A total of 6 animals with a normal diet and 11 animals with TUDCA supplementation were assessed. (**D**) Number of CAG repeats in the human *ATXN3* transgenic construct in treated and untreated CMVMJD135 mice. (**E**) Body weight progression of mice from the pre-treatment stage to the end of the trial. A total of 14 to 17 animals were evaluated per group. (**F**) Brain weight of mice at 34 weeks of age, after occision. A total of 4 to 5

animals were assessed per condition. Student's t-test for (B, C), Mann-Whitney U test for (D), One-Way ANOVA for (E, F), $*p < 0.05$, $*** p < 0.001$.

Fig. S4. Additional motor characterization of TUDCA-treated CMVMJD135 mice. (**A**) Evaluation of the time taken for a mouse to cross a 17 mm round beam, with TG animals being unable to perform the test at 26 weeks of age. (**B**) Time for a mouse to fall from a hanging wire, as a measure of muscular strength. (**C**) The presence and degree of severity of foot dragging was evaluated through footprinting, with TG TUDCA showing improvement in several time points. (**D**) The tonus of the hindlimbs was assessed qualitatively, with treated animals showing improvements in late stages of disease. (**E**) A qualitative assessment of tremors revealed a significant improvement of TG TUDCA-treated animals in comparison with TG. (**F**) Number of rears in a viewing jar, as a measure of vertical exploratory activity. A total number of 14 to 17 animals was used per group. A total of 14-17 mice per condition was used in all tests and evaluated in the indicated weeks of age. Black *, WT vs TG; red *, TG vs TG TUDCA. One-Way ANOVA for (A, B), Chi-Square test for (C-F), $* p < 0.05$, $** p < 0.01$, $*** p < 0.001$.

Fig. S5. Ataxin-3 aggregation is unaltered by TUDCA. (**A**) Immunohistochemistry for ataxin-3 (and respective quantification) reveals intranuclear inclusions/aggregates in the lateral reticular nucleus (LRT) and (**B**) pontine nuclei (PN) whose density per area is unchanged upon treatment with TUDCA. A total of 3 to 4 animals was evaluated per condition, with 3 to 6 sections used per animal. Student's t-test.

Fig. S6. Gene/protein levels of representative markers of proteostasis networks. (**A**) RT-qPCR of representative genes of the heat shock response, (**B**) antioxidant response, (**C**) ER stress and (**D**) autophagy. Brainstem tissue of 34 weeks-old mice was used, with 4 to 6 biological replicates per tested gene. (**E**) Western blot analysis of the levels of the 20S subunit of the proteasome, normalized to α-tubulin, in the brainstem of 34-week-old mice. A total of 3 biological replicates was tested. One-Way ANOVA was applied.

Fig. S7. Additional characterization of neuroinflammation and apoptosis in CMVMJD135 mice. (**A**) Additional inflammation genes' expression was assessed in the brainstem and spinal cord of 34 weeks-old mice, with expression values normalized for *B2m*. A total of 4-6 animals was tested per condition. (**B**) Western blot analysis (and respective quantification) of the microglial marker IBA-1 and (**C**) NF-κB pathway marker IKKβ, normalized to actin levels. A total of 3 biological replicates was tested per group. (**D**) Western blot analysis (and respective quantification) of the phosphorylation ratio of

p65. A total of 3 biological replicates was tested per group. (**E**) Western blot analysis (and respective quantification) of the anti-apoptotic protein TP53, represented by the ratio of phosphorylated-to-total TP53 levels. A total of 3 biological replicates per group was tested. (**F**) Expression levels of the pro-apoptotic marker caspase 3, through RT-qPCR, normalized for B2m. A total of 4-6 biological replicates was tested per group. One-Way ANOVA was applied.

Fig. S8. Additional transcriptomic and nematode/mouse functional data on the glucocorticoid receptor. (**A**) Venn diagram representing the total number of differentially expressed genes (including genes only predicted to be protein-coding) between WT, TG and/or TG TUDCA mice. The absolute number of genes and respective percentage (regarding the total number of differentially expressed genes in the analysis) is represented. (**B**) Gene expression of different transcripts measured by RNASeq, evaluated in the brainstem of 34 weeks-old mice, with 3-4 animals per group. In the RNASeq, expression of *Cox6b1* was decreased in TG TUDCA mice, expression of *Mat2b* was unchanged between groups, and expression of *Slc6a4* was increased in TG TUDCA mice, all of which are observed in the RT-qPCR (albeit without reaching statistical significance). (**C**) Gene expression of representative GR target genes in neuronal tissue, evaluated in the brainstem of 34 weeks-old mice, with 4 animals per group. (**D**) Drug toxicity was evaluated by measuring the optical density at 600 nm (OD_{600}) as a readout of OP50 consumption (food source), from day 0 (egg) to day 7. Treatments with 1% DMSO and 5% DMSO were used as non-toxic and toxic controls, respectively. Concentrations ranging from 0.0001 µM to 50 µM were tested for dexamethasone and (**E**) mifepristone. (**F**) Dose-response assay for the effects of mifepristone in the locomotion of N2 animals, using 1% DMSO was used as the negative control drug. A total of 150-200 animals were evaluated across 3-4 independent experiments. (**G**) Weights of mice from the preclinical trial with SCA3 animals co-treated with TUDCA and mifepristone. No statistical significant pairwise comparison was observed between any of the groups. A total of 11-15 mice per condition was assessed continuously in each time point. One-Way ANOVA was applied.

Fig. S9. Additional characterization of GR dysfunction in CMVMJD135 mice. (**A**) Representation of GR interaction with TUDCA in the antagonist binding conformation. The left figure represents the full view of the surface of the protein, while the right figure represents a slab view, with a cross-section along the surface of the protein to show the

accommodation of TUDCA inside the protein cavity. (**B**) Western blot analysis (and respective quantification) of the GR protein levels in the hippocampus, a non-affected region in SCA3. A total of 4 biological replicates per group was used, in 34-weeks-old mice. (**C**) Schematic representation of the timeline of the injections of TUDCA (50 mg/kg weight) in the acutely-treated mice. i.p., intraperitoneal. (**D**) Quantification of the levels of serum corticosterone by ELISA, of acutely treated mice, showing the expected increase in WT mice from the morning to the night period, as well as an overall increase in the levels in CMVMJD135 mice. A total of 5 animals was used per condition. (**E**) Expression levels of *GR*, *Fkbp5* and *Hsp90*, measured by RT-qPCR, in the brainstem of 34-week-old mice. A total of 4-6 biological replicates were assessed per group. One-Way ANOVA was applied, *** $p < 0.001$.

Fig. S10. Controls for the Tripartite split-GFP system. (**A**) Tripartite split GFP fluorescence in mammalian MRC5-SV cells expressing GFP1-9 transfected with GR fused with GFP10 and 14Q or (**B**) 78Q ATXN3 fused with GFP11, treated with Vehicle (Veh) or TUDCA at 25, 50 or 100 µM for 24h. (**C**) Tripartite split GFP fluorescence for the reverse assembly of GFP (GR fused with GFP11 and 14Q or 78Q ATXN3 fused with GFP10), showing the interaction is still present. (**D**) GR fused with GFP10 and 78Q ATXN3 fused with GFP11 treated with dexamethasone (DEX) at 10^{-6} M for 24h. As expected, a strong nuclear signal for GFP is observed, as dexamethasone is a well-known strong GR ligand. (**E**) Tubulin fused with GFP10 and 14Q or 78Q ATXN3 fused with GFP11, showing an already known interaction between tubulin and ATXN3. (**F**) Negative controls of the assay, showing signal absence upon two non-interacting proteins (GR and tubulin), (**G**) when GFP10 is not fused with any protein (empty) and (**H**) upon non-transfected cells. Green fluorescence at 488 nm excitation (GFP), DAPI nuclear staining (blue). Scale bars are 100 µm (20 µm in the insets).

Fig. S11. Additional correlations between peripheral gene expression and disease in SCA3 patients. (**A**) Partial correlation between the predicted time to disease onset (in pre-symptomatic patients) or disease duration (in symptomatic patients) with peripheral *GR* or (**B**) *FBKP5* expression, when adjusting for the sex of the patients. (**C**) Partial correlation between the predicted time to disease onset (in pre-symptomatic patients) or disease duration (in symptomatic patients) with peripheral *GR* or (**D**) *FBKP5* expression, when adjusting for the age of the patients. (**E**) Pearson's correlation between the peripheral expression of *GR* and *FKBP5* in pre-symptomatic and (**F**) symptomatic

patients. A total of 11 Pre-SCA3 (with 17 CTRL) and 30 SCA3 (with 20 CTRL) patients were assessed. Pearson Correlation Coefficient (r) was used.

Supplementary tables

Table S1. Statistical reports of all performed analyses.

Figure		Statistical report	Sample size
Fig. 1B		$F(7,19) = 7.897$, $p < 0.001$, $\omega^2 = 0.641$	3 or 4 (per group population)
Fig. 1D		Group: $F(1, 3) = 27.232$, $p < 0.001$, $\omega^2_p = 0.840$	14 to 17 (per group)
Fig. 1E		Group: $F(1, 3) = 24.630$, $p < 0.001$, $\omega^2_p = 0.825$	
Fig. 1F		$\chi^2 (3) = 56.844$, $p < 0.001$, $\varphi = 0.766$	
Fig. 1G		$\chi^2 (3) = 39.241$, $p < 0.001$, $\varphi = 0.359$	
Fig. 1H		$\chi^2 (6) = 54.808$, $p < 0.001$, $\varphi = 0.810$	
Fig. 2A		$F(2, 9) = 256.879$, $p < 0.001$, $\omega^2 = 0.977$	3 to 4 (per group)
Fig. 2B		$F(2, 9) = 6509.678$, $p < 0.001$, $\omega^2 = 0.999$	
Fig. 2C		$F(2, 9) = 5.107$, $p = 0.033$, $\omega^2 = 0.406$	
Fig. 2D		$F(2, 8) = 12.465$, $p = 0.003$, $\omega^2 = 0.676$	
Fig. 3A (Brainstem)	Arg1	$F(2, 9) = 15.370$, $p = 0.001$, $\omega^2 = 0.705$	4 to 6 (per group)
	Ccl2	$F(2, 9) = 7.894$, $p = 0.010$, $\omega^2 = 0.535$	
	Cd68	$F(2, 11) = 5.620$, $p = 0.021$, $\omega^2 = 0.398$	
	Cd86	$F(2, 12) = 7.756$, $p = 0.007$, $\omega^2 = 0.474$	
	Il10	$F(2, 11) = 6.292$, $p = 0.015$, $\omega^2 = 0.431$	
	Il1b	$F(2, 11) = 2.707$, $p = 0.011$, $\omega^2 = 0.196$	
	iNos2	$F(2, 11) = 0.209$, $p = 0.815$, $\omega^2 < 0$	
	Prdx2	$F(2, 10) = 4.263$, $p = 0.046$, $\omega^2 = 0.334$	
	Tnfa	$F(2, 12) = 4.968$, $p = 0.027$, $\omega^2 = 0.346$	
Fig. 3A (Spinal Cord)	Cxcl17	$F(2, 9) = 7.114$, $p = 0.014$, $\omega^2 = 0.505$	
	Il1b	$F(2, 9) = 7.386$, $p = 0.013$, $\omega^2 = 0.516$	
	Tnfa	$F(2, 11) = 3.999$, $p = 0.050$, $\omega^2 = 0.300$	
Fig. 3B		$F(2, 13) = 19.862$, $p < 0.001$, $\omega^2 = 0.702$	4 to 6 (per group population)
Fig. 3C		$F(2, 9) = 7.188$, $p = 0.014$, $\omega^2 = 0.508$	
Fig. 3D		$F(2, 8) = 1.528$, $p = 0.274$, $\omega^2 = 0.088$	
Fig. 3F		$F (2, 9) = 4{,}125$, $p = 0.0535$, $\omega^2 = 0.053$	15-32 astrocytes per group (n=3-4 animals/group)
Fig. 3G		$F (2, 8) = 3{,}930$, $p = 0.0647$, $\omega^2 = 0.091$	
Fig. 4F		$F(10, 39) = 30.860$, $p < 0.001$, $\omega^2 = 0.857$	4 to 6 (per group population)
Fig. 4G		$F(16, 79) = 7.137$, $p < 0.001$, $\omega^2 = 0.506$	4 to 6 (per group population)
Fig. 4H		$F(9, 29) = 11.091$, $p < 0.001$, $\omega^2 = 0.700$	3 or 4 (per group population)

Fig. 4I		F(9, 29) = 2.633, p = 0.023, ω^2 = 0.274	3 to 5 (per group population)
Fig. 4J		F(9, 27) = 12.512, p < 0.001, ω^2 = 0.767	3 to 4 (per group population)
Fig. 4K		F(9, 37) = 22.969, p < 0.001, ω^2 = 0.808	3 to 7 (per group population)
Fig. 5B		Group: F(1, 5) = 41.433, p < 0.001, ω^2_p = 0.741	11 to 15 (per group)
Fig. 5D		F(3, 12) = 5.744, p = 0.011, ω^2 = 0.471	4 (per group)
Fig. 5E		F(3, 12) = 4.416, p = 0.026, ω^2 = 0.390	
Fig. 6C		F(2, 6) = 12.280, p = 0.008, ω^2 = 0.715	3 (per group)
Fig. 6D		F(2, 9) = 5.952, p = 0.023, ω^2 = 0.452	4 (per group)
Fig. 6E		F(2,9) = 0.170, p = 0.846, ω^2 < 0	
Fig. 6F		F(2, 9) = 0.140, p = 0.871, ω^2 < 0	
Fig. 6G		H(2) = 5.808, p = 0.055	3 to 4 (per group)
Fig. 6H		t(4) = 0.957, p = 0.190, d = 0.782	3 (per group)
Fig. 7D		U = 36.0, p = 0.002, r = 0.832	12 (per group)
Fig. 7E		U = 36.0, p = 0.004, r = 0.810	12 (per group)
Fig. 8A		t(5) = 2.559, p = 0.049, d = 2.018	4 controls and 3 SCA3
Fig. 8B		t(5) = 0.759, p = 0.482, d = 0.581	
Fig. 8C	Pre-SCA3	t(26) = 0.262, p = 0.795, d = 0.093	17 CTRL and 11 Pre-SCA3
	SCA3	t(48) = 3.388, p = 0.001, d = 0.943	20 CTRL and 30 SCA3
Fig. 8D	Pre-SCA3	t(24) = 1.614, p = 0.120, d = 0.740	17 CTRL and 11 Pre-SCA3
	SCA3	U = 159.0, p = 0.005, r = 0.395	20 CTRL and 30 SCA3
Fig. 8E		r = - 0.153, p = 0.340	11 Pre-SCA3 and 30 SCA3
Fig. 8F		r = 0.704, p < 0.001	
Fig. 8G		r = - 0.189, p = 0.248	
Fig. 8H		r = - 0.743, p < 0.001	
Fig. 8I	Pre-SCA3	r = - 0.660, p = 0.027	
	SCA3	r = - 0.005, p = 0.805	
Fig. 8J	Pre-SCA3	r = - 0.317, p = 0.372	
	SCA3	r = - 0.060, p = 0.751	
Fig. S2A		F(9, 27) = 4.493, p = 0.001, ω^2 = 0.490	3 to 4 (per group population)
Fig. S2B		F(9, 19) = 7.453, p < 0.001, ω^2 = 0.667	
Fig. S2C		F(9, 19) = 6.790, p < 0.001, ω^2 = 0.642	
Fig. S2D		F(9, 20) = 18.506, p < 0.001, ω^2 = 0.840	
Fig. S2E		F(9, 20) = 6.926, p < 0.001, ω^2 = 0.640	
Fig. S2F		F(9, 19) = 10.667, p < 0.001, ω^2 = 0.750	
Fig. S2G		F(9, 25) = 18.631, p < 0.001, ω^2 = 0.819	
Fig. S2H		F(9, 30) = 4.629, p = 0.001, ω^2 = 0.449	
Fig. S3B		t(8) = 0.937, p = 0.376, d = 0.059	

Fig. S3C	$t(15) = 2.319, p = 0.035, d = 1.304$		6 ND and 11 TUDCA
Fig. S3D	$U = 115, p = 0.842, r=0.036$		15 to 16 (per group)
Fig. S3E	Group: $F(1, 3) = 26.429, p < 0.001, \omega^2_p = 0.836$		14 to 17 (per group)
Fig. S3F	$F(3, 14) = 9.533, p = 0.001, \omega^2 = 0.587$		4 to 5 (per group)
Fig. S4A	Group: $F(1, 3) = 5.714, p = 0.003, \omega^2_p = 0.485$		14 to 17 (per group)
Fig. S4B	Group: $F(1, 3) = 426.809, \omega^2_p = 0.988$		
Fig. S4C	$\chi^2 (6) = 311.595, p < 0.001, \varphi = 0.750$		
Fig. S4D	$\chi^2 (3) = 19.619, p < 0.001, \varphi = 0.238$		
Fig. S4E	$\chi^2 (6) = 19.993, p < 0.001, \varphi = 0.357$		
Fig. S4F	$\chi^2_F (5) = 169.143, p < 0.001$		
Fig. S5A	$t(5) = 0.213, p = 0.840, d = 0.192$		3 to 4 (per group)
Fig. S5B	$t(6) = 0.700, p = 0.510, d = 0.495$		
Fig. S6A	Hsp27	$F(2, 11) = 2.759, p = 0.107, \omega^2 = 0.201$	4 to 6 (per group)
	Hsp40	$F(2, 12) = 0.083, p = 0.921, \omega^2 < 0$	
	Hsp60	$F(2, 11) = 3.563, p = 0.064, \omega^2 = 0.268$	
	Hsp70	$F(2, 12) = 0.534, p = 0.600, \omega^2 < 0$	
	Hsp105	$F(2, 12) = 0.293, p = 0.751, \omega^2 < 0$	
Fig. S6B	Nrf2	$F(2, 10) = 0.463, p = 0.642, \omega^2 < 0$	
	Nqo1	$F(2, 12) = 0.068, p = 0.934, \omega^2 < 0$	
	Hmox1	$F(2, 12) = 0.109, p = 0.898, \omega^2 < 0$	
	Hif1a	$F(2, 9) = 0.17, p = 0.849, \omega^2 < 0$	
	Arnt	$F(2, 9) = 0.479, p = 0.634, \omega^2 < 0$	
Fig. S6C	Xbp1	$F(2, 11) = 1.194, p = 0.340, \omega^2 = 0.027$	
Fig. S6D	LC3	$F(2, 11) = 0.196, p = 0.825, \omega^2 < 0$	
	Becn1	$F(2, 11) = 0.386, p = 0.689, \omega^2 < 0$	
Fig. S6E	$F(2, 6) = 2.120, p = 0.201, \omega^2 = 0.199$		3 (per group)
Fig. S7A (Brainstem)	Cx3cr1	$F(2, 10) = 0.368, p = 0.701, \omega^2 < 0$	4 to 6 (per group)
	Cxcl10	$F(2, 10) = 0.097, p = 0.908, \omega^2 < 0$	
	Cxcl12	$F(2, 10) = 0.050, p = 0.951, \omega^2 < 0$	
	Cxcl14	$F(2, 12) = 0.139, p = 0.872, \omega^2 < 0$	
	Cxcl17	$F(2, 11) = 1.176, p = 0.345, \omega^2 = 0.025$	
	Gfap	$F(2, 12) = 0.075, p = 0.928, \omega^2 < 0$	
	Iba1	$F(2, 12) = 1.353, p = 0.295, \omega^2 = 0.045$	
	Il4	$F(2, 10) = 2.534, p = 0.129, \omega^2 = 0.191$	
	Il6	$F(2, 11) = 2.108, p = 0.168, \omega^2 = 0.137$	
	Mac2	$F(2, 10) = 1.972, p = 0.190, \omega^2 = 0.130$	
	MhcII	$F(2, 10) = 1.972, p = 0.190, \omega^2 = 0.130$	
	Nfkb1	$F(2, 9) = 0.490, p = 0.628, \omega^2 < 0$	
	Tgfb1	$F(2, 10) = 1.065, p = 0.381, \omega^2 = 0.010$	
	Cxcl10	$F(2, 9) = 1.083, 0.379, \omega^2 = 0.014$	
	Mac2	$F(2, 9) = 1.004, p = 0.404, \omega^2 = 0.001$	

Fig. S7A (Spinal Cord)	MhcII	$F(2, 9) = 0.095, p = 0.910, \omega^2 < 0$	
	Prdx2	$F(2, 9) = 0.095, p = 0.910, \omega^2 < 0$	
Fig. S7B		$F(2, 15) = 0.171, p = 0.844, \omega^2 < 0$	3 (per group)
Fig. S7C		$F(2, 6) = 1.156, p = 0.376, \omega^2 = 0.034$	
Fig. S7D		$F(2, 6) = 0.264, p = 0.777, \omega^2 < 0$	
Fig. S7E		$F(2, 6) = 0.388, p = 0.694, \omega^2 < 0$	
Fig. S7F		$F(2, 10) = 0.514, p = 0.613, \omega^2 < 0$	4 to 6 (per group)
Fig. S8B	Cox6b1	$F(2,6) = 1.797, p = 0.245, \omega^2 = 0.150$	3 to 4 (per group)
	Mat2b	$F(2,8) = 0.099, p = 0.099, \omega^2 < 0$	
	Slc6a4	$F(2,7) = 1.058, p = 0.397, \omega^2 = 0.024$	
Fig. S8C	Clpp	$F(2,9) = 2.849, p = 0.110, \omega^2 = 0.236$	4 per group
	Cox6a1	$F(2,9) = 3.106, p = 0.094, \omega^2 = 0.260$	
	Csrp2	$F(2,9) = 0.054, p = 0.948, \omega^2 < 0$	
	Cyp27a1	$F(2, 9) = 0.112, p = 0.896, \omega^2 < 0$	
	Efemp1	$F(2,9) = 1.632, p = 0.248, \omega^2 = 0.095$	
	Gabrb1	$F(2,9) = 0.713, p = 0.516, \omega^2 < 0$	
	Gadd45b	$F(2, 9) = 0.284, p = 0.759, \omega^2 < 0$	
	Gpd1	$F(2, 9) = 0.157, p = 0.857, \omega^2 < 0$	
	Hes5	$F(2,9) = 2.400, p = 0.146, \omega^2 = 0.189$	
	Hmgcs2	$F(2, 9) = 0.166, p = 0.850, \omega^2 < 0$	
	Idh2	$F(2, 9) = 0.129, p = 0.880, \omega^2 < 0$	
	Lgals1	$F(2,9) = 0.884, p = 0.446, \omega^2 < 0$	
	Pdk4	$F(2, 9) = 0.160, p = 0.854, \omega^2 < 0$	
	Pfdn2	$F(2,9) = 0.297, p = 0.750, \omega^2 < 0$	
	Plekhb1	$F(2,9) = 0.235, p = 0.795, \omega^2 < 0$	
	Ptgds	$F(2,9) = 0.588, p = 0.575, \omega^2 < 0$	
	Ptprd	$F(2,9) = 0.497, p = 0.624, \omega^2 < 0$	
	Sgk1	$F(2, 9) = 0.377, p = 0.696, \omega^2 < 0$	
	Stx8	$F(2,9) = 1.240, p = 0.335, \omega^2 = 0.038$	
Fig. S8F		$F(4, 15) = 1.972, p = 0.151, \omega^2 = 0.164$	3 or 4 (per group population)
Fig. S8G		Group: $F(1, 5) = 2,362, p = 0.048, \omega^2_p = 0.080$	11 to 15 (per group)
Fig. S9B		$F(2,9) = 1.373, p = 0.302, \omega^2 = 0.056$	4 per group
Fig. S9D		Group: $F(2, 10) = 5.813, p = 0.021, \omega^2_p = 0.425$	5 per group
Fig. S9E	Nr3c1	$F(2, 9) = 0.152, p = 0.861, \omega^2 < 0$	4 to 6 per condition
	Fkbp5	$F(2. 9) = 0.466, p = 0.642, \omega^2 < 0$	
	Hsp90	$F(2,13) = 25.004, p < 0.001, \omega^2 = 0.750$	
Fig. S11A		$r = - 0.093, p = 0.567$	11 Pre-SCA3 and 30 SCA3
Fig. S11B		$r = - 0.702, p < 0.001$	
Fig. S11C		$r = - 0.130, p = 0.424$	
Fig. S11D		$r = - 0.472, p = 0.002$	
Fig. S11E		$r = 0.686, p = 0.029$	
Fig. S11F		$r = 0.249, p = 0.185$	

Table S2. Statistical analysis of the lifespan assay.

**Condition**	**Median Lifespan** (s.e.m.)	**Mean Lifespan** (s.e.m.)	**Control Condition**	**Median Lifespan** (s.e.m.)	**Mean Lifespan** (s.e.m.)	**p-value** (Cox)	**Hazard Ratio** (95% CI)
N2 + TUDCA	**15** (0.261)	**15.716** (0.292)	N2 + DMSO	**14** (0.364)	**15.118** (0.282)	**0.241**	**0.873** [0.703, 1.093]
AT3Q130 + DMSO	**15** (0.684)	**15.677** (0.338)	N2 + DMSO	**14** (0.364)	**15.118** (0.282)	**0.128**	**0.844** [0.678, 1.050]
AT3Q130 + TUDCA	**17** (0.609)	**18.202** (0.464)	N2 + DMSO	**14** (0.364)	**15.118** (0.282)	< **0.001**	**0.509** [0.405, 0.641]
daf-2 LoF	**29** (1.239)	**29.181** (0.770)	N2 + DMSO	**14** (0.364)	**15.118** (0.282)	< **0.001**	**0.124** [0.092, 0.167]
daf-16 LoF	**12** (0.274)	**13.098** (0.218)	N2 + DMSO	**14** (0.364)	**15.118** (0.282)	< **0.001**	**1.722** [1.385, 2.140]
AT3Q130 + DMSO	**15** (0.684)	**15.677** (0.338)	AT3Q130 + TUDCA	**17** (0.609)	**18.202** (0.464)	< **0.001**	**0.604** [0.483, 0.755]

Table S3. Statistical analyses of non-treated vs TUDCA-treated WT animals (p-values).

			**Age (weeks)**													
			**4**	**6**	**10**	**12**	**14**	**16**	**18**	**20**	**22**	**24**	**26**	**30**	**32**	**34**
Body weight		**p-value (WT *vs* WT TUDCA)**	0.62	0.62	0.68	0.93	0.81	0.33	0.37	0.91	0.86	0.90	0.97	0.43	0.88	0.97
**Behavioral test**	Beam square 12 mm		0.78		0.75		0.27	0.72	0.14	0.10	0.15	0.56	0.90	0.40		
	Beam circle 17 mm		0.22		0.52		0.91	0.22	0.21	0.29	0.11	0.06	0.49			
	Motor swimming				0.68		0.94	0.36	0.06	0.51	0.57	0.16	0.30	0.30	0.45	0.12
	Foot dragging								1.00	1.00	1.00	1.00	1.00	1.00	1.00	1.00
	Quality of movement								1.00	1.00	1.00	1.00	0.48	1.00	1.00	1.00
	Hanging wire			0.38	0.30	0.86	0.18	0.33	0.10	0.33		0.33		0.58	0.50	0.52
	Hindlimb tonus			0.21		0.24		0.50		1.00		1.00		1.00		0.11
	Strength to grab			0.56		0.50		1.00		1.00		1.00		1.00		0.24
	No. of rears				0.83		0.50		0.17		0.87					0.65
	No. of squares in the arena								0.82	0.46	0.78	0.23	**0.03**	0.10	0.12	0.10
	Tremors				1.00		1.00		0.10		0.09		1.00			0.65
	Limb clasping								1.00	1.00	1.00	1.00	0.48	1.00	1.00	1.00

Table S4. Similarity between protein sequences of nematode and human nuclear hormone receptors.

**Nematode Gene**	**Predicted Human Orthologue**	**Amino Acid Identity (%)**
nhr-27	FXR	81.9
nhr-65		16.2
nhr-157		85.5
nhr-57	GR	58.8
nhr-81		57.1
nhr-82		48.2
nhr-93		79.5
nhr-257		36.0
nhr-261		29.7

Table S5. List of ChEMBL approved GR-binding molecules.

Attached.

Table S6. Information on patients whose brain samples were obtained.

Attached.

Table S7. Oligonucleotide sequences used in this study.

Attached.

Supplementary methods

C. elegans toxicity assay

The toxicity assay was carried out as previously described (1). All compounds were tested in 96 well plates prepared with WT animals, inactivated OP50 bacteria at approximately OD_{595} = 0.700 and the drug to test. The OD_{595} of each well was measured daily for 7 days and normalized for the number of animals in each well, as previously described (1). Wells with 5% DMSO were used as toxic compound controls, and 1% DMSO as non-toxic compound controls. All conditions were prepared in triplicate and an additional well was prepared without animals to exclude variations in OD_{595} of each individual compound.

C. elegans drug treatment and motility assay

Compound treatment was performed in liquid culture in a 96-well plate format, as previously described (1). In the day of the assay, 4-days old animals were transferred from the 96-well plates onto an unseeded NGM plate (equilibrated at 20°C). Plates were dried for 45 minutes to 1 hour before starting the assays. Motility assays were performed at 20°C as previously described (1). Animals remaining inside a 1-cm circle after one minute were scored as locomotion defective. Motor behavior assays were run in triplicate or quadruplicate, with a total of 150 to 200 animals tested per compound concentration.

C. elegans lifespan assay

For the lifespan experiment, TUDCA (1 µM in 1% DMSO) was diluted in inactivated OP50, prepared as previously described (1), and seeded onto NGM plates. 20 gravid adults were allowed to lay eggs for 3 hours in plates with the respective drugs. After 3 days, 100 synchronized young adults were passed to fresh plates and scored every 1-2

days thereon. Animals were censored if desiccated on the edge of plates, if under the agar, if they had extruded intestinal content or if internal hatching occurred. Animals were scored as dead if no mechanical response was present after light touching on the head 3 times. Plates were freshly prepared 2-3 times a week and animals transferred every day during the first 10 days, and every 2 days afterwards.

Mouse housing and health monitoring

All mice were exposed to the standard conditions of the room they were housed in: an artificial 12 h light/dark cycle (lights on from 8 a.m. to 8 p.m.), with a room temperature of 21 ± 1°C and a relative humidity of 50–60%. According to the internal rules of the animal facility, animals were fed with a standard diet (4RF25 throughout the gestation and postnatal periods, and 4RF21 in adulthood) (Mucedola SRL, Settimo Milanese, Italy) and water *ad libitum*. FELASA guidelines were followed regarding animals' health status, by using sentinel mice housed in the same animal rack (2, 3). Humane endpoints for the experiment were defined (20% reduction of the body weight, inability to reach food and water, presence of wounds in the body, dehydration). The humane endpoints were not reached as our experiments were performed before animals reached those conditions.

Mice treatment with TUDCA and mifepristone

For the chronic treatment, at 5 weeks of age the animals were sequentially assigned by cage into four groups of 15 animals each: CMVMJD135 and WT under normal diet and CMVMJD135 and WT under diet supplemented with 0.4% TUDCA. The treatment had the duration of 29 weeks, until the animals reached 34 weeks of age. This endpoint was chosen since CMVMJD135 mice under a standard diet were still able to reach the food

in the cage and perform most of the behavioral tests used in the study. Treatment started at 5 weeks of age since phenotype onset in this model occurs at 6 weeks of age.

An additional group of animals were used to perform an acute treatment. At week 33, animals were injected intraperitonially (i.p.) with 50 mg/kg of TUDCA (Sigma) or vehicle (NaCl 0.9%)), daily, for 7 days. A total of 5 animals was used per group (WT + vehicle, CMVMJD135 + vehicle, CMVMJD135 + TUDCA). Approximately 10 μL of blood were collected from the tail vein at 8 a.m. on day 4, and 8 p.m. of day 6 for quantification of corticosterone levels in the serum. This regimen was based on a previous observation that TUDCA is effective in a mouse model of Parkinson's disease after 3 daily injections (4). Animals were euthanized on day 7, at 34 weeks of age, by decapitation, with their brains being harvested, flash frozen in liquid nitrogen and stored at -80ºC for further molecular biology analyses.

To perform the TUDCA treatment + GR pharmacological inhibition studies, CMVMJD135 and WT-littermates, both male and female, were used. Drug administration started at 5 weeks of age and lasted until the age of 24 weeks. Vehicle (NaCl 0.9%) was administered i.p. for 5 days (once per day) to CMVMJD135 (n=13; 6 males (M), 7 females (F)) and WT mice (n=15; 6M, 9F). Mifepristone (MFP, Sigma) was administered i.p. once per week at 200 mg/kg to CMVMJD135 (n=13; 6M, 7F) and WT mice (n=11; 5M, 6F). TUDCA at 50 mg/kg was administered i.p. for 5 days (once per day) to CMVMJD135 mice (n=14; 6M, 8F). Co-treatment with MFP and TUDCA was administered using the same regimen as for single drug administration to CMVMJD135 mice (n=12; 6M, 6F).

Mouse behavioral phenotype assessment

Body weight: all mice were weighed from 4 to 34 weeks of age every two weeks.

Beam walk balance test: this test was used to assess balance and fine motor coordination, being performed as previously described (5). The test consisted of three days of training, where the animals had to cross the 12 mm squared beam three times/day. On day four, the mice were tested in the training beam (12 mm squared) and in a 17 mm rounded beam, two trials per animal. The time to cross the different beams was registered by the experimenter. Animals were allowed to fail twice during the test paradigm: either by falling or turning around on the beam.

Motor swimming test: to analyze their swimming skill, mice were trained for two consecutive days (3 trials per animal) to traverse a clear perspex water tank (60 cm) to a safe platform at the end. Water temperature was kept at 23ºC using a bath thermostat. Mice were tested for three consecutive days (2 trials per animal) and the latency to traverse the tank was registered by the experimenter.

Vertical movement assessment (rears): each mouse was placed in a viewing jar (15 cm diameter) for 5 minutes, and the number of vertical movements (including movements on and off the wall) was registered.

Spontaneous activity: the horizontal exploratory activity was measured in an open arena. This transparent arena was labeled with 15 squares (55 x 33 x 18 cm), and the number of squares travelled during one minute were manually counted by the researcher. Gait quality was also scored while animals were freely-walking in the area.

Footprint analysis: to quantitatively assess gait quality, footprints of each mouse were obtained by using non-toxic ink. The hind and forepaws of the mice were coated in different colors (black and red, respectively). Animals were encouraged to walk in an inclined runway (100 cm-long × 4.2 cm width × 10 cm height) over a clean paper sheet,

towards a safe black box. The presence and/or severity of foot dragging was longitudinally scored by assessing its presence in six consecutive steps, and was classified as follow: 0 = absent; 1 = mild, up to three steps; 2 = severe, more than three steps out of six.

Hanging wire grid test: mice were placed on the top of a metallic grid and slowly inverted 180º towards the surface of the bench; the cutoff of the test was 120 seconds, and the time each mouse took to fall (or if the animal was able to hang for the maximum time allowed) was annotated by the experimenter.

Neuropathology and immunohistochemistry

After being deeply anesthetized, mice were perfused with PBS followed by 4% paraformaldehyde (PFA) in PBS through the left ventricle of the heart. Brains were harvested and post fixed on PFA 4% solution for one 24 hours followed by paraffin embedding. Slides with 4 µm-thick sections were stained with cresyl violet or processed for immunohistochemistry with rabbit anti-GFAP (1:500, DAKO Corporation),rabbit anti-ataxin-3 1H9 (1:1000, Merck Millipore, Temecula, CA, USA) or goat anti-ChAT (1:1000, Merck Millipore, Temecula, CA, USA)

Ataxin-3 positive inclusions in the pontine nuclei (PN) and lateral reticular nucleus of the medulla (LRt) of 34 week old animals were quantified and normalized for total area using the Olympus BX51 stereological microscope (Olympus) and the Visiopharm integrator system software (Visiopharm). Brains and spinal cords of mice (n=4 per each condition) were sectioned in 40 µm-thick transverse sections using a LeicaVT100S vibratome (Leica Biosystems, Carnaxide, Portugal). ChAT-positive neurons in the spinal cord of 34 weeks old animals were quantified throughout the entire spinal cord using the Olympus BX51 stereological microscope (Olympus) and the Visiopharm integrator system software (Visiopharm). A total of 20-30 spinal cord sections per slide for each animal (n=4 per

group) were analyzed. GFAP densitometry in the substantia nigra and in the pontine nuclei was quantified and normalized for the total area using the Fiji software (ImageJ®).

RNA sequencing analysis

Results were analyzed using Transcriptome Analysis Console software v4.0 (Thermo Scientific, Waltham, Massachusetts). Principal component analysis (PCA) demonstrated partial overlap between TG and TUDCA mice, and a fully independent transcriptional profile of WT mice. The PCA retained 63.8% of explained variability of samples. Assessment of differential gene expression was carried out using a Bayesian one-way analysis of variance. Transcripts were considered differentially expressed if they simultaneously had a nominal p-value smaller than 0.010 and absolute fold-change of at least 1.500. The ENCODE Transcription Factor Targets database (6) was aligned with the desired transcripts to determine those that are targets of the GR (*NR3C1*) or the FXR (*NR1H4*).

Western blot analysis

Brain tissue was homogenized in cold RIPA buffer, to which a mixture of protease (Complete; Roche, Switzerland) and phosphatase inhibitors (Cocktail II and III, Sigma-Aldrich, Missouri, USA) was added, followed by sonication for 10 seconds in a bath sonicator. The Bradford assay (Bio-Rad, California, USA) was used to determine the protein concentration of each sample. Samples were heated for 5 minutes at 100°C and centrifuged for 10 seconds before loading. For each sample, 25 µg of total protein were loaded into SDS-PAGE gels followed by transference to nitrocellulose membranes (Bio-Rad, California, USA). Membranes were incubated with the respective primary

antibodies: rabbit anti-Proteasome 20S α+β (1:1000, Abcam, Cambridge UK), rabbit anti-GFAP (1:500, Dako, Denmark), mouse anti-GR (1:500, Santa Cruz Biotechnology, Texas, USA), rabbit anti-GR (1:200, Abcam, Cambridge, UK), rabbit anti-IBA-1 (1:500, Wako, Osaka, Japan), mouse anti-HSP90-β (1:1000, Enzo Life Sciences, Exeter, UK), rabbit anti-TP53 (1:1000, Cell Signaling, Massachusetts, USA), mouse anti-phospho-TP53 (1:1000, Cell Signaling, Massachusetts, USA), rabbit anti-H3 (1:1000, Abcam, Cambridge UK), mouse anti-GAPDH (1:500, Santa Cruz Biotechnology, Texas, USA), mouse anti-α-tubulin (1:200, DSHB, University of Iowa, Iowa) or mouse anti-β-actin (1:100, DSHB, University of Iowa, Iowa). Subsequently, membranes were probed with the respective secondary antibodies: anti-rabbit (1:10.000, Bio-Rad, California, USA) or anti-mouse (1:10.000, Bio-Rad, California, USA). Antibody affinity was detected by chemiluminescence (Clarity Western ECL, Bio-Rad, California, USA). Band quantification was performed using ImageLab® or AzureSpot, according to the manufacturer's instructions. For total protein level normalization in co-IP and TUBEs experiments, proteins were detected using a fluorescent dye (TotalStain Q, Azure biosystems, California, USA).

RT-qPCR analysis

Samples were treated with DNase I (Thermo Scientific, Waltham, Massachusetts) and the quality of RNA was evaluated by agarose gel electrophoresis. cDNA was synthesized using the iScript™ cDNA synthesis kit (Bio-Rad, California, USA). The qPCR was then performed using the 5x HOT FIREPol® EvaGreen® qPCR Mix Plus (ROX) (Solis BioDyne, Tartu, Estonia), as per the manufacturer's indications.

Subcellular fractionation of mouse tissue

Brain tissue was prepared in homogenization buffer (9% sucrose, 5 mM DTT, 2 mM EDTA, 25 mM Tris pH 7.4, complete protease inhibitor (Roche) and phosphatase inhibitor cocktails II and III (Sigma)) using a Dounce glass homogenizer, followed by centrifugation (10 min at 1000 rcf). The resulting supernatant was further centrifuged (15 min at 12,500 rcf), and its respective supernatant submitted to ultracentrifugation (1 h at 176,000 rcf), hence obtaining the final supernatant as the cytosolic fraction. The pellet from the first centrifugation was washed with 100 µL B1 buffer (0.32 M sucrose, 0.1 mM EGTA, 0.5 mM DTT, 1 mM HEPES, complete protease inhibitor (Roche) and phosphatase inhibitor cocktails II and III (Sigma)) and further centrifuged (10 min at 2000 rcf). The remaining pellet was washed with 200 µL B2 buffer (0.1 mM EGTA pH 7.0, 0.5 mM DTT, 10 mM HEPES, pH 7.9, 400 mM NaCl, 1.5 mM $MgCl_2$, 5% glycerol, complete protease inhibitor (Roche) and phosphatase inhibitor cocktails II and III (Sigma)), vortexed, incubated on ice for 30 min and centrifuged (15 min at 14,000 rcf), with the final supernatant corresponding to the nuclear fraction.

Enzyme-linked immunosorbent assay (ELISA)

For the measurement of corticosterone levels in mice, approximately 10 µL of tail vein blood was collected from mice at 8 a.m. and 2 days later, at 8 p.m. Samples were centrifuged, and the plasma supernatant was collected and used for the measurement. Corticosterone was measured using the Corticosterone ELISA Kit (Enzo Life Sciences, Farmingdale, NY, USA) accordingly to manufacturer's instructions. Concentrations were calculated based on a 4PL sigmoidal regression (with a least squared fit model) from Corticosterone standard samples.

Quantification of serum TUDCA levels

Mice with 34 weeks of age (chronically treated with TUDCA) were euthanized and blood was immediately collected. Samples were centrifuged and serum was collected for quantification of TUDCA levels, as previously described (7). Briefly, conjugated bile acids were extracted using chloroform/methanol followed by a column extraction using Lipidex 1000 (bed size, 4 x 1 x 1 cm; Packard Instrument Co., Groningen, The Netherlands). Bile acids were hydrolyzed and isolated from neutral sterols by lipophilic anion exchange chromatography. Derived methyl esters were separated by gas chromatography-mass spectrometry, and TUDCA was identified based on its retention index and mass spectrum.

Tripartite split GFP association assay

Cloning for the tripartite split GFP system

GR was amplified by PCR from pEGFP GR (Addgene #47504) with the synthetic oligonucleotides: 5’ – GGCGGCGGTGGATCTAGAATGGACTCCAAAGAATCA – 3’ (sense) and 5’ – TTAAACGGGCCCTCTAGTTCACTTTTGATGAAACAG – 3’ (antisense) for fusion with GFP10, and inserted into XbaI restriction site of modified pcDNA_GFP10-Nter vector. For fusion with GFP11, GR was amplified with the oligonucleotides 5’ – GCCACCATGGCTATCGCTATGGACTCCAAAGAATCA – 3’ (sense) and 5’ – GCCACCGCCACCATCGATCTTTTGATGAAACAGAAG – 3’ (antisense), and inserted into ClaI restriction site of modified pcDNA_GFP11-Cter. Wild-type (14Q) and mutant ATXN3 (78Q) were amplified from vector pBRIT TAP-ATXN3 14Q and pBRIT TAP-ATXN3 78Q isoform 2 (3 UIMs) generated in our laboratory, with the oligonucleotides: 5’ – CCTCCGGCGGCGGTGGATCTAGAATGGAGTCCATCTTCCACGAG – 3’ (sense) and 5’ –

TCAGCGGGTTTAAACGGGCCCTCTATTTTTTTCCTTCTGTTTTCAAATC – 3' (14Q) or TCAGCGGGTTTAAACGGGCCCTCTATTTTTTGCCTTCGGTTTTCAGGTC (78Q) (antisense), for fusion with GFP10. For fusion with GFP11, the following oligonucleotides were used: 5' – GGTGGAATTGCCACCATGGCTATGGAGTCCATCTTCCACGA – 3' (sense) and 5' – GACCCACCACCTCCAGAGCCACCGCCACCATCTTTTTTTCCTTCTGTTTTCAAATC – 3' (14Q) or 5' – GACCCACCACCTCCAGAGCCACCGCCACCATCTTTTTTGCCTTCGGTTTTCAGGTC – 3' (78Q) (antisense). Tubulin was amplified from cDNA of human SH-SY5Y cells with the oligonucleotides: 5' - CCTCCGGCGGCGGTGGATCTAGAATGCGTGAGTGCATCTCCATCCAC – 3' (sense) and 5' – TCAGCGGGTTTAAACGGGCCCTATGTATTCCTCTCCTTCTTCCTC – 3' (antisense) for the vector with GFP10, and with the oligonucleotides: 5' – GGAATTGCCACCATGGCTATCATGCGTGAGTGCATCTCCATCCA – 3' (sense) and 5' – ACCACCTCCAGAGCCACCGCCACCATCGTATTCCTCTCCTTCTTCCTC – 3' (antisense) for the vector with GFP11.

Cell fixation, staining, and microscopy analysis

After treatment, cells on coverslips were fixed with 4% PFA, permeabilized with 0.5% Triton X-100 in PBS buffer, and incubated with the DAPI counterstain (stock solution of 10 μg/mL) with a dilution of 1:2000. To analyze cells, coverslips were mounted with mounting medium (Epredi Lab Vision PermaFluor Aqueous Mounting Medium, TA-030-FM) and analyzed under Olympus LPS Confocal FV3000 microscope. Cells were imaged

using a 488-argon laser with a 490–553 nm emission filter (GFP), and with 405 UV diode laser for DAPI labelling. A 20x objective and a pinhole set at 1.0 Airy units was used, at a resolution of 1024 x 1024 pixels and z-stacking (step size of 1.5 μm). ImageJ® software was used for image processing. The area of GFP and DAPI signal colocalization was measured using a color threshold algorithm based on an HSB (hue, saturation and brightness) color space, using Li thresholding method, and manually defining the Hue vale. The overlap value was compared to total area of GFP signal.

Molecular modelling for protein-ligand docking

In preparing the structures for protein-ligand docking, water molecules and additional ligands were removed. Mifepristone, AZD9567 and INT-777 were saved for later used to optimize the docking protocol for each target, by redocking and comparing the predicted poses with the experimental ones by root mean square deviation (RMSD). Preparation of the structure of the targets and addition of the hydrogen atoms was performed in the GOLD software suite (8), using the recommended protocol, as in previous studies involving other protein targets (9). Structures for TUDCA, UDCA and taurine were downloaded from PUBCHEM (10), protonated for physiological pH using Openbabel (11), and optimized using Gaussian16 software (12).

Docking was performed with GOLD using the PLP scoring function (13), assuming a value over 60 as indicative of very strong binding (14). Mifepristone, AZD9567 and INT-777 were used as a reference to evaluate the accuracy of the docking protocol to each target and to optimize it. The conformation of each ligand was randomized, and the ligands were redocked against their initial target. Different settings were considered to ensure an accurate reproduction to the reference ligands with an RMSD below 1.5 Å. Final RMSD values were 0.83 Å, 0.41 Å and 1.16 Å for 3H52, 6EL9 and 7CFN

respectively, demonstrating high accuracy in reproducing experimentally confirmed binding poses. The optimized protocol with a binding pocket defined as centered on the initial cavity defined by each ligand, plus a radius of 20 Å was finally selected for each target, and applied in docking TUDCA, UDCA and taurine. To evaluate the TUDCA binding ability in a broader context, the optimized docking protocol was extended to 72 confirmed GR-binding drugs and clinical candidates, obtained from ChEMBL(15). A detailed list is presented in supporting information (Table S5), listing name, structure, date of first approval and corresponding references. The structures of these molecules were downloaded from ChEMBL, treated with Datawarrior (16), protonated for physiological pH using Openbabel and docked against both GR models using the same protocol used for TUDCA.

Human samples

Freshly frozen samples of pons, cerebellar cortex and dorsal striatum were used for protein and/or nucleic acid extraction. Proteins were extracted as aforementioned for mouse tissue. Nucleic acids were extracted using the AllPrep® DNA/RNA/miRNA kit (Qiagen, Hilden, Germany), by following the manufacturer's instructions. DNA was used in a PCR for quantification of the number of CAG repeats of each patient using the High Fidelity PCR Enzyme Mix (Thermo Scientific, Waltham, Massachusetts) and previously described primers MJD25a and MJD52 (17), followed by Sanger sequencing. RNA was used for cDNA synthesis and subsequent RT-qPCR, as described above. RNA was extracted from whole blood, treated with DNase, and reverse transcribed as previously described (18). The quantification of gene expression was performed as previously described for mouse tissue. Expression levels of *B2M* were used for normalization. For data analysis, the $2^{-\Delta\Delta Ct}$ method was used. The gene expression levels were normalized

for both housekeeping and control group. Individuals from the control group comprised apparently healthy individuals from the Azorean islands. This matching by population of origin was considered a priority, when comparing to age and sex matching, as the genetic background of isolated insular regions tends to be different from other populations.

The predicted age of clinical conversion in pre-symptomatic SCA3 patients was calculated based on the number of CAG repeats and age, as previously described (19). The predicted time to disease onset was determined by subtracting each patient's age at sample collection from the predicted age of clinical conversion.

Statistical analysis

Regarding continuous variables, the assumption of normality was assessed by qualitative analysis of Q-Q plots and frequency distributions, the z-score of skewness and kurtosis, as well as by the Kolmogorov-Smirnov and Shapiro-Wilk tests. The assumption of homogeneity of variances was tested by Levene's test and assumed for all variables. For repeated measurements, sphericity was tested using Mauchly's test, and assumed for all tested variables. Values that deviated more than 1.5 interquartile ranges from the mean were considered outliers and excluded from further analyses. For the comparison of means between 2 groups, the two-tailed unpaired Student's t-test or the Mann-Whitney U test was used (when data were normally or non-normally distributed, respectively). For the comparison of mean locomotion impairment in *C. elegans* experiments, a one-way analysis of variance (ANOVA) was used, followed by Dunnett's *post-hoc* test using AT3Q130 + DMSO as the control category. For the RNAi experiments, pairwise comparisons were performed using an orthogonal planned contrast analysis in the one-way ANOVA model. All other mean comparisons with more than 2 groups were carried out using a one-way ANOVA followed by Tukey's HSD *post-hoc* test, or a Kruskal-

Wallis test (when data were normally or non-normally distributed, respectively). Regarding the comparison of means with one between and one within-subjects factor, a mixed design ANOVA model was used, followed by Tukey's HSD *post-hoc* test for between-subjects variables. For the comparison of medians of discrete variables across time-points, a Friedman's ANOVA was carried out, with pairwise comparisons through the Kruskal-Wallis statistic. For the comparison of categorical variables, Fisher's exact test was used. *C. elegans* lifespan data was analyzed using a Cox regression model with the condition as a categorical covariable and a simple contrasts analysis. Correlations were carried out using Pearson's correlation coefficient. Effect size measurements are reported for all analyses (Cohen's d for t-tests, r for non-parametric tests and correlation, ω^2 or ω^2_p for ANOVAs, φ for categorical analyses and the hazard ratio for the survival analysis). TUDCA-treated WT mice were included in all mouse behavioral statistical analyses.

References

1. Teixeira-Castro A, et al. Serotonergic signalling suppresses ataxin 3 aggregation and neurotoxicity in animal models of Machado-Joseph disease. *Brain.* 2015;138(Pt 11):3221-37.
2. Nicklas W, et al. Recommendations for the health monitoring of rodent and rabbit colonies in breeding and experimental units. *Lab Anim.* 2002;36(1):20-42.
3. Guillen J. FELASA guidelines and recommendations. *J Am Assoc Lab Anim Sci.* 2012;51(3):311-21.
4. Rosa AI, et al. Tauroursodeoxycholic Acid Improves Motor Symptoms in a Mouse Model of Parkinson's Disease. *Mol Neurobiol.* 2018;55(12):9139-55.

5. Silva-Fernandes A, et al. Chronic treatment with 17-DMAG improves balance and coordination in a new mouse model of Machado-Joseph disease. *Neurotherapeutics.* 2014;11(2):433-49.
6. Consortium EP. The ENCODE (ENCyclopedia Of DNA Elements) Project. *Science.* 2004;306(5696):636-40.
7. Setchell KD, et al. Bile acid concentrations in human and rat liver tissue and in hepatocyte nuclei. *Gastroenterology.* 1997;112(1):226-35.
8. Jones G, et al. Development and validation of a genetic algorithm for flexible docking. *J Mol Biol.* 1997;267(3):727-48.
9. Quelhas D, et al. Assessing the effects of PMM2 variants on protein stability. *Mol Genet Metab.* 2021;134(4):344-52.
10. Kim S, et al. PubChem Substance and Compound databases. *Nucleic Acids Res.* 2016;44(D1):D1202-13.
11. O'Boyle NM, et al. Open Babel: An open chemical toolbox. *J Cheminform.* 2011;3:33.
12. Frisch MJ, et al. Wallingford, CT; 2016.
13. Korb O, et al. Empirical scoring functions for advanced protein-ligand docking with PLANTS. *J Chem Inf Model.* 2009;49(1):84-96.
14. Martins FG, et al. Identification of New Potential Inhibitors of Quorum Sensing through a Specialized Multi-Level Computational Approach. *Molecules.* 2021;26(9).
15. Gaulton A, et al. ChEMBL: a large-scale bioactivity database for drug discovery. *Nucleic Acids Res.* 2012;40(Database issue):D1100-7.
16. Sander T, et al. DataWarrior: an open-source program for chemistry aware data visualization and analysis. *J Chem Inf Model.* 2015;55(2):460-73.

17. Maciel P, et al. Correlation between CAG repeat length and clinical features in Machado-Joseph disease. *Am J Hum Genet.* 1995;57(1):54-61.

18. Raposo M, et al. Novel candidate blood-based transcriptional biomarkers of Machado-Joseph disease. *Mov Disord.* 2015;30(7):968-75.

19. Tezenas du Montcel S, et al. Prediction of the age at onset in spinocerebellar ataxia type 1, 2, 3 and 6. *J Med Genet.* 2014;51(7):479-86.

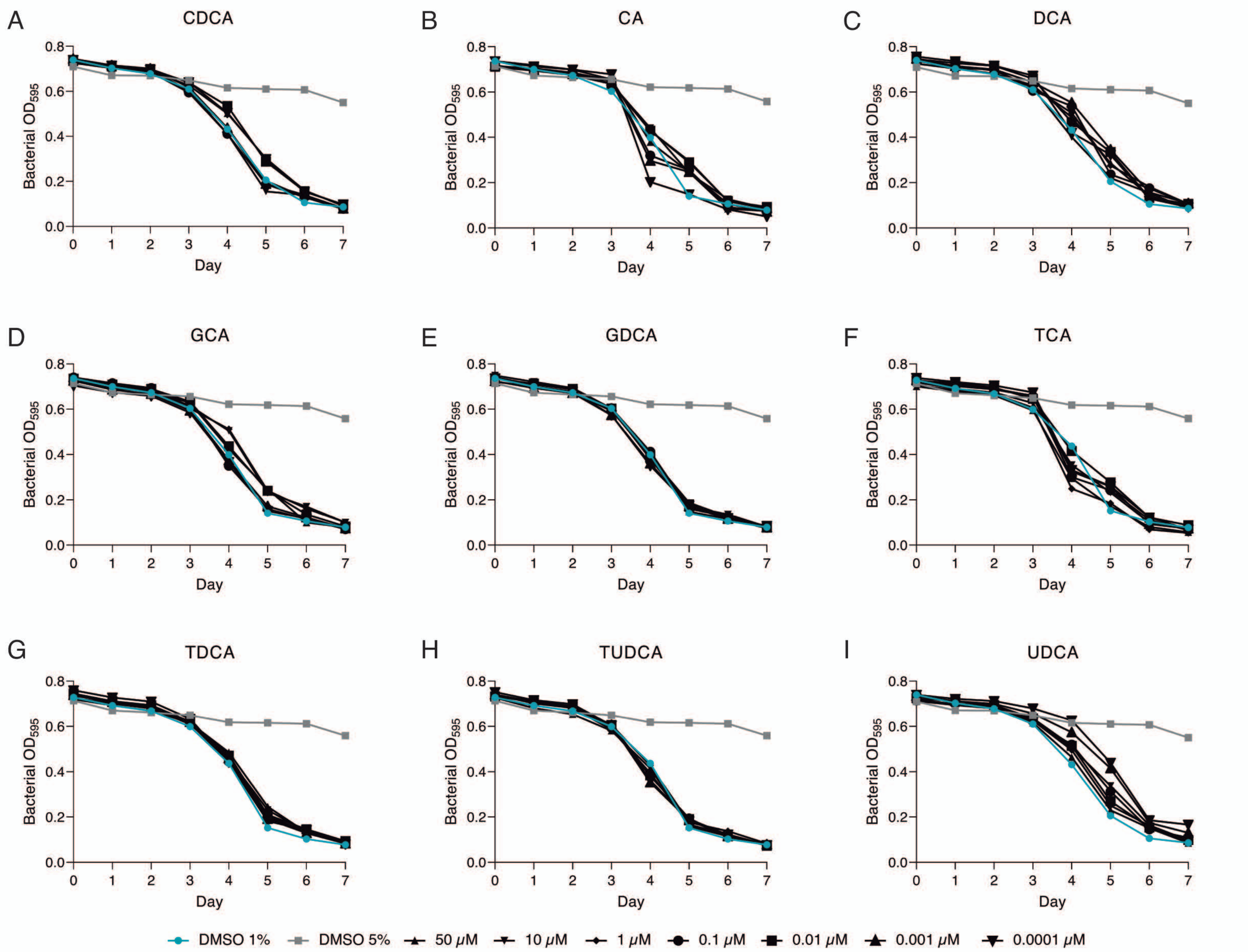
A
CDCA
B
CA
C
DCA
D
GCA
E
GDCA
F
TCA
G
TDCA
H
TUDCA
I
UDCA
Bacterial OD595
Day
DMSO 1%
DMSO 5%
50 μM
10 μM
1 μM
0.1 μM
0.01 μM
0.001 μM
0.0001 μM

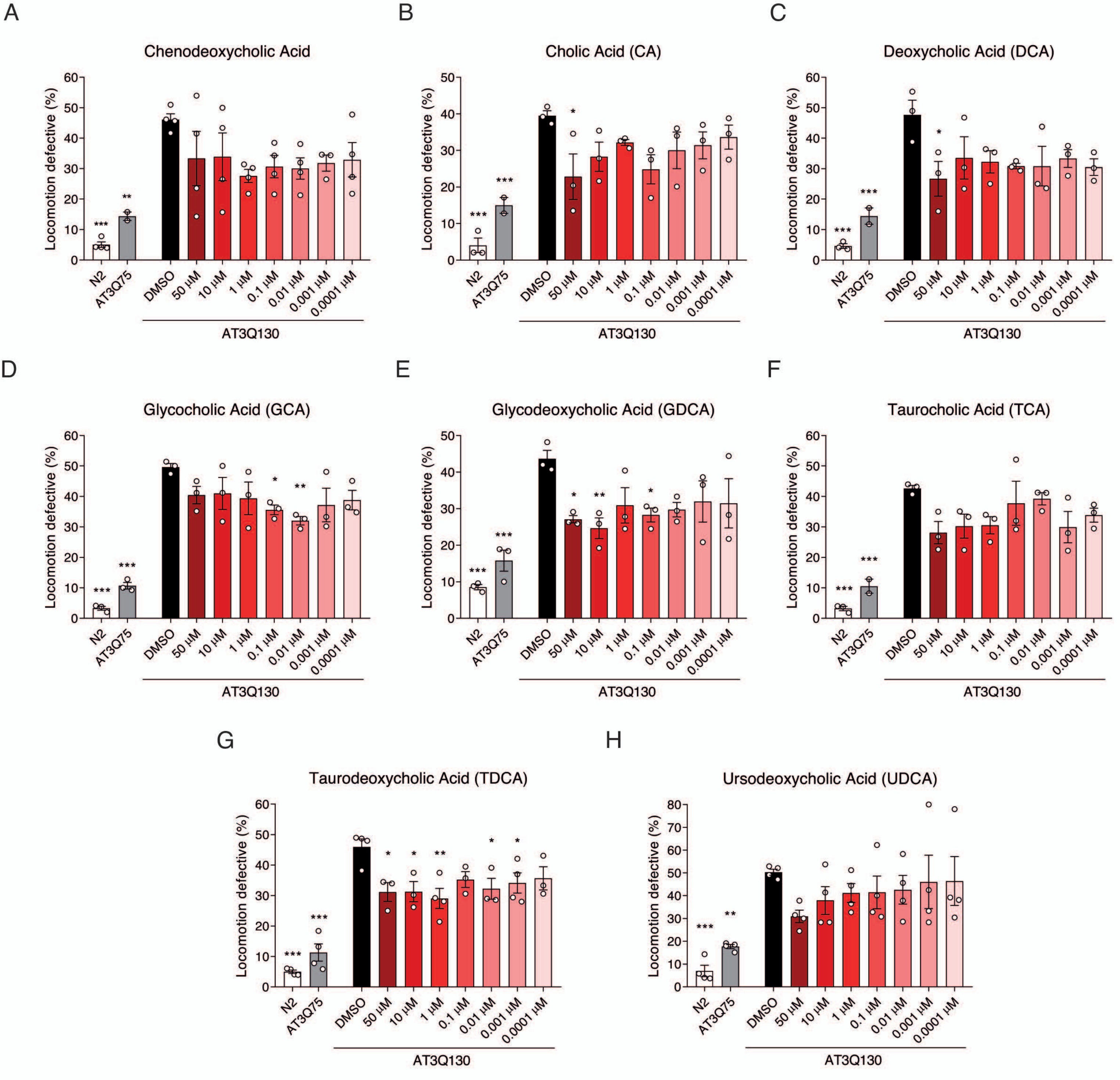
A
Chenodeoxycholic Acid
B
Cholic Acid (CA)
C
Deoxycholic Acid (DCA)
D
Glycocholic Acid (GCA)
E
Glycodeoxycholic Acid (GDCA)
F
Taurocholic Acid (TCA)
G
Taurodeoxycholic Acid (TDCA)
H
Ursodeoxycholic Acid (UDCA)
Locomotion defective (%)
N2
AT3Q75
DMSO
50 μM
10 μM
1 μM
0.1 μM
0.01 μM
0.001 μM
0.0001 μM
AT3Q130

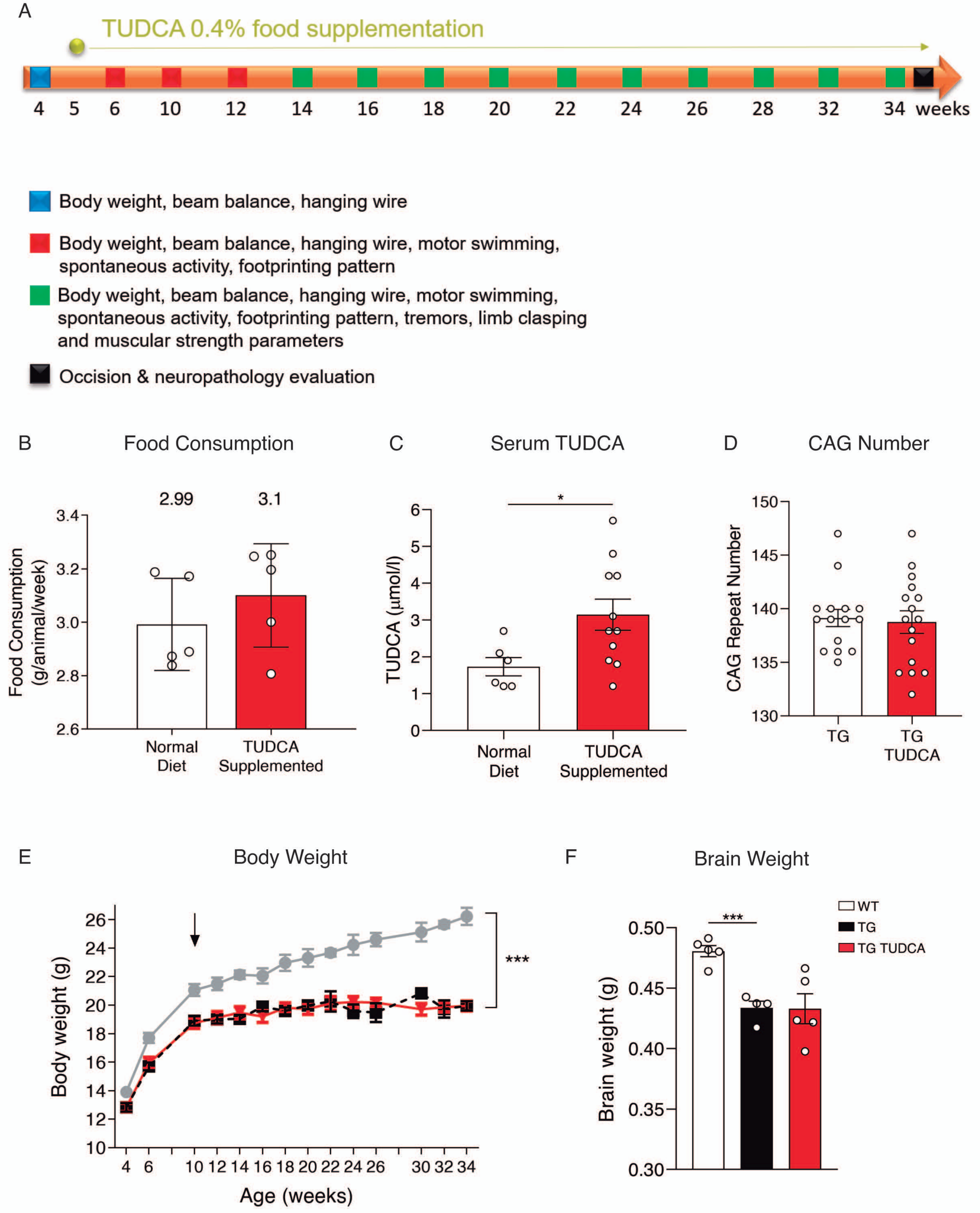
A
TUDCA 0.4% food supplementation
4 5 6 10 12 14 16 18 20 22 24 26 28 32 34 weeks
Body weight, beam balance, hanging wire
Body weight, beam balance, hanging wire, motor swimming, spontaneous activity, footprinting pattern
Body weight, beam balance, hanging wire, motor swimming, spontaneous activity, footprinting pattern, tremors, limb clasping and muscular strength parameters
Occision & neuropathology evaluation
B
Food Consumption
2.99
3.1
Food Consumption (g/animal/week)
3.4
3.2
3.0
2.8
2.6
Normal Diet
TUDCA Supplemented
C
Serum TUDCA
TUDCA (µmol/l)
*
6
5
4
3
2
1
0
Normal Diet
TUDCA Supplemented
D
CAG Number
CAG Repeat Number
150
145
140
135
130
TG
TG TUDCA
E
Body Weight
Body weight (g)
26
24
22
20
18
16
14
12
10
***
4 6 10 12 14 16 18 20 22 24 26 30 32 34
Age (weeks)
F
Brain Weight
Brain weight (g)
0.50
0.45
0.40
0.35
0.30
***
WT
TG
TG TUDCA

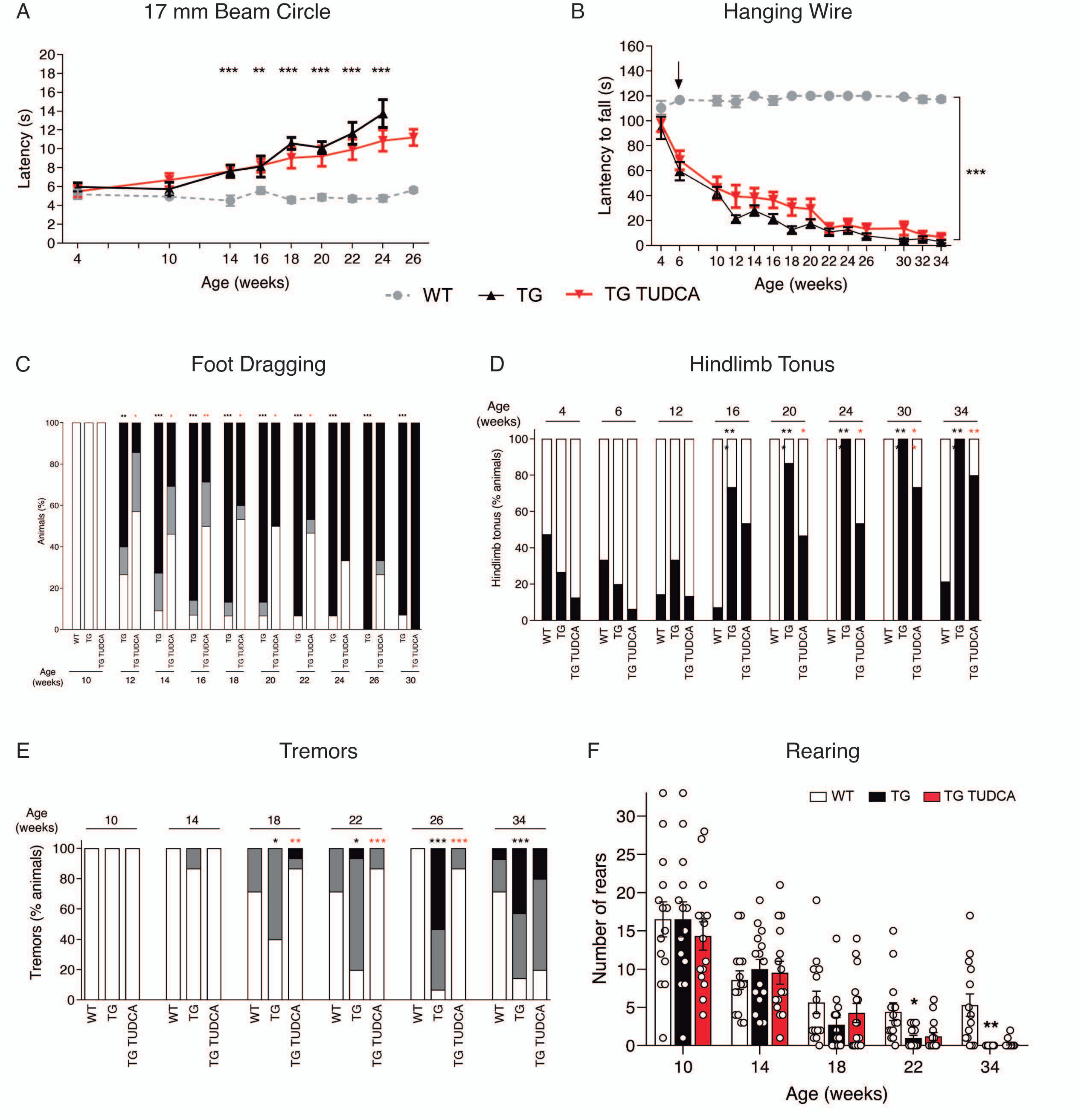
A
17 mm Beam Circle
Latency (s)
Age (weeks)
B
Hanging Wire
Lantency to fall (s)
Age (weeks)
WT
TG
TG TUDCA
C
Foot Dragging
Animals (%)
Age (weeks)
D
Hindlimb Tonus
Age (weeks)
Hindlimb tonus (% animals)
E
Tremors
Age (weeks)
Tremors (% animals)
F
Rearing
Number of rears
Age (weeks)

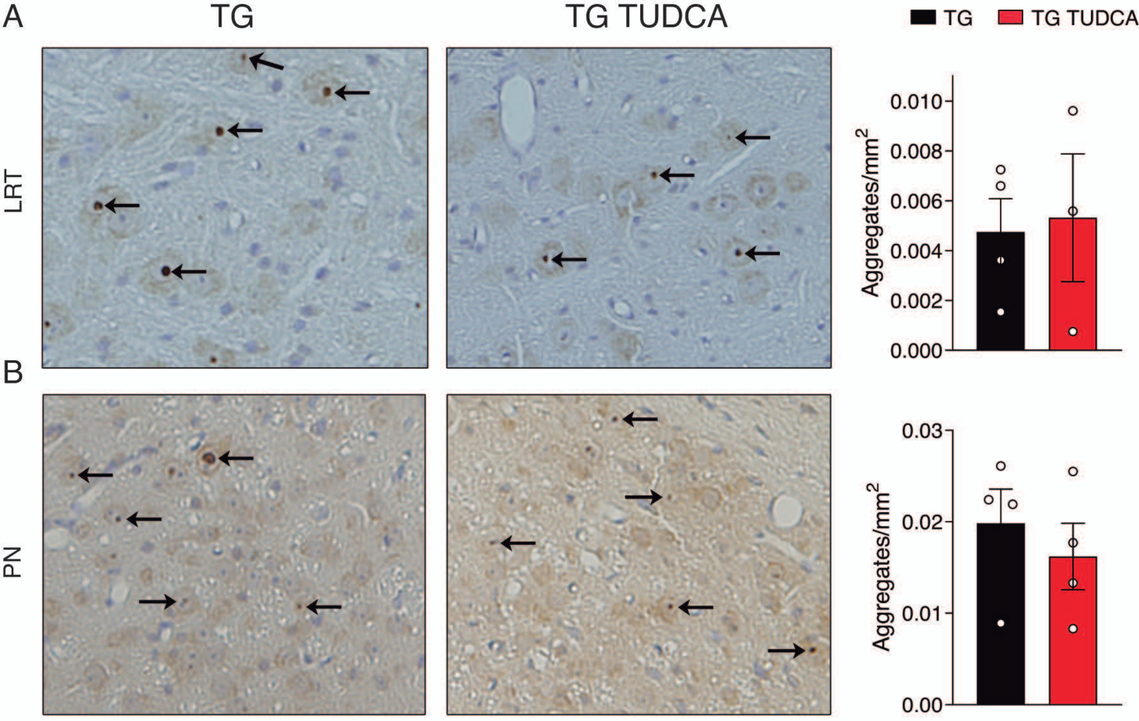
A
TG
TG TUDCA
TG
TG TUDCA
LRT
Aggregates/mm^2
0.010
0.008
0.006
0.004
0.002
0.000
B
PN
Aggregates/mm^2
0.03
0.02
0.01
0.00

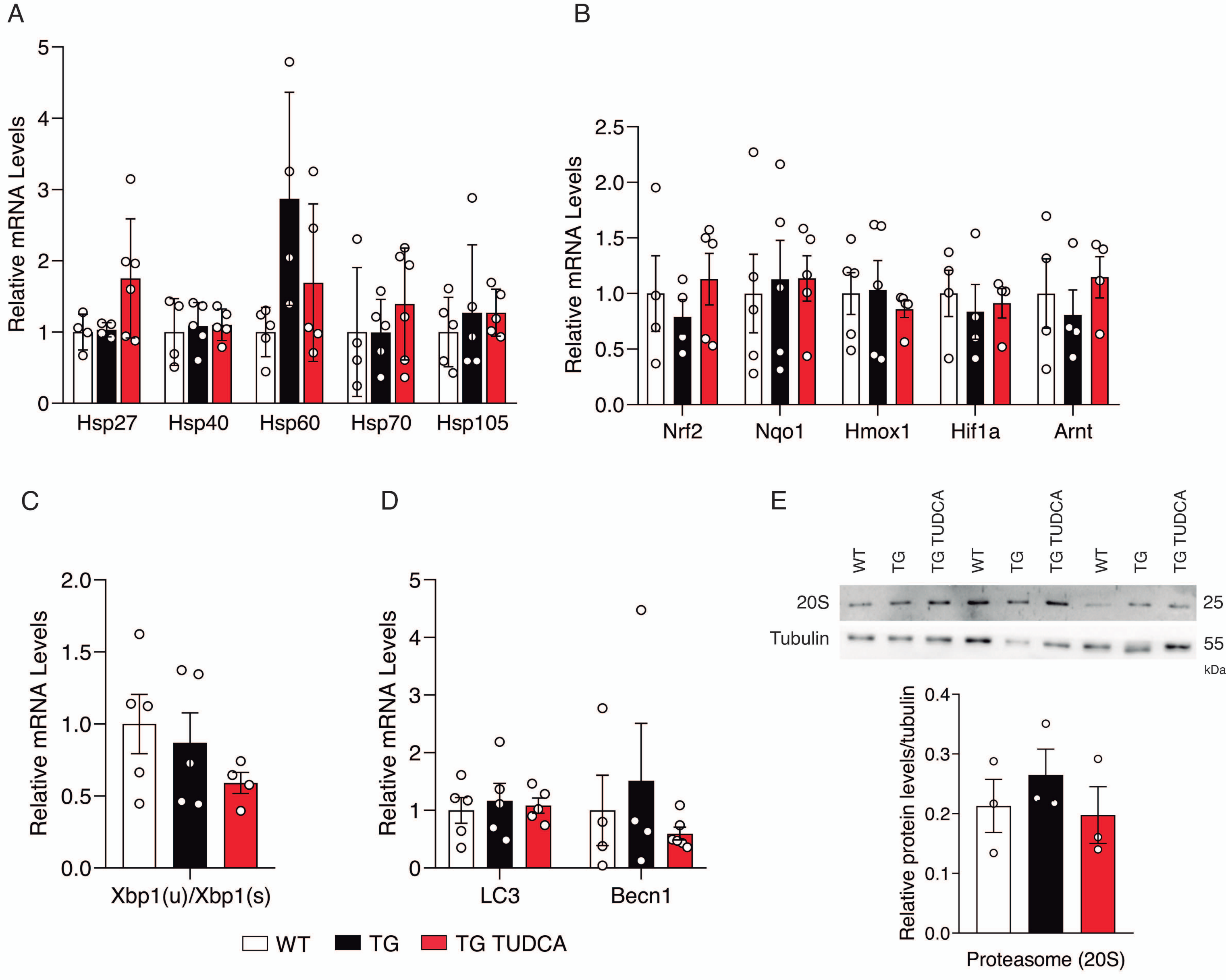
A
Relative mRNA Levels
Hsp27
Hsp40
Hsp60
Hsp70
Hsp105
B
Relative mRNA Levels
Nrf2
Nqo1
Hmox1
Hif1a
Arnt
C
Relative mRNA Levels
Xbp1(u)/Xbp1(s)
D
Relative mRNA Levels
LC3
Becn1
E
WT
TG
TG TUDCA
20S
Tubulin
25
55
kDa
Relative protein levels/tubulin
Proteasome (20S)
WT
TG
TG TUDCA

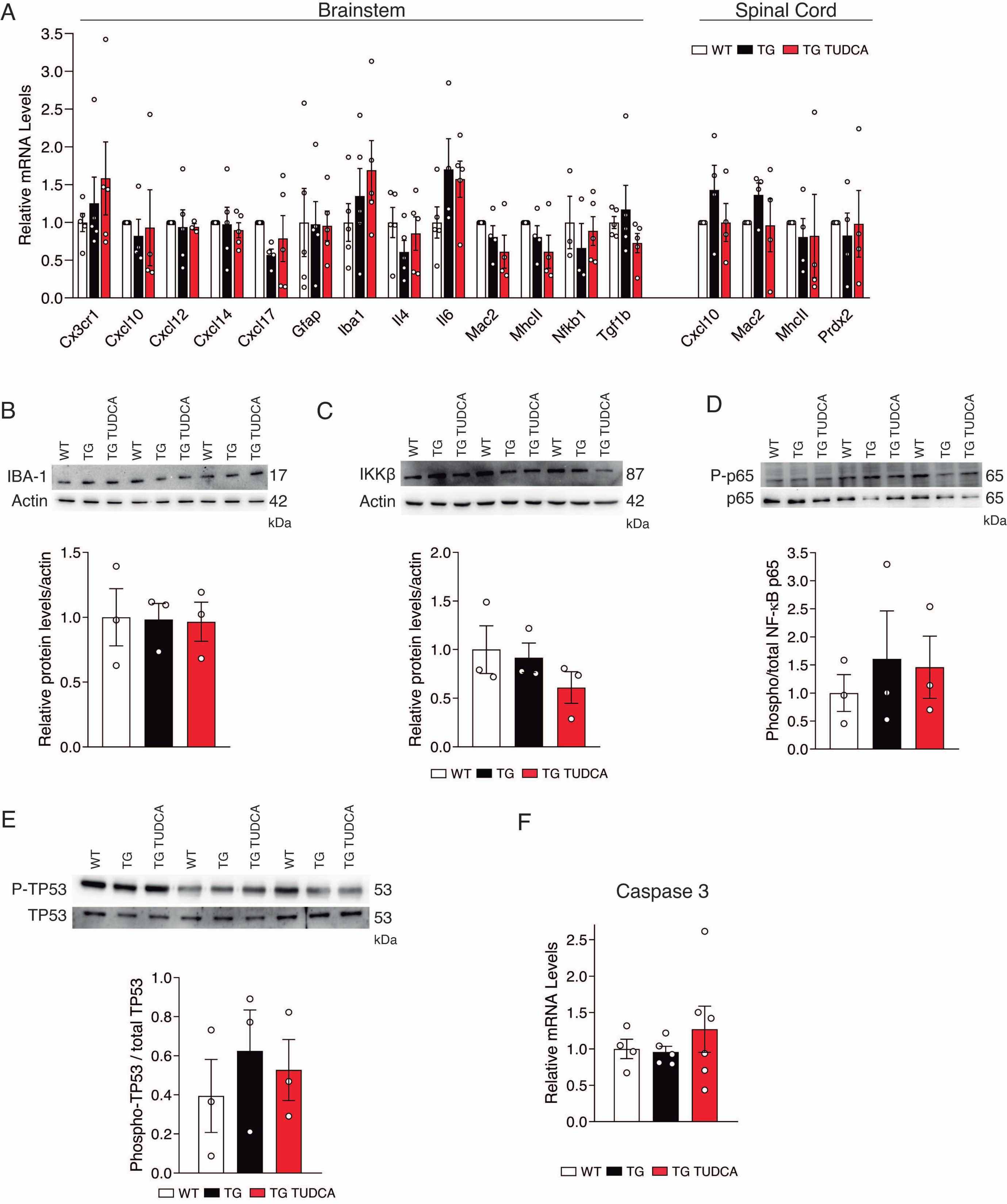
A
Brainstem
Spinal Cord
WT TG TG TUDCA
Relative mRNA Levels
Cx3cr1 Cxcl10 Cxcl12 Cxcl14 Cxcl17 Gfap Iba1 Il4 Il6 Mac2 MhcII Nfkb1 Tgf1b Cxcl10 Mac2 MhcII Prdx2
B
WT TG TG TUDCA WT TG TG TUDCA WT TG TG TUDCA
IBA-1 17
Actin 42
kDa
Relative protein levels/actin
C
IKKβ 87
Actin 42
kDa
Relative protein levels/actin
WT TG TG TUDCA
D
P-p65 65
p65 65
kDa
Phospho/total NF-κB p65
E
P-TP53 53
TP53 53
kDa
Phospho-TP53 / total TP53
WT TG TG TUDCA
F
Caspase 3
Relative mRNA Levels
WT TG TG TUDCA

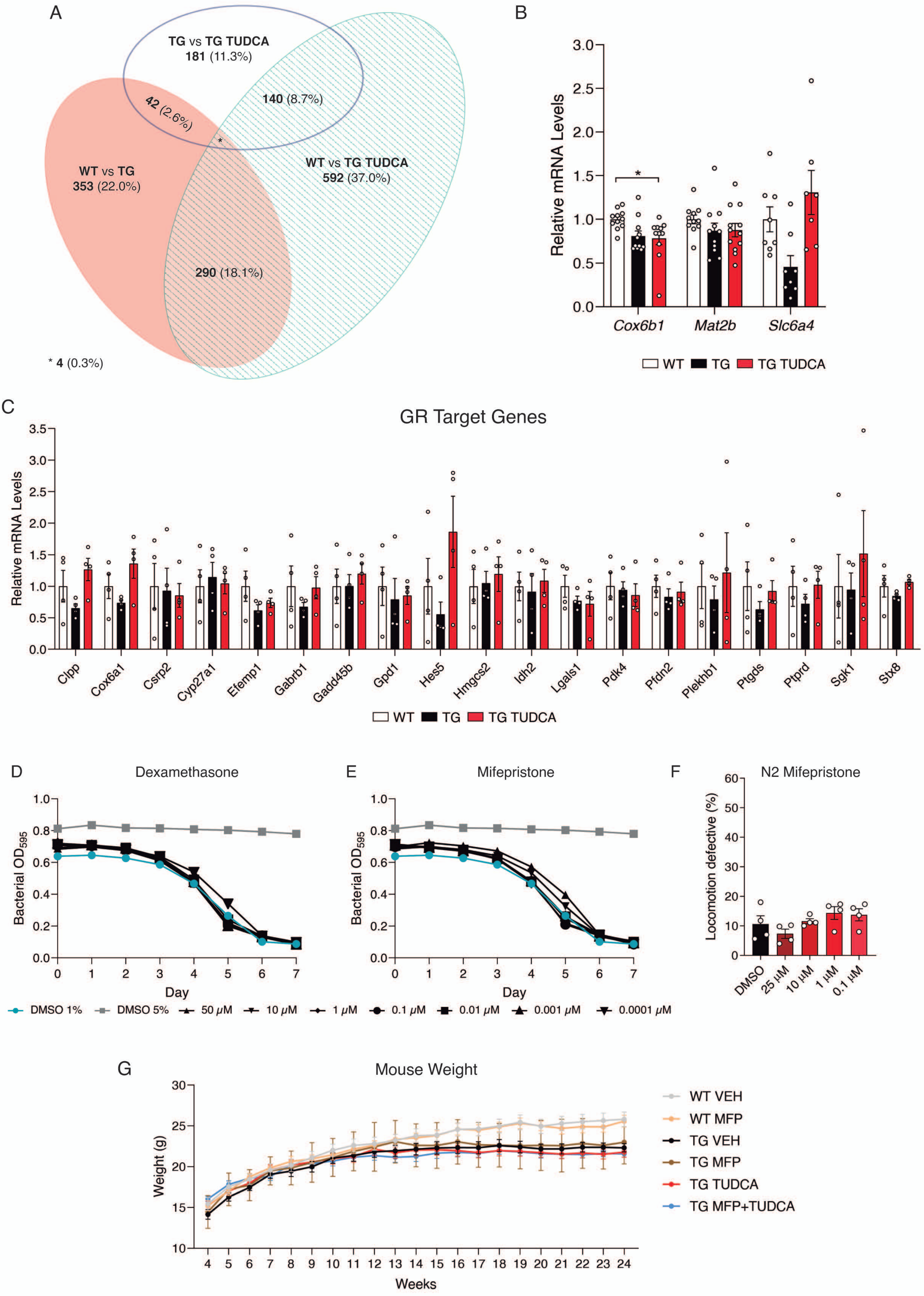

A
TG vs TG TUDCA
181 (11.3%)
42 (2.6%)
140 (8.7%)
WT vs TG
353 (22.0%)
WT vs TG TUDCA
592 (37.0%)
290 (18.1%)
* 4 (0.3%)
B
Relative mRNA Levels
Cox6b1
Mat2b
Slc6a4
WT
TG
TG TUDCA
C
GR Target Genes
Relative mRNA Levels
Clpp
Cox6a1
Csrp2
Cyp27a1
Efemp1
Gabrb1
Gadd45b
Gpd1
Hes5
Hmgcs2
Idh2
Lgals1
Pdk4
Pfdn2
Plekhb1
Ptgds
Ptprd
Sgk1
Stx8
WT
TG
TG TUDCA
D
Dexamethasone
Bacterial OD595
Day
E
Mifepristone
Bacterial OD595
Day
F
N2 Mifepristone
Locomotion defective (%)
DMSO
25 μM
10 μM
1 μM
0.1 μM
DMSO 1%
DMSO 5%
50 μM
10 μM
1 μM
0.1 μM
0.01 μM
0.001 μM
0.0001 μM
G
Mouse Weight
Weight (g)
Weeks
WT VEH
WT MFP
TG VEH
TG MFP
TG TUDCA
TG MFP+TUDCA

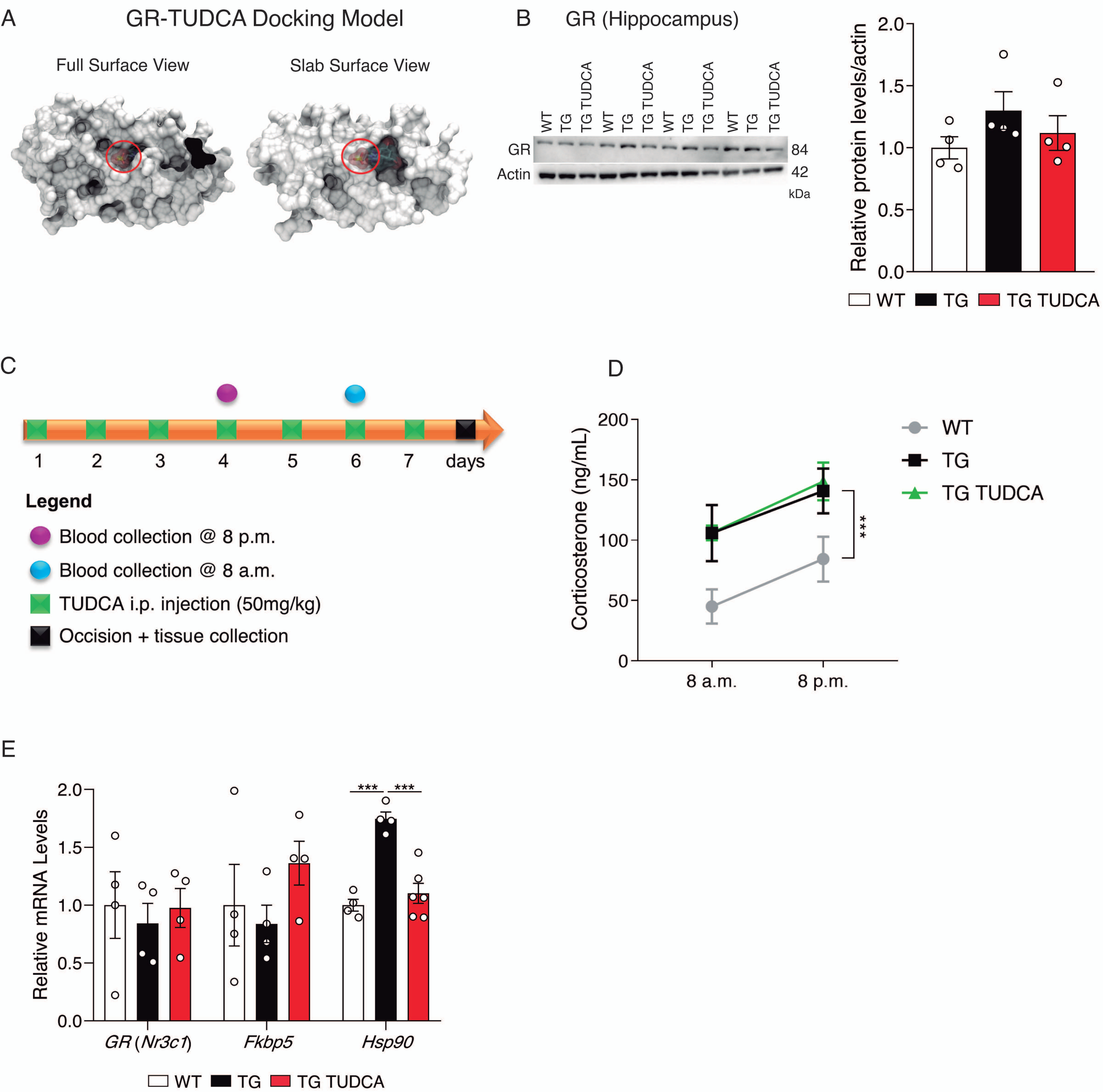
A
GR-TUDCA Docking Model
Full Surface View
Slab Surface View
B
GR (Hippocampus)
WT
TG
TG TUDCA
GR
Actin
84
42
kDa
Relative protein levels/actin
2.0
1.5
1.0
0.5
0.0
WT
TG
TG TUDCA
C
1
2
3
4
5
6
7
days
Legend
Blood collection @ 8 p.m.
Blood collection @ 8 a.m.
TUDCA i.p. injection (50mg/kg)
Occision + tissue collection
D
Corticosterone (ng/mL)
200
150
100
50
0
8 a.m.
8 p.m.
***
WT
TG
TG TUDCA
E
Relative mRNA Levels
2.0
1.5
1.0
0.5
0.0
GR (Nr3c1)
Fkbp5
Hsp90
***
***
WT
TG
TG TUDCA

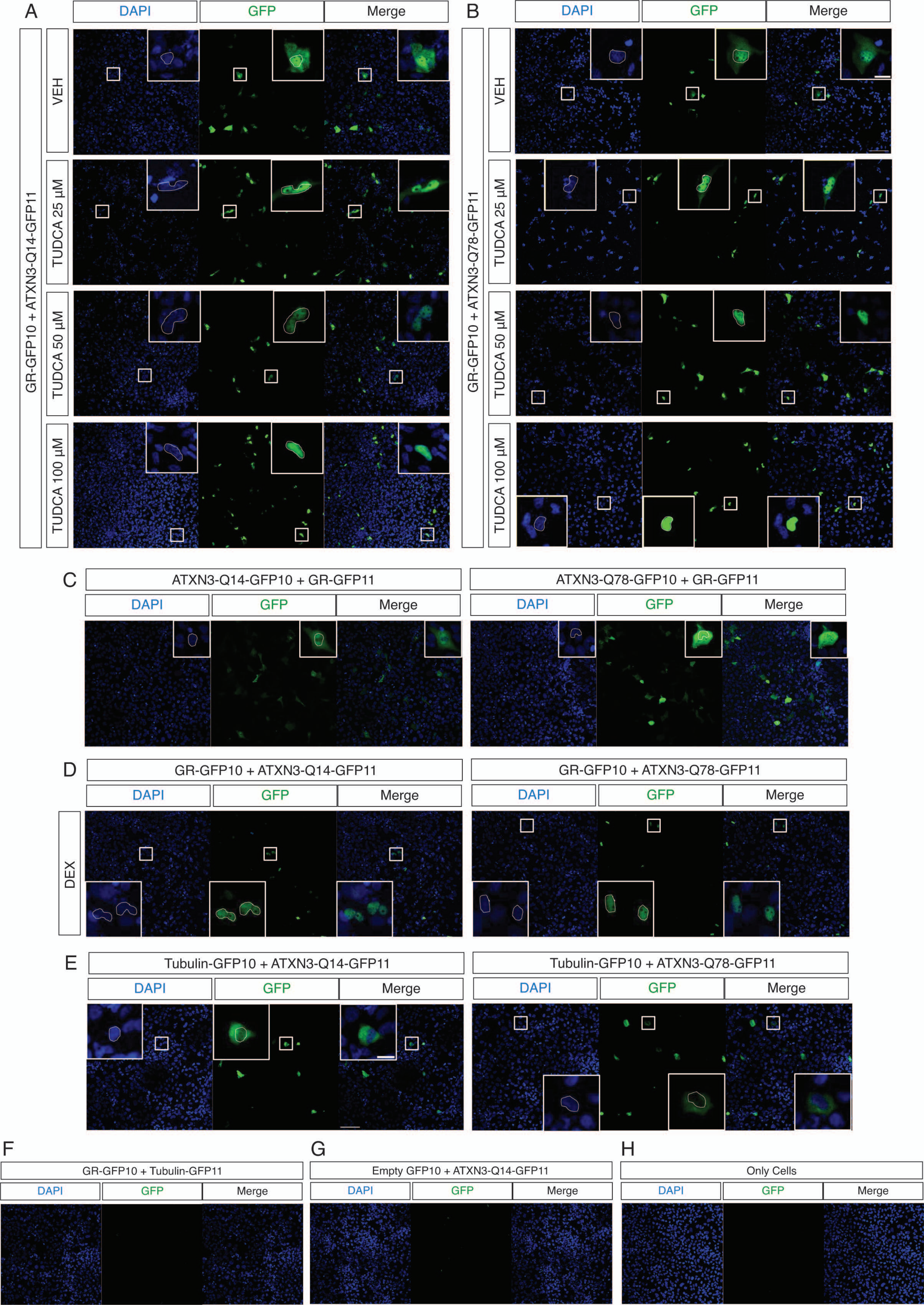
A
DAPI
GFP
Merge
GR-GFP10 + ATXN3-Q14-GFP11
VEH
TUDCA 25 µM
TUDCA 50 µM
TUDCA 100 µM
B
DAPI
GFP
Merge
GR-GFP10 + ATXN3-Q78-GFP11
VEH
TUDCA 25 µM
TUDCA 50 µM
TUDCA 100 µM
C
ATXN3-Q14-GFP10 + GR-GFP11
ATXN3-Q78-GFP10 + GR-GFP11
DAPI
GFP
Merge
D
GR-GFP10 + ATXN3-Q14-GFP11
GR-GFP10 + ATXN3-Q78-GFP11
DAPI
GFP
Merge
DEX
E
Tubulin-GFP10 + ATXN3-Q14-GFP11
Tubulin-GFP10 + ATXN3-Q78-GFP11
DAPI
GFP
Merge
F
GR-GFP10 + Tubulin-GFP11
DAPI
GFP
Merge
G
Empty GFP10 + ATXN3-Q14-GFP11
DAPI
GFP
Merge
H
Only Cells
DAPI
GFP
Merge

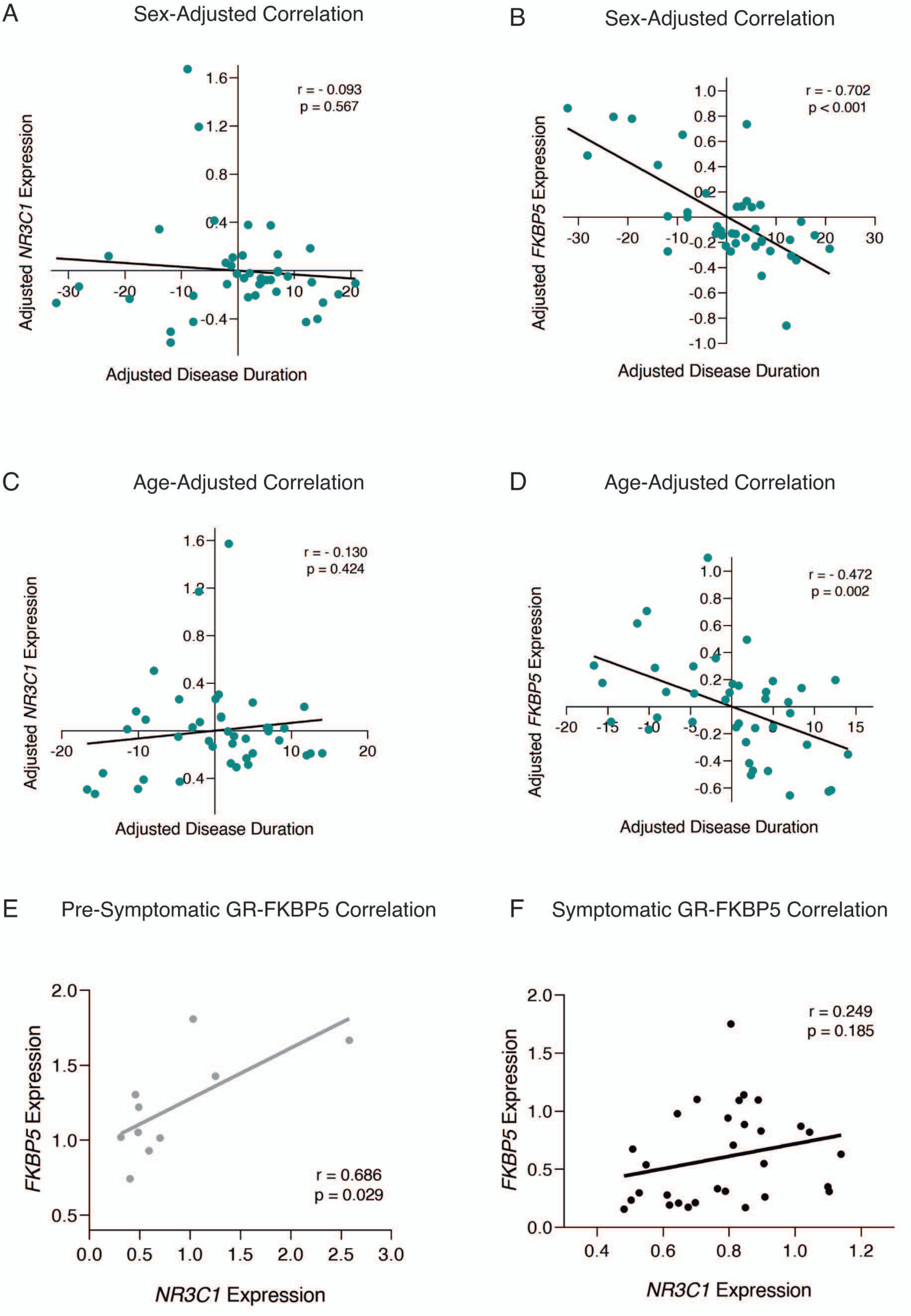
A
Sex-Adjusted Correlation
r = - 0.093
p = 0.567
Adjusted NR3C1 Expression
Adjusted Disease Duration
B
Sex-Adjusted Correlation
r = - 0.702
p < 0.001
Adjusted FKBP5 Expression
Adjusted Disease Duration
C
Age-Adjusted Correlation
r = - 0.130
p = 0.424
Adjusted NR3C1 Expression
Adjusted Disease Duration
D
Age-Adjusted Correlation
r = - 0.472
p = 0.002
Adjusted FKBP5 Expression
Adjusted Disease Duration
E
Pre-Symptomatic GR-FKBP5 Correlation
r = 0.686
p = 0.029
FKBP5 Expression
NR3C1 Expression
F
Symptomatic GR-FKBP5 Correlation
r = 0.249
p = 0.185
FKBP5 Expression
NR3C1 Expression
