# Supplementary material for: Glucocorticoid receptor-dependent therapeutic efficacy of tauroursodeoxycholic acid in preclinical models of spinocerebellar ataxia type 3
Source: J Clin Invest. 2024 Mar 1;134(5):e162246. doi: 10.1172/JCI162246 (PMC10904051; doi:10.1172/JCI162246)

# Uncropped Blots

## Glucocorticoid receptor dysfunction as a biomarker and target for bile acid therapy in SCA3/MJD

Sara Duarte-Silva[1,2,§], Jorge Diogo Da Silva[1,2,3,4,§], Daniela Monteiro-Fernandes[1,2,§], Marta Daniela Costa[1,2], Andreia Neves-Carvalho[1,2], Mafalda Raposo[5], Carina Soares-Cunha[1,2], Joana S. Correia[1,2], Gonçalo Nogueira-Goncalves[1,2], Henrique S. Fernandes[6,7], Stephanie Oliveira[1,2], Ana Rita Ferreira-Fernandes[1,2], Fernando Rodrigues[1,2], Joana Pereira-Sousa[1,2], Daniela Vilasboas-Campos[1,2], Sara Guerreiro[1,2], Jonas Campos[1,2], , Liliana Meireles-Costa[1,2], Cecilia M. Rodrigues[8,9], Stephanie Cabantous[10], Sergio F. Sousa[6,7], Manuela Lima[5,11], Andreia Teixeira-Castro[1,2], Patricia Maciel[1,2,*].

**Important Notice**:

One of the specificities of the design of our experiments that require Western Blots is that sample loading is made in sets of three (as we mostly have 3 experimental groups, WT, TG and TG TUDCA). However, the sample order is randomly chosen for each experiment. Therefore, each individual experiment has a different sample organization, which is indicated for each individual blot.
Purple rectangles indicate the blot shown in the main/supplemental figures.

# Figure 3B

Set No. 1

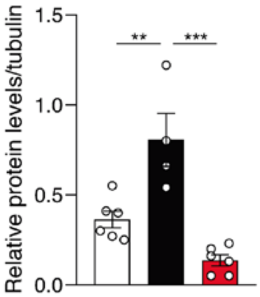


Samples are organized in sets of three, from left to right, as indicated below.

| TG | WT | TG TUDCA |
|---|---|---|

GFAP

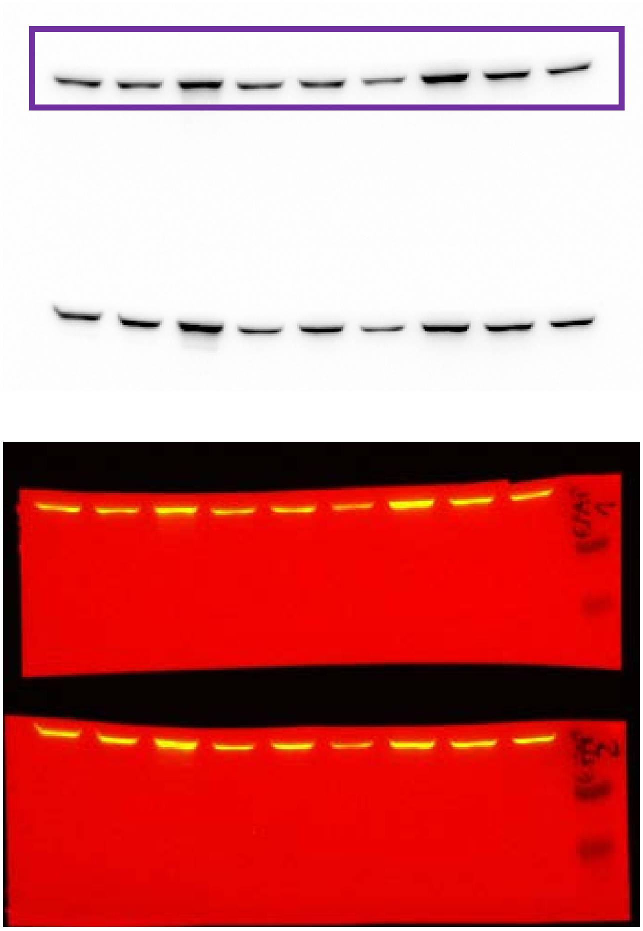

Tubulin

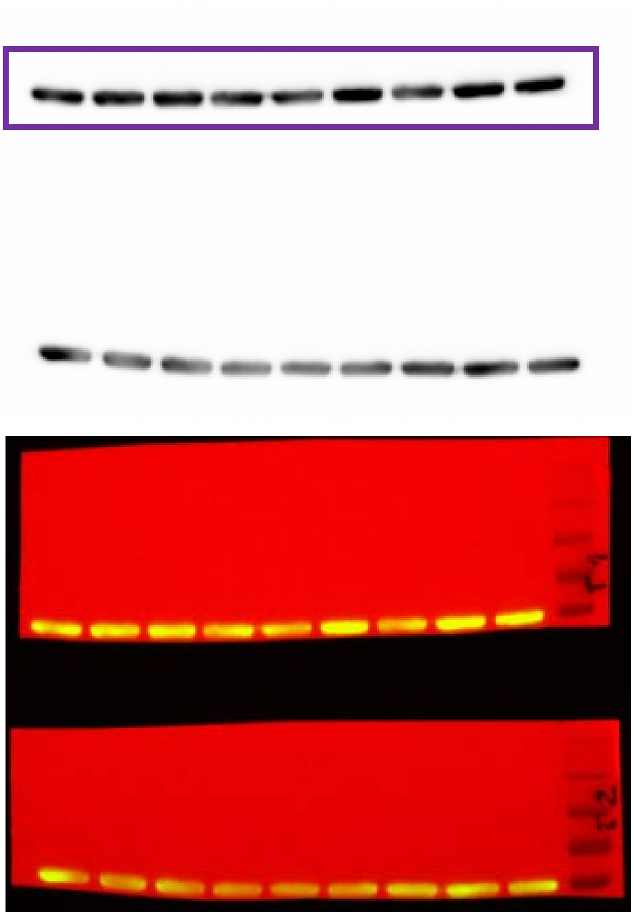

**Figure 3B**

| | | |
|---|---|---|
| WT | TG TUDCA | TG |

Set No. 2

Samples are organized in sets of three, from left to right, as indicated above.

**GFAP**

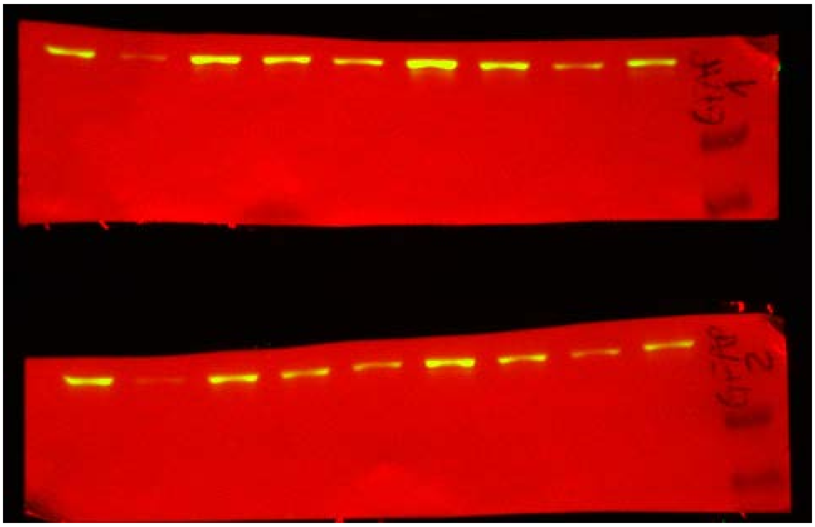

**Tubulin**

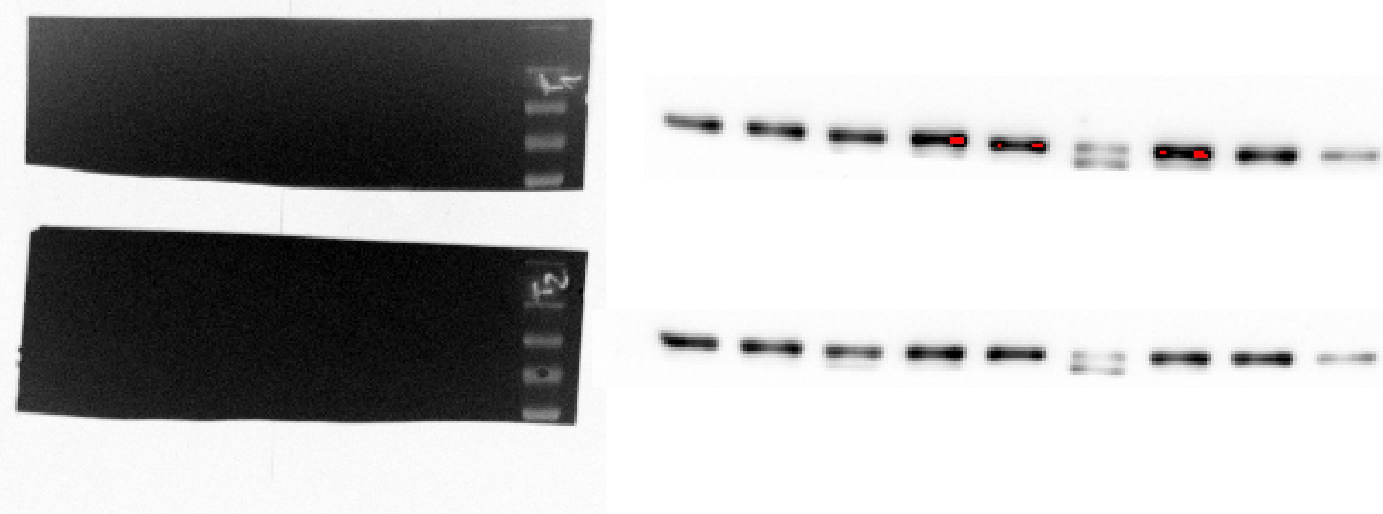

**Figure 6C**

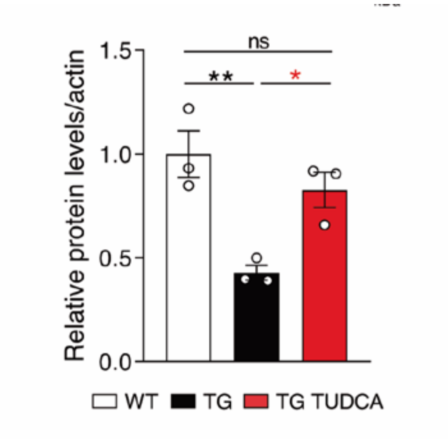


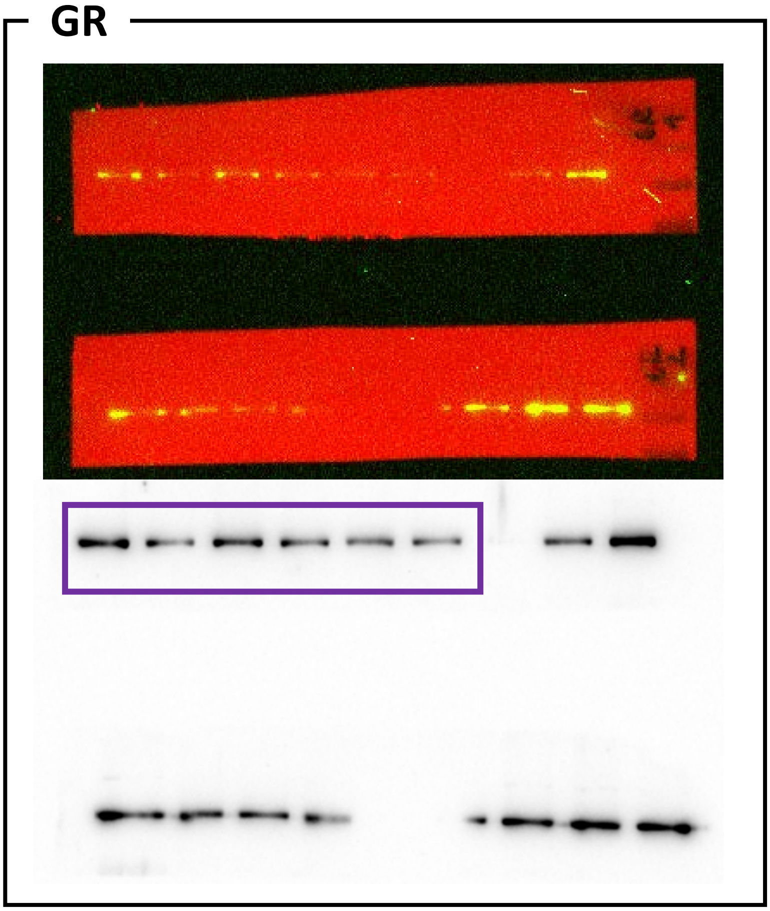


| WT | TG | TG TUDCA |
|---|---|---|

Samples are organized in sets of three, from left to right, as indicated above.

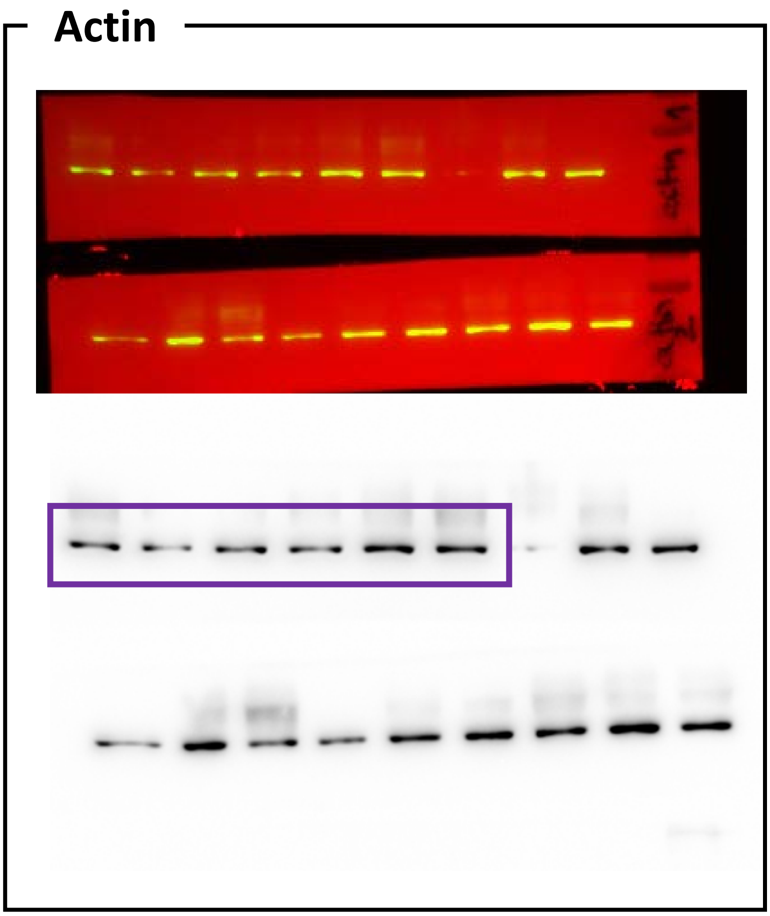

**Figure 6D**

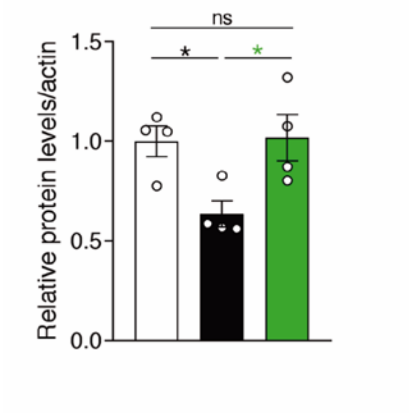


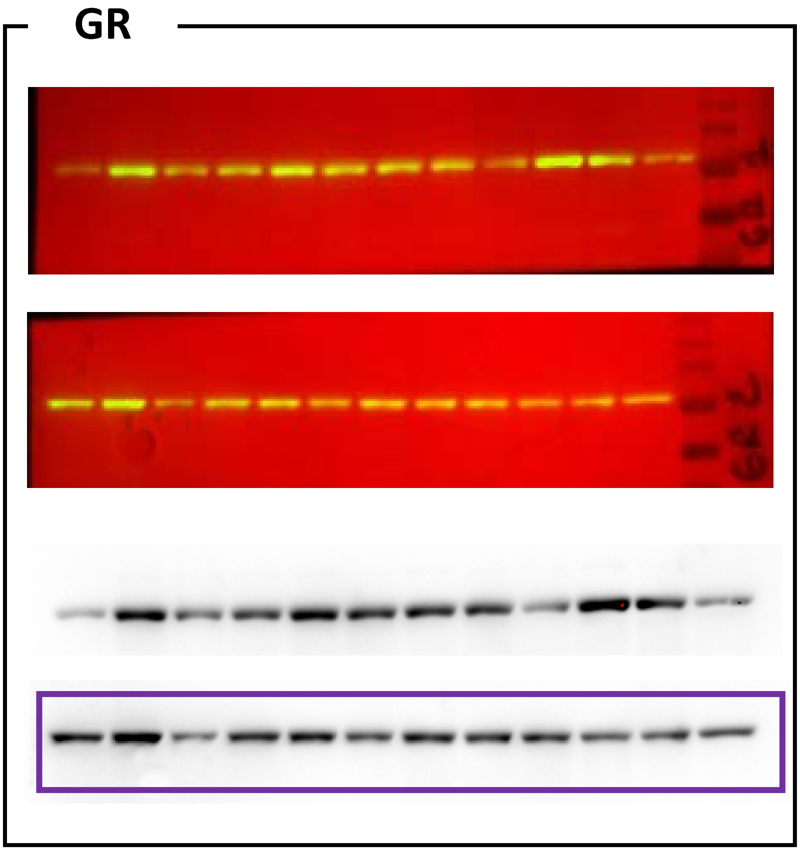


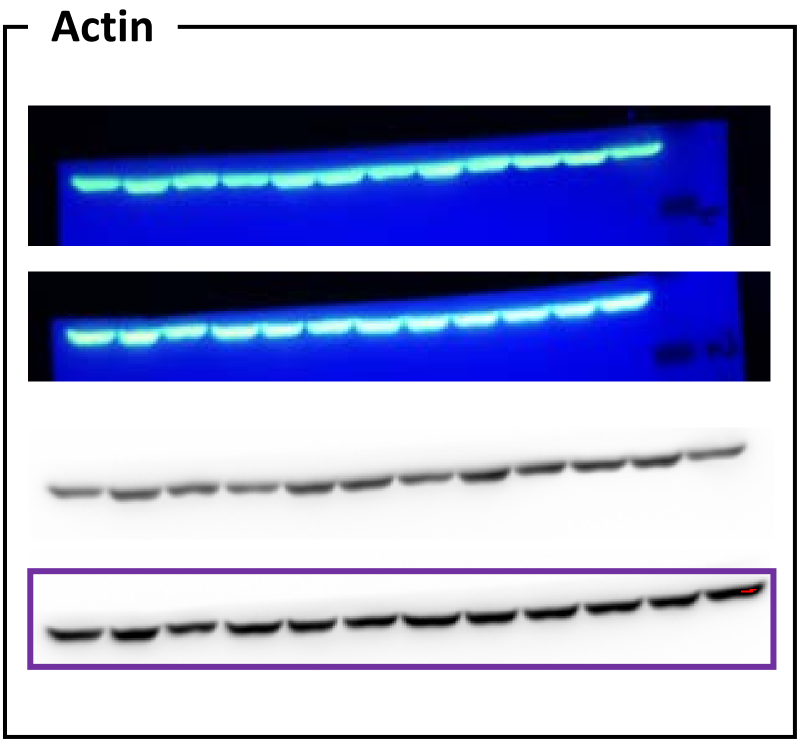


| | | |
|---|---|---|
| WT | TG TUDCA | TG |

Samples are organized in sets of three, from left to right, as indicated above.

# Figure 6E

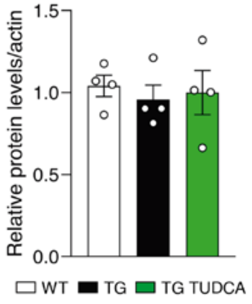


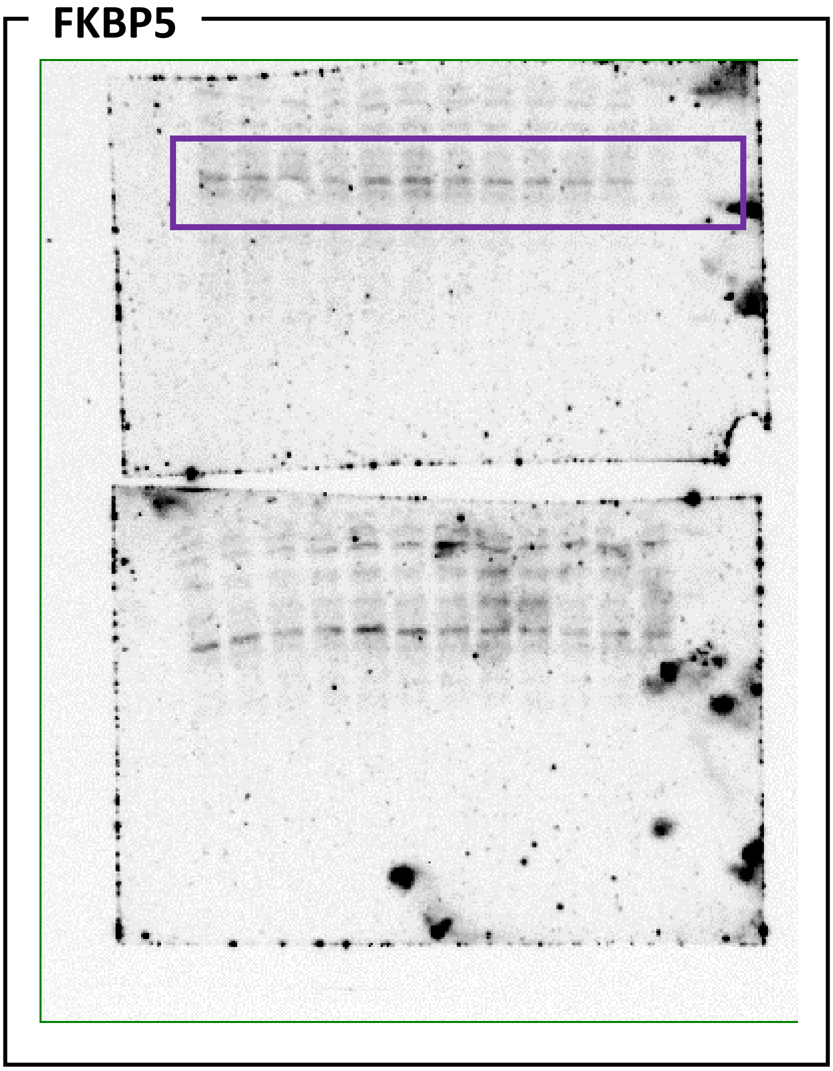


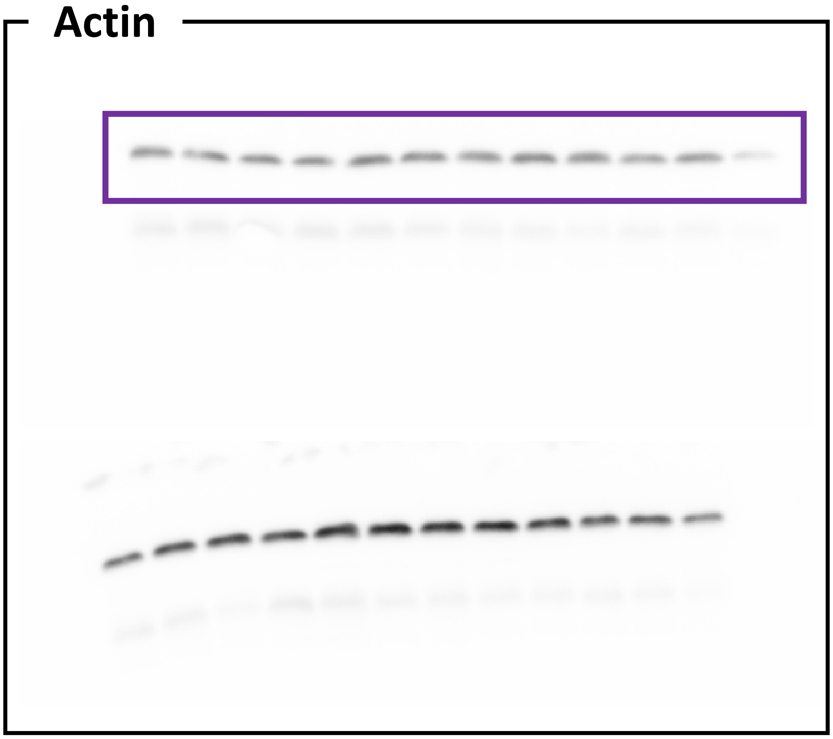


| TG | TG TUDCA | WT |
|---|---|---|

Samples are organized in sets of three, from left to right, as indicated above.

# Figure 6F

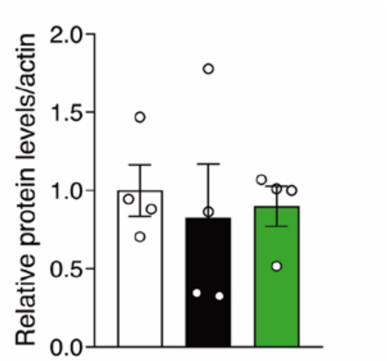


| WT | TG | TG TUDCA |
|---|---|---|

Samples are organized in sets of three, from left to right, as indicated above.

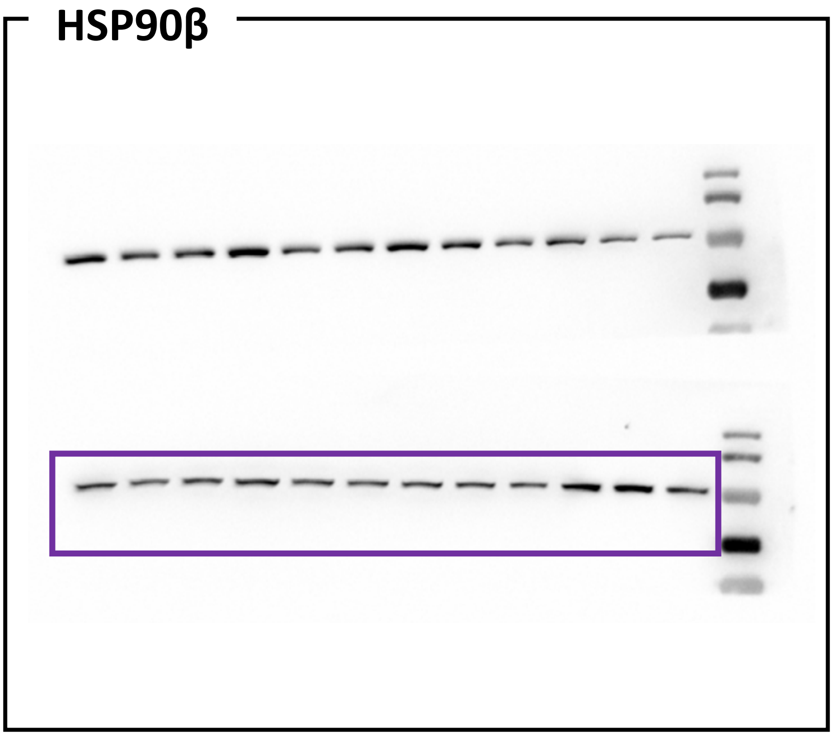


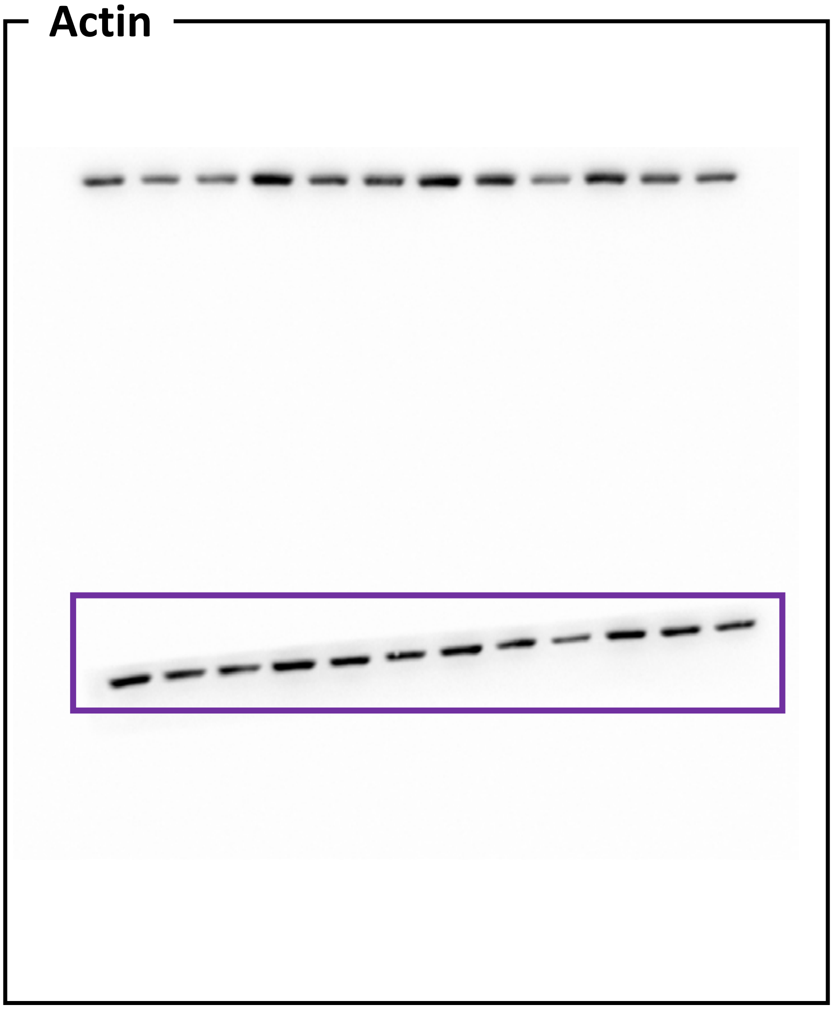

# Figure 6G

Set No. 1

(Single Group per Gel)

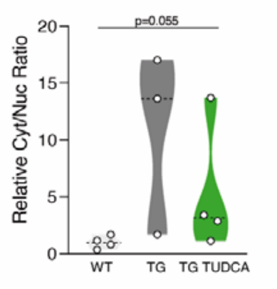


WT (1)

WT (2)

TG (1)

TG (2)

TG TUDCA (1)

TG TUDCA (2)

Four biological replicates of each group are included in each gel

## GR (Cytoplasmic)

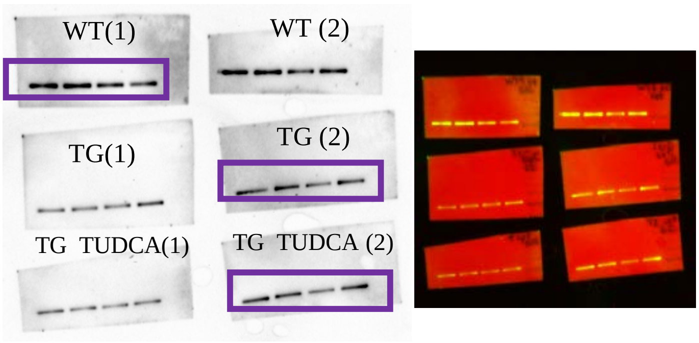


## Tubulin (Cytoplasmic)

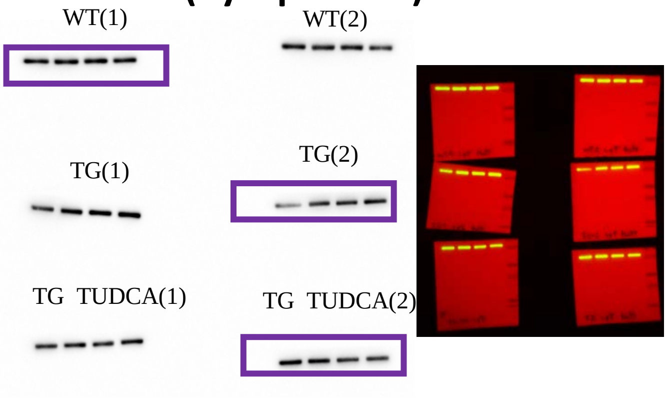


## GR (Nuclear)

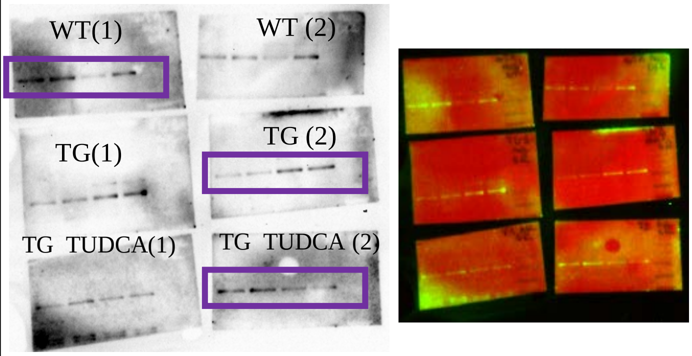


## H3 (Nuclear)

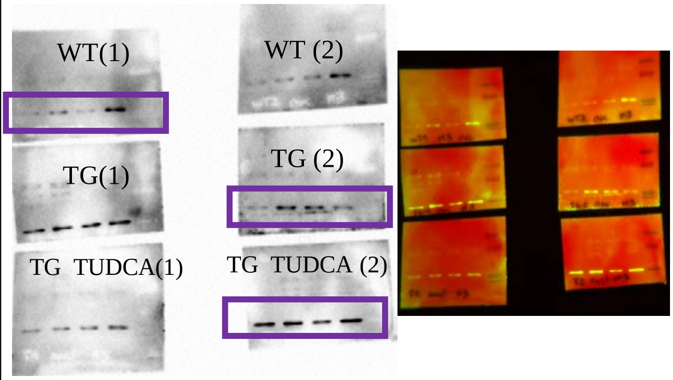

# Figure 6G

Set No. 2

(Single Group per Gel)

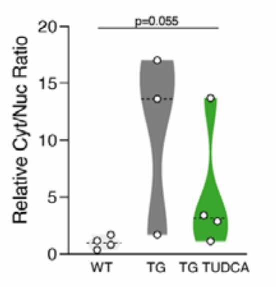


WT

TG

TG TUDCA

Four biological replicates of each group are included in each gel

## GR (Cytoplasmic)

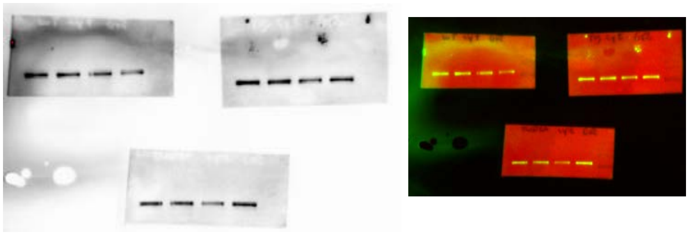

## Tubulin (Cytoplasmic)

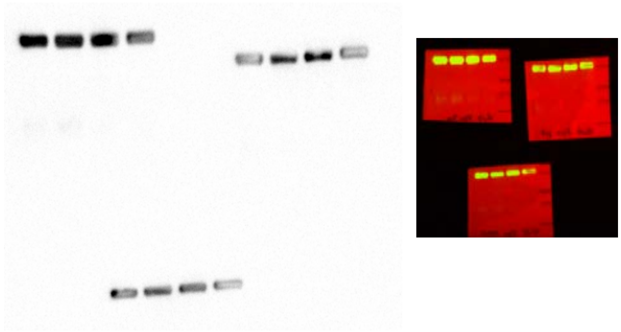

## GR (Nuclear)

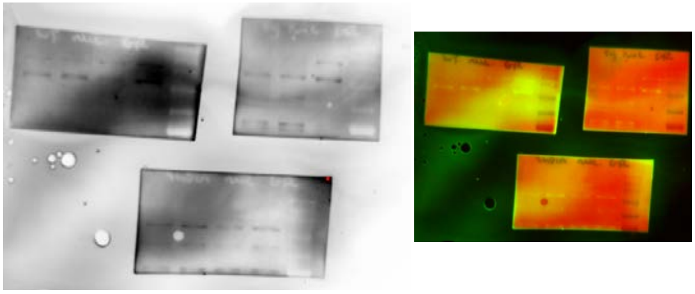

## H3 (Nuclear)

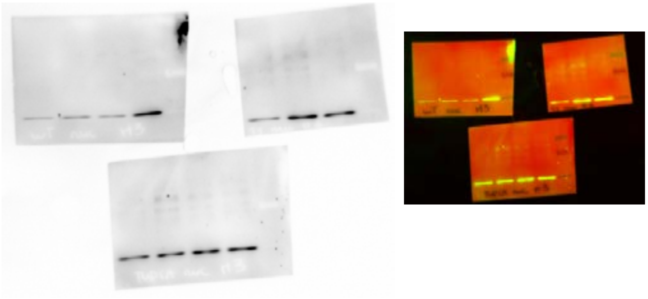

# Figure 6G

Set No. 3

(All Groups per Gel)

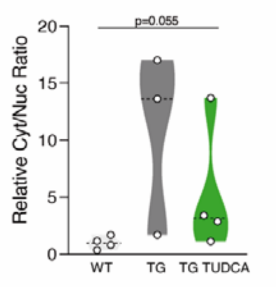


| WT | TG | TG TUDCA |
|---|---|---|

Samples are organized in sets of three, from left to right, as indicated above.

## GR (Cytoplasmic)

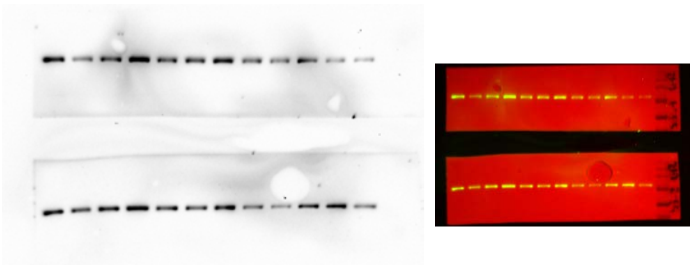

## Actin (Cytoplasmic)

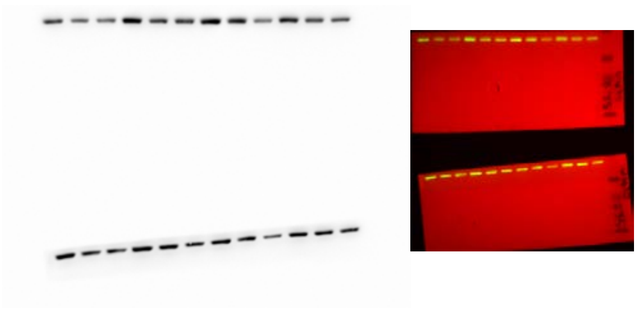

## GR (Nuclear)

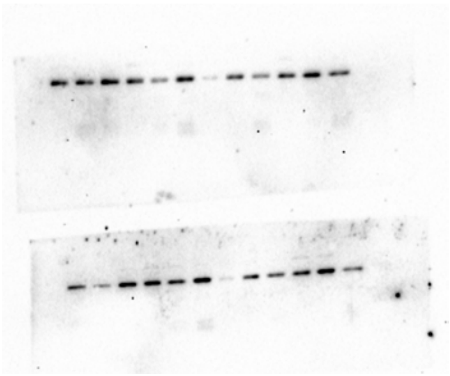

## H3 (Nuclear)

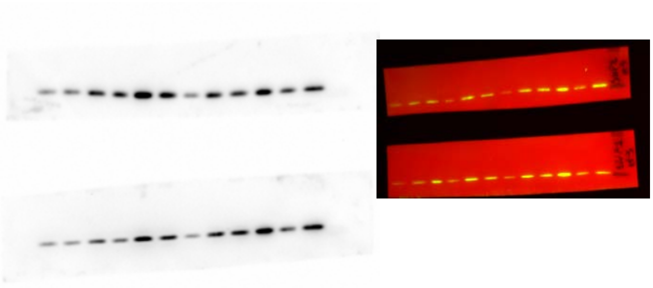

**Figure 6G**

# Efficiency of the Cellular Fractionation Protocol

Immunobloting of WT mouse brainstem after cellular fractionation of cytoplasm (showing the presence of actin and absence of H3) and nucleus (showing the presence of H3 and absence of actin)

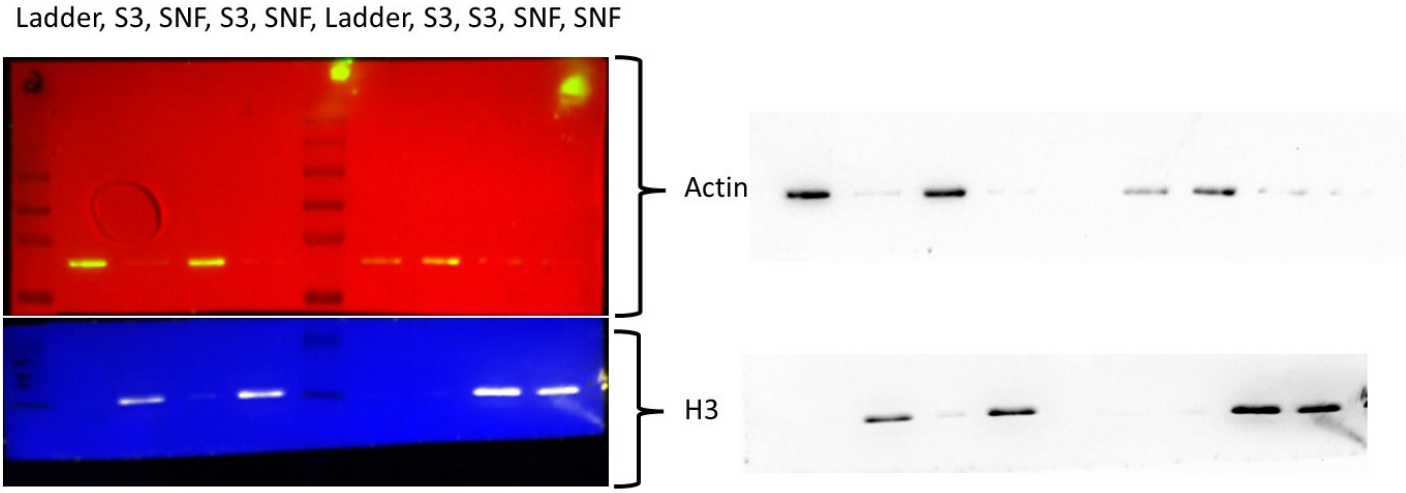


**S3 – cytoplasmic fraction**
**SNF – nuclear fraction**

**Figure 6H**

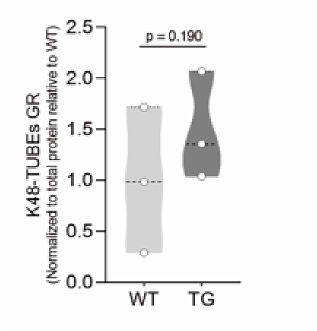


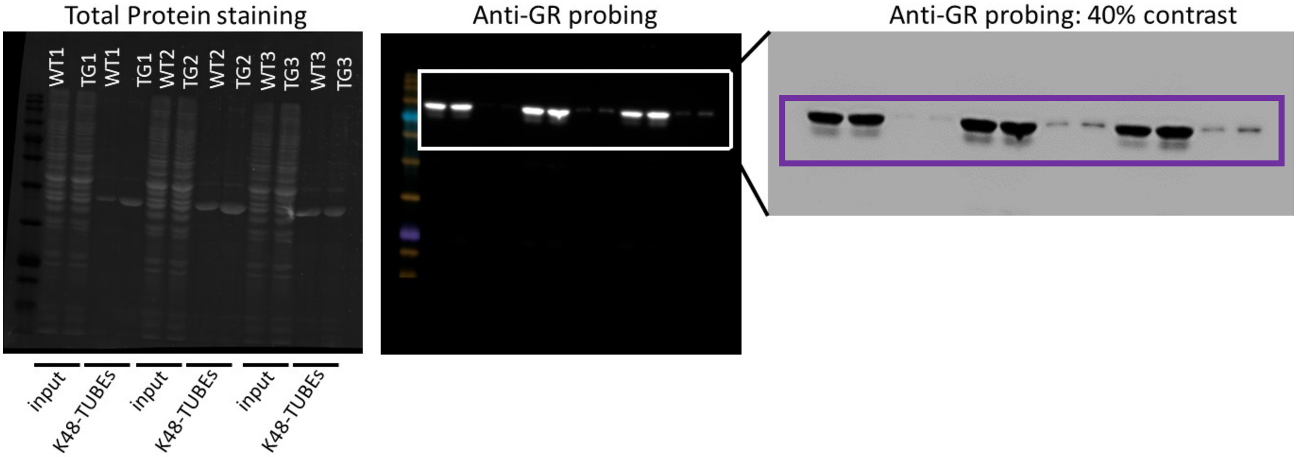


# Unbound fraction

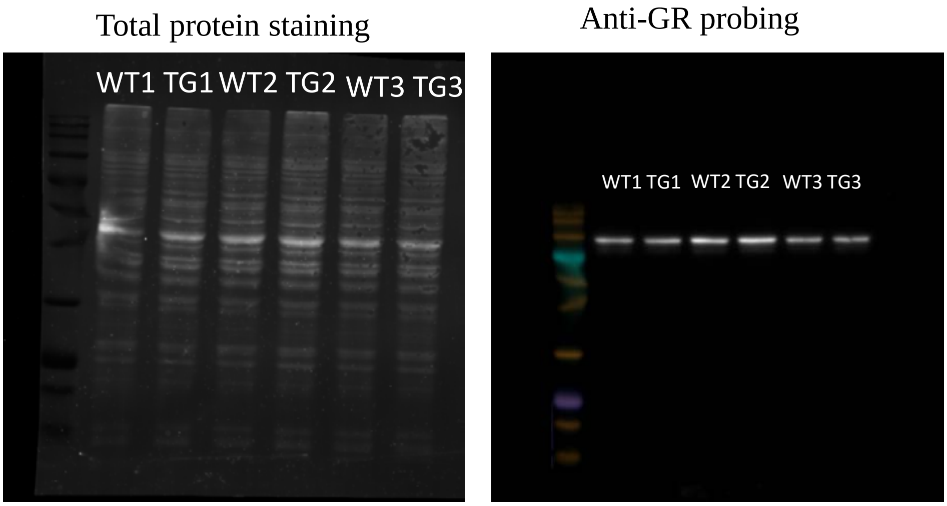

# Figure 7A – co-IP

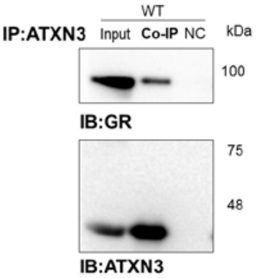


A 40% increase in image contrast was applied from the original blots

**Co-immunoprecipitation of ATXN3; Immunoblot with GR**

## Replicate 1

WT

Input Co-IP NC

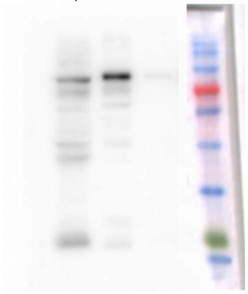

## Replicate 2

WT

Input Co-IP NC

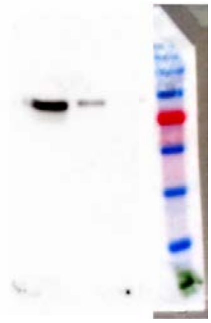

## Replicate 3

WT

Input Co-IP NC

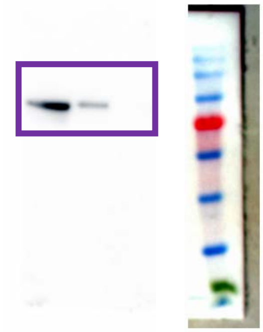

**Co-immunoprecipitation of ATXN3 (rabbit anti-MJD1); Immunoblot with ATXN3 (mouse anti-ATXN3 1H9)**

## Replicate 1

WT

Input Co-IP NC

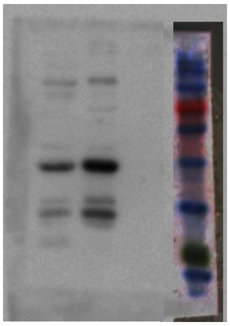

## Replicate 2

WT

Input Co-IP NC

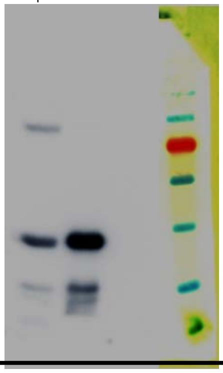

## Replicate 3

WT

Input Co-IP NC

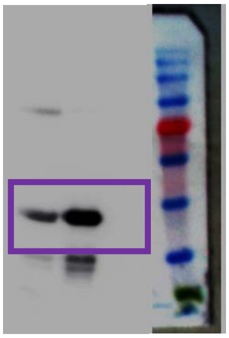

**Total protein staining**

## Replicate 1

WT

Input Co-IP NC

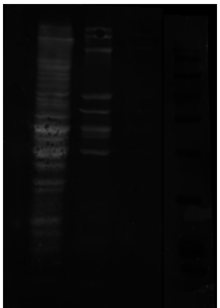

## Replicate 2

WT

Input Co-IP NC

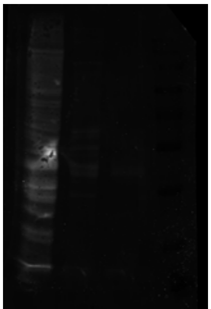

## Replicate 3

WT

Input Co-IP NC

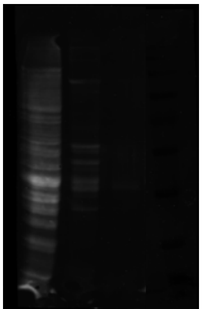

**Figure 8A**

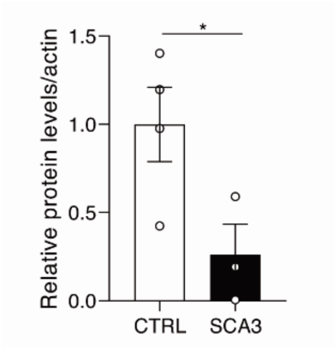


SCA3 | CTRL | SCA3 | CTRL | SCA3 | CTRL | CTRL

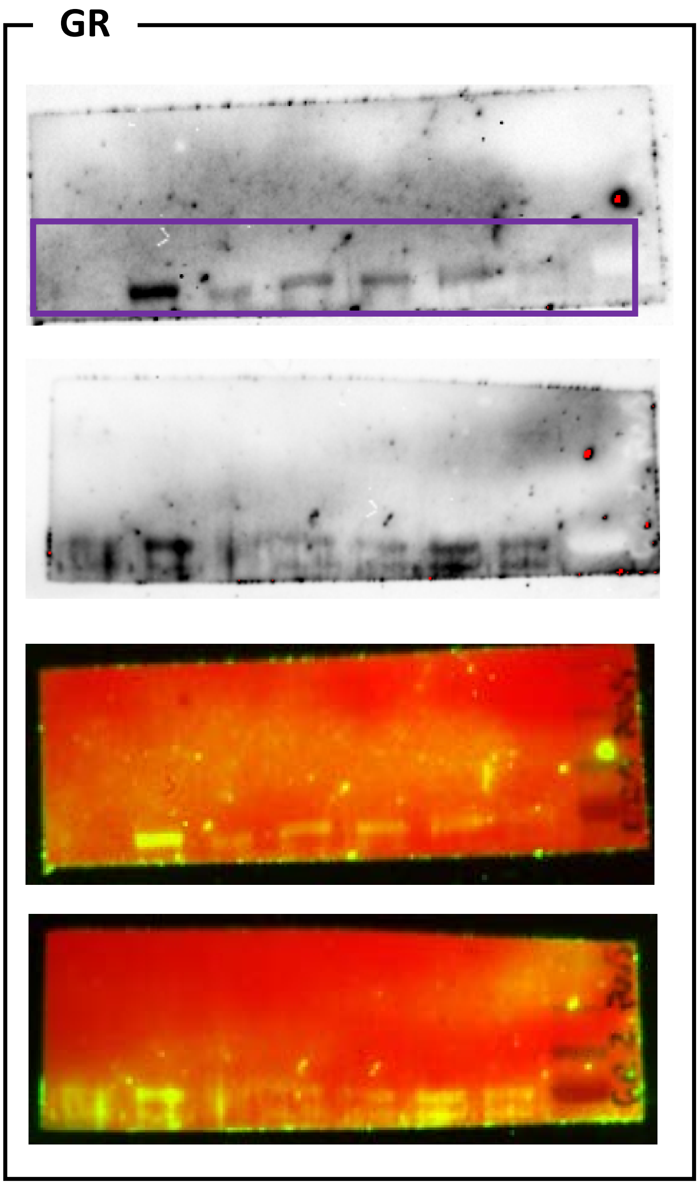


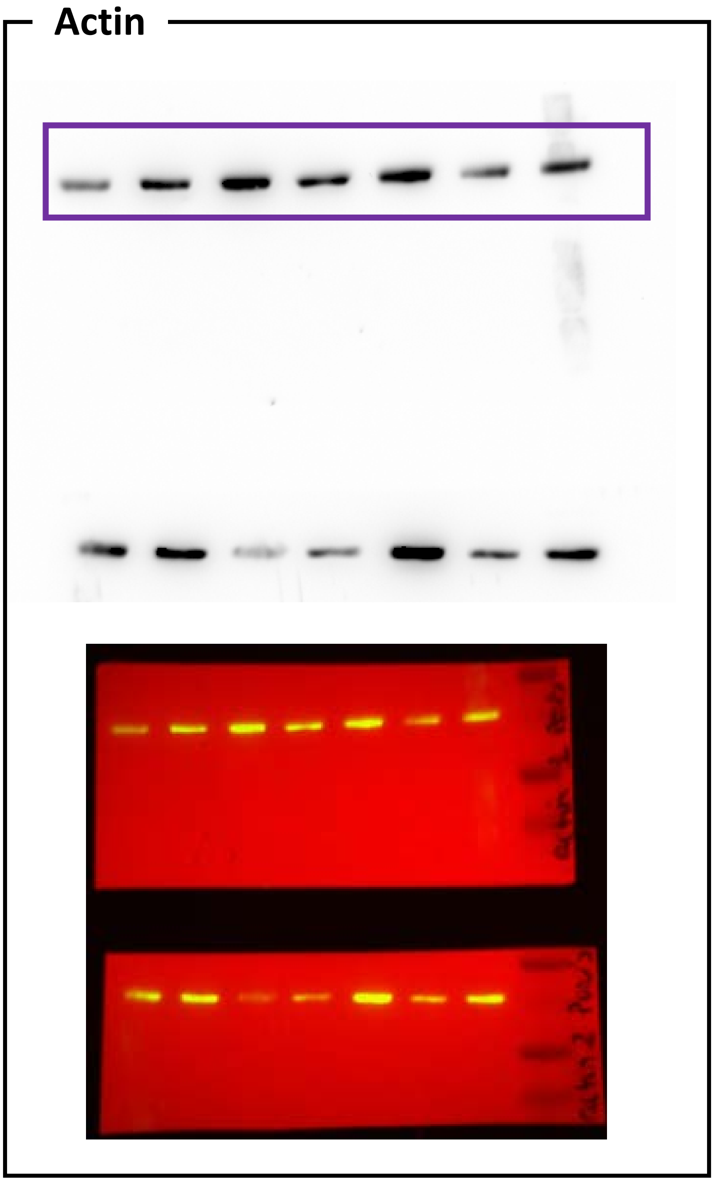

# Figure 8B

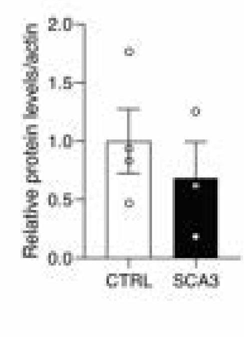


CTRL SCA3 CTRL SCA3 CTRL SCA3 CTRL CTRL

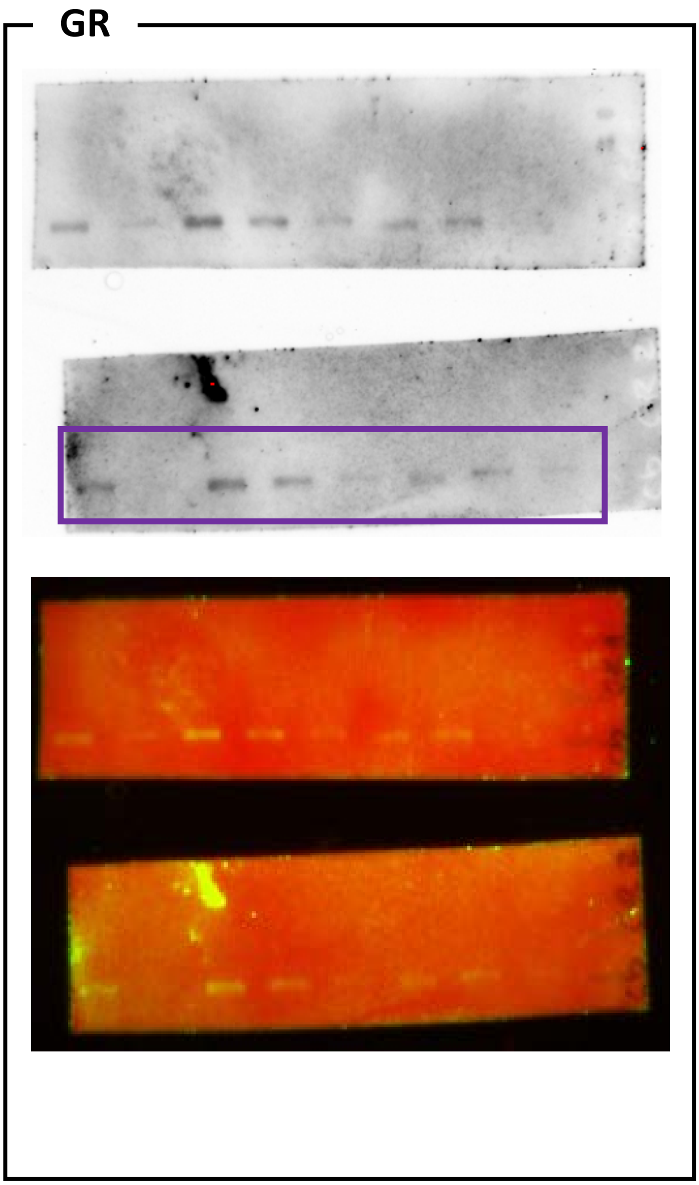


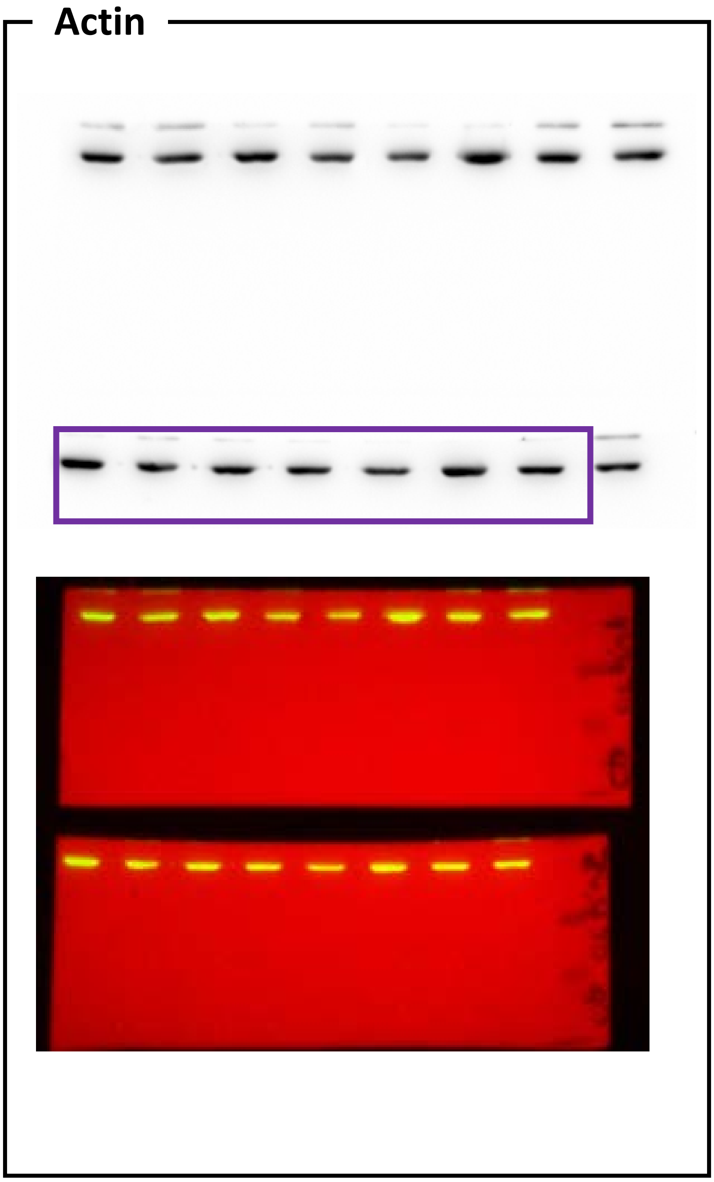

# Figure S6E

| WT | TG | TG TUDCA |
|---|---|---|

Samples are organized in sets of three, from left to right, as indicated above.

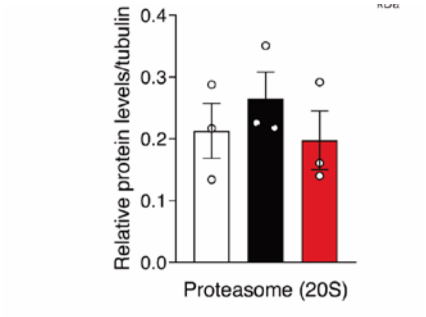


**Proteasome (20S)**

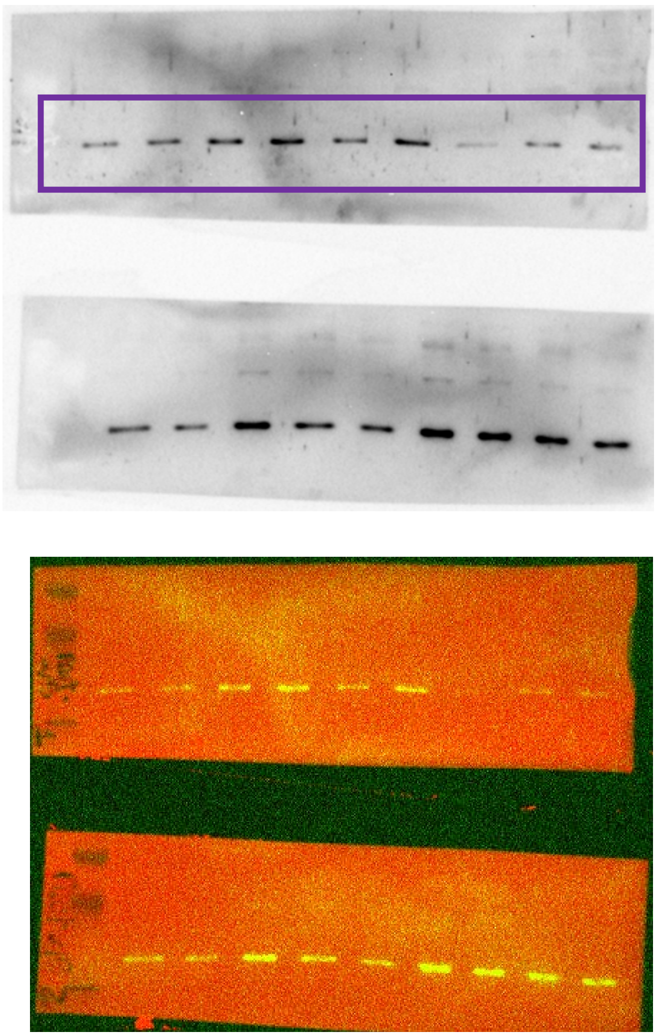

**Tubulin**

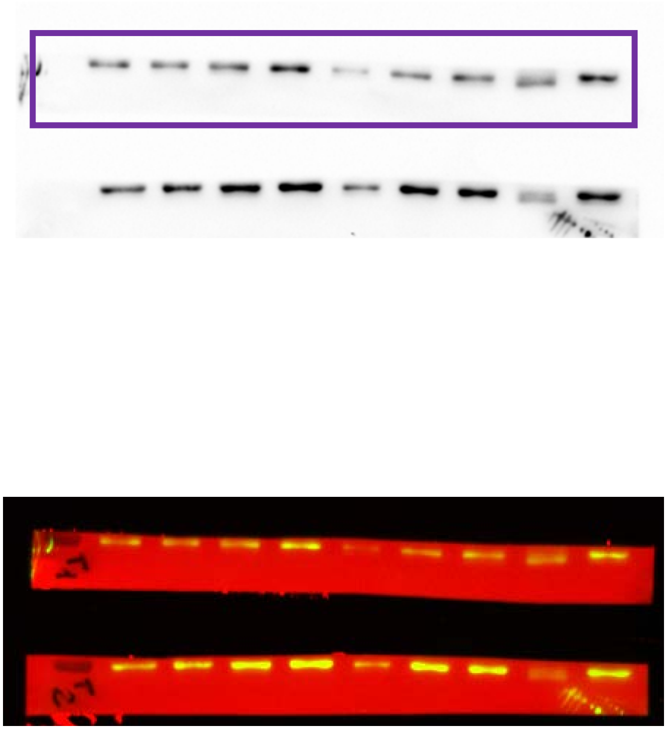

# Figure S7B

| WT | TG | TG TUDCA |
|---|---|---|

Samples are organized in sets of three, from left to right, as indicated above.

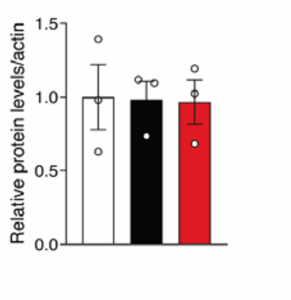


**IBA-1**

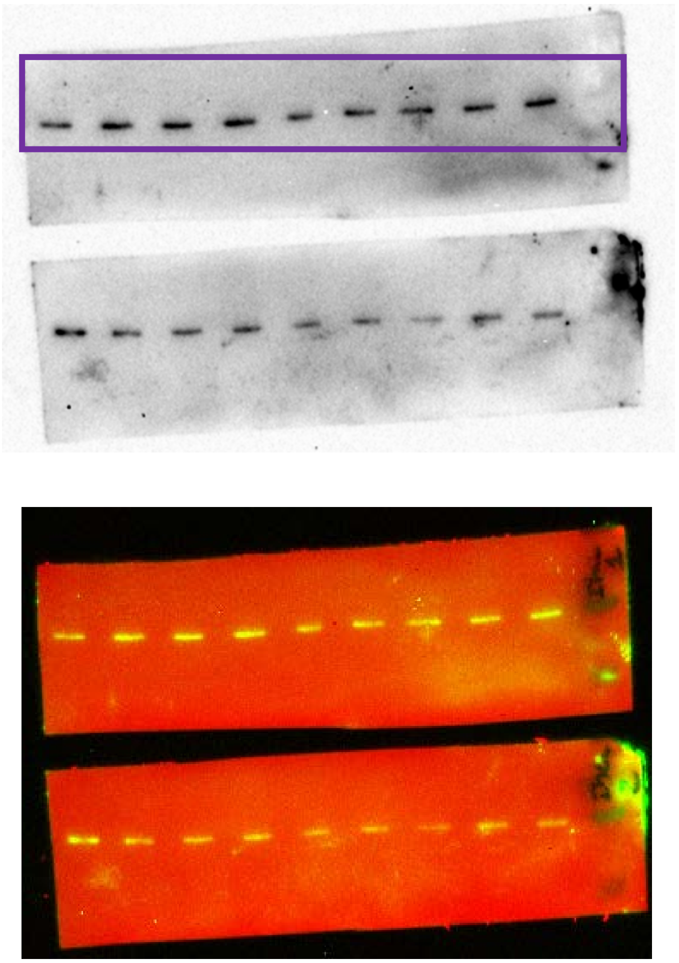

**Actin**

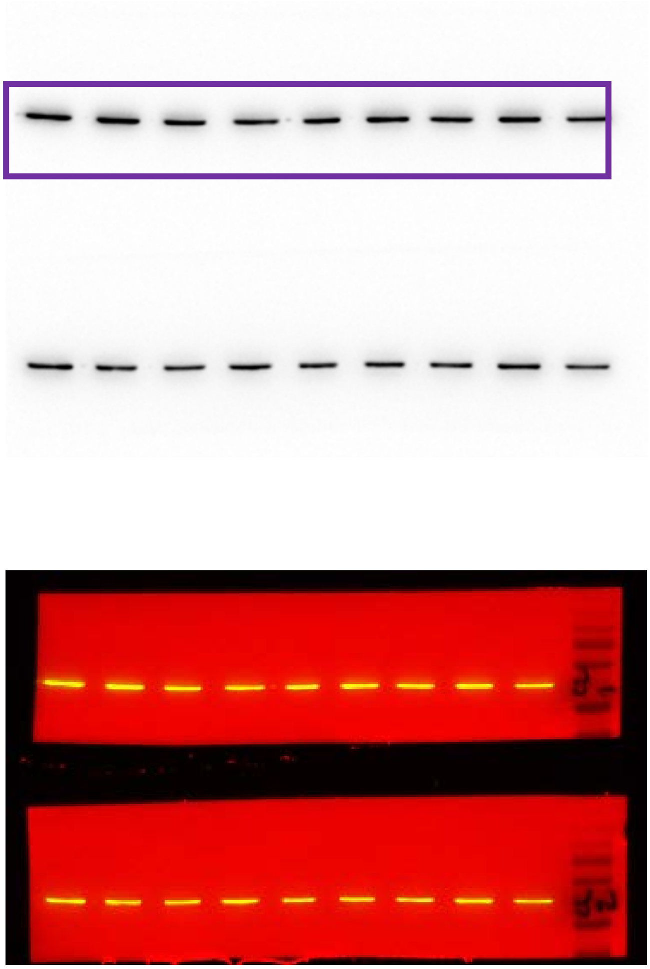

# Figure S7C

WT | TG | TG TUDCA

Samples are organized in sets of three, from left to right, as indicated above.

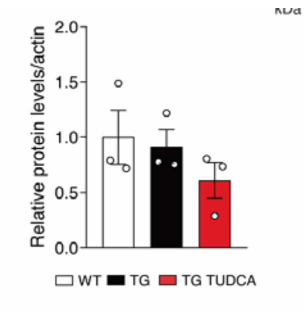


**IKKβ**

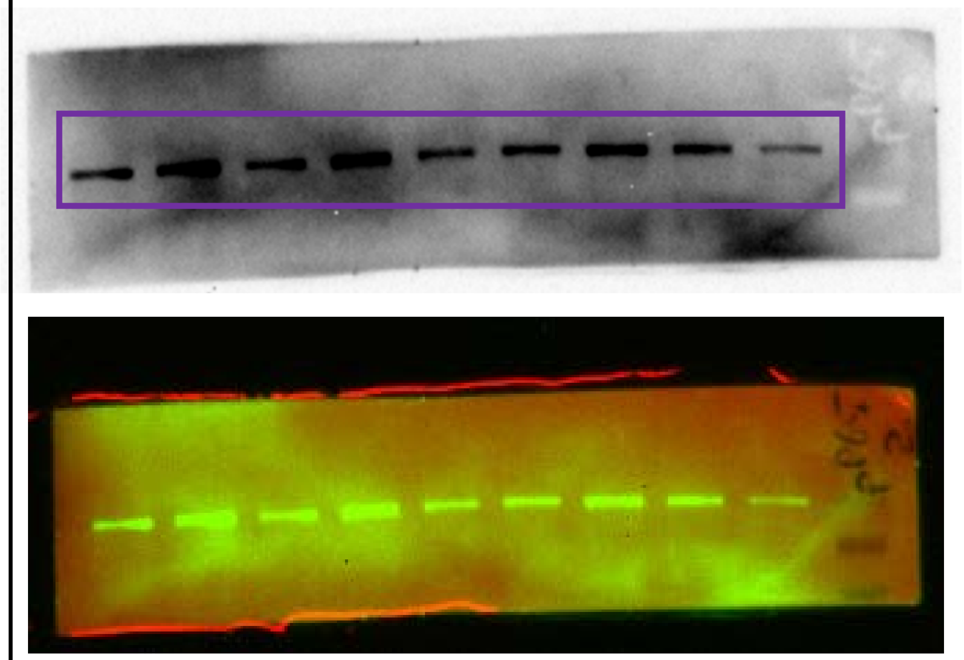

**Actin**

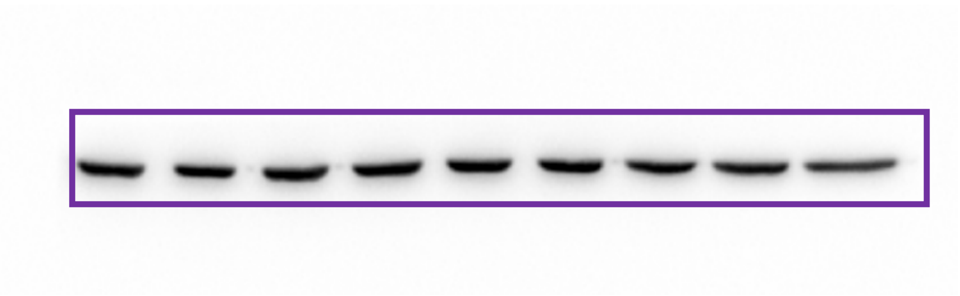

# Figure S7D

| WT | TG | TG TUDCA |
| --- | --- | --- |

Samples are organized in sets of three, from left to right, as indicated above.

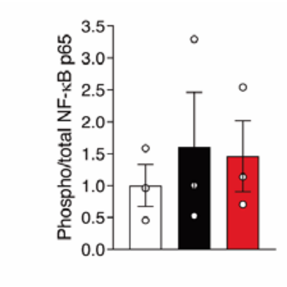


**P-p65**

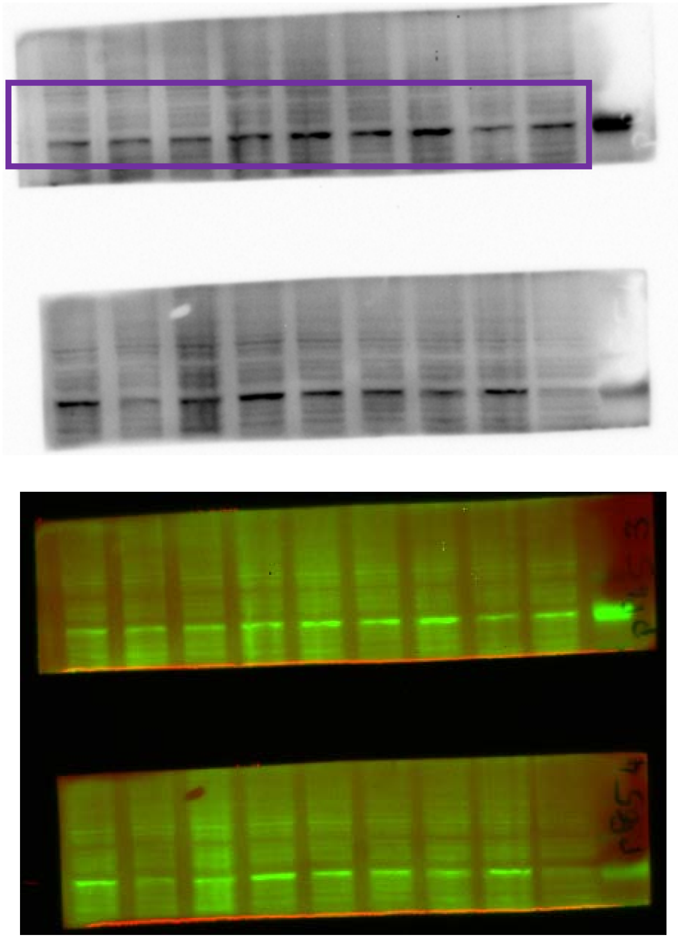

**Total p65**

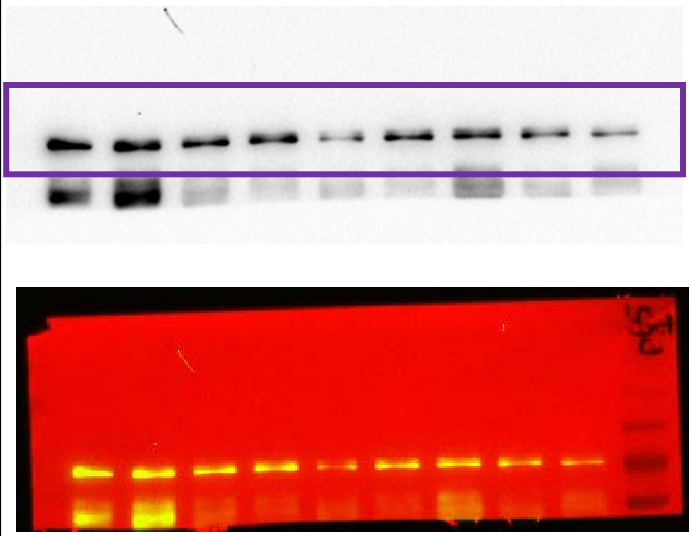

# Figure S7E

| WT | TG | TG TUDCA |
|---|---|---|

Samples are organized in sets of three, from left to right, as indicated above.

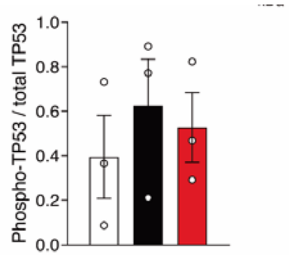


**P-TP53**

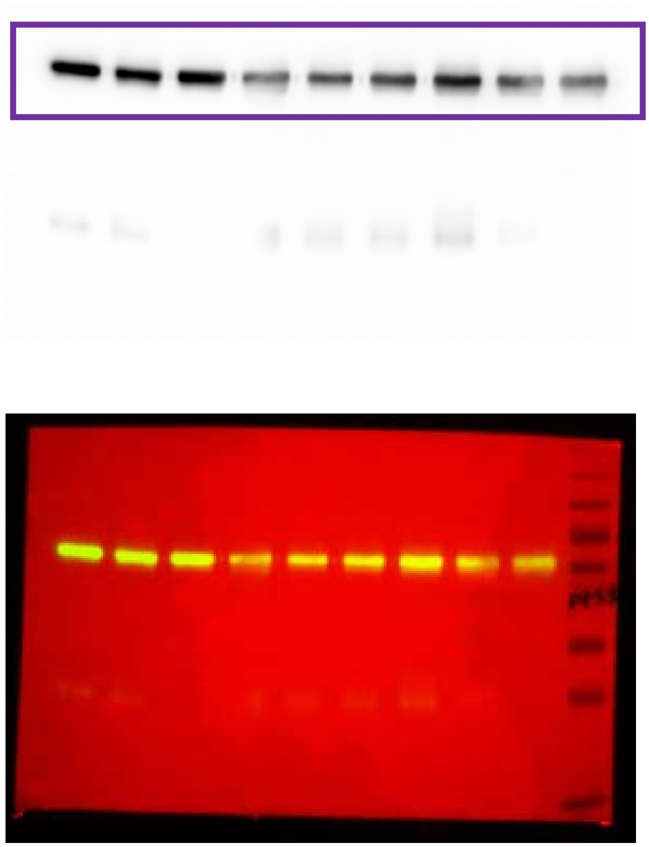

**Total TP53**

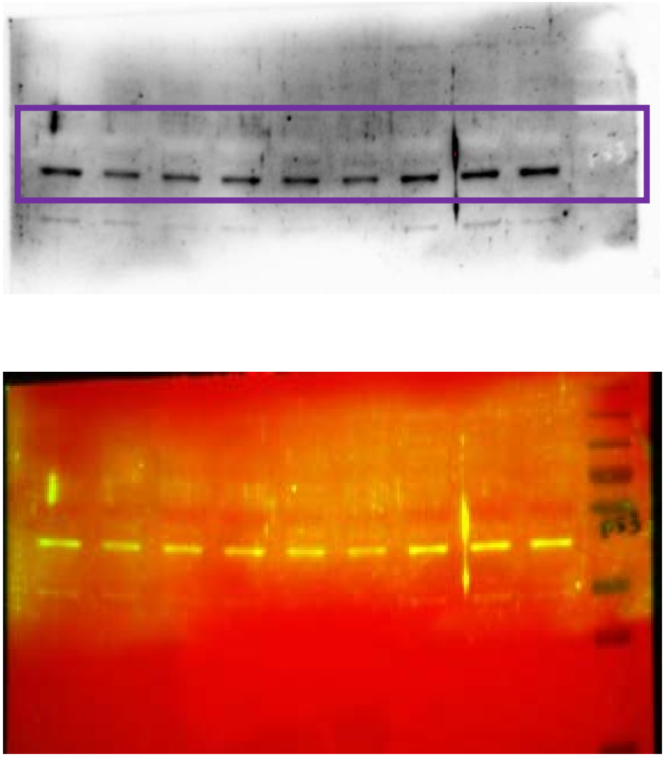

**Figure S9B**

| WT | TG | TG TUDCA |
|---|---|---|

Samples are organized in sets of three, from left to right, as indicated above.

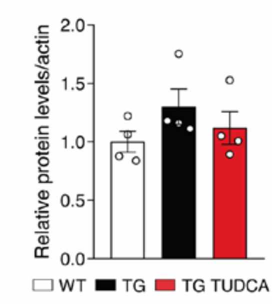


**GR**

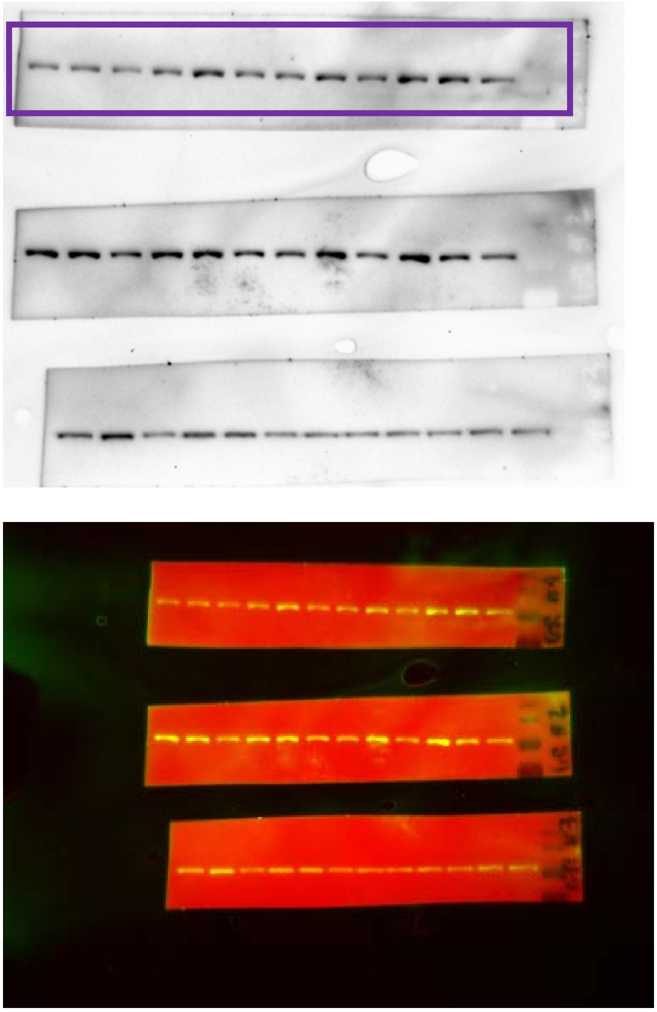

**Actin**

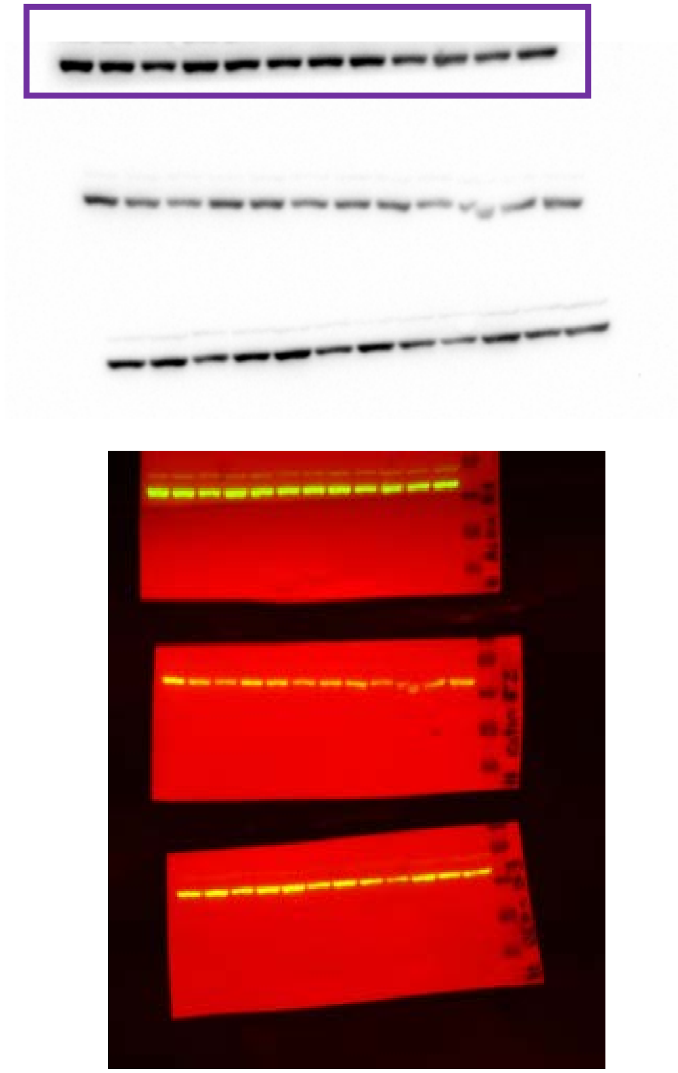

Supplement: Unedited blot and gel images [file jci-134-162246-s142.pdf]
